# Ethnopharmacology, Phytochemistry, and Global Distribution of Mangroves―A Comprehensive Review

**DOI:** 10.3390/md17040231

**Published:** 2019-04-18

**Authors:** Sadeer Nabeelah Bibi, Mahomoodally Mohamad Fawzi, Zengin Gokhan, Jeewon Rajesh, Nazurally Nadeem, Rengasamy Kannan R.R., Albuquerque R.D.D.G., Shunmugiah Karutha Pandian

**Affiliations:** 1 Department of Health Sciences, Faculty of Science, University of Mauritius, Réduit 80835, Mauritius; nabeelah.sadeer1@umail.uom.ac.mu (S.N.B.); r.jeewon@uom.ac.mu (J.R.); 2 Department of Biology, Science Faculty, Selcuk University, Campus, 42250 Konya, Turkey; gokhanzengin@selcuk.edu.tr; 3 Department of Agricultural and Food Science, Faculty of Agriculture, University of Mauritius, Réduit 80835, Mauritius; n.nazurally@uom.ac.mu; 4 Department of Biotechnology, Science Campus, Alagappa University, Karaikudi 630 003, India; 5 Universidade Federal do Rio de Janeiro (UFRJ), Rio de Janeiro, Brazil; ricardo-diego-cf@hotmail.com

**Keywords:** bioactive compounds, *Bruguiera gymnorhiza*, *Rhizophora mucronata*, *Avicennia* species, pneumatophores, traditional uses

## Abstract

Mangroves are ecologically important plants in marine habitats that occupy the coastlines of many countries. In addition to their key ecological importance, various parts of mangroves are widely used in folklore medicine and claimed to effectively manage a panoply of human pathologies. To date, no comprehensive attempt has been made to compile and critically analyze the published literature in light of its ethnopharmacological uses. This review aims to provide a comprehensive account of the morphological characteristics, ethnobotany, global distribution, taxonomy, ethnopharmacology, phytochemical profiles, and pharmacological activities of traditionally used mangroves. Out of 84 mangrove species, only 27 species were found to be traditionally used, however not all of them are pharmacologically validated. The most common pharmacological activities reported were antioxidant, antimicrobial, and antidiabetic properties. Mangroves traditionally reported against ulcers have not been extensively validated for possible pharmacological properties. Terpenoids, tannins, steroids, alkaloids, flavonoids, and saponins were the main classes of phytochemicals isolated from mangroves. Given that mangroves have huge potential for a wide array of medicinal products and drug discovery to prevent and treat many diseases, there is a dire need for careful investigations substantiated with accurate scientific and clinical evidence to ensure safety and efficient use of these plants and validate their pharmacological properties and toxicity.

## 1. Introduction

Medicinal plants are potential pharmacies grown in the wild and have been co-existed and co-evolved alongside human civilizations since the beginning of life on Earth. Since ancient times, human life has been revolving around plants as they were used for their curative nature to alleviate human pain and have been the focal point of many researchers since the dawn of medicine. For centuries, medicinal plants have been used as remedies for human ailments and diseases because they contain components of therapeutic value. With the increasing incidence and complexity of diseases threatening human health, the need for novel and effective bio-molecules is of paramount importance, which brings forward natural products/plants as the pipeline of tomorrow for drug discovery. Alarmingly, recent estimates reported that every fifth plant species found under the kingdom Plantae are threatened with extinction [1] and thus if we are not careful, they may disappear in front of our eyes due to disastrous environmental factors taking with them notable medicinal values.

Due to the long history in folklore medicine, medicinal plants have not escaped the attention of today’s pharmaceutical chemists. The importance of traditional medicines has been well understood by the pharmaceutical industry since the discovery and successful development of aspirin from the symbolic Willow tree [2]. For instance, metformin, derived from *Galega officinalis* L., is a commonly used type 2 diabetic drug. Interestingly, a study has shown that metformin can also have potential cytotoxic effects on cancerous cells [3]. Taxol, the blockbuster anticancer drug, derived from *Taxus brevifolia* Nutt., showed significant effect against various types of cancers viz; ovarian, breast, lung cancer, head, and neck tumors [4]. Medicinal plants have contributed profoundly in the discovery of new compounds, and the quest is still ongoing with the aim to search for more novel biologically active metabolites from traditionally used medicinal plants.

At the time of writing, Allkin Bob from the Royal Botanic Gardens, Kew, recorded around 28, 187 plant species as medicinal plants [5]. Many of them are commonly known in the medical lore and are also extensively used in modern phytomedicine while some of them still need a thorough investigation. This review aims at elaborating and providing an overview on mangrove plants, which are traditionally known medicinal plants and have attracted much interest in the quest for novel pharmacophores.

Mangrove is a shrub or small tree that grows in coastal brackish or saline waters in muddy or rocky soils. Mangroves are halophytes, being salt tolerant, they can quickly adapt themselves in harsh coastal conditions [6]. Currently, the word ‘mangrove’ encompasses 84 species from 24 genera and 16 families. However, only 70 species out of the 84 are classified as true mangroves while the rest as mangrove associates [7]. Nonetheless, the difference between these two classifications is still unclear which can lead to misinterpretations. Irrespective of the classification issues, many mangrove trees are traditionally used, and several genera have attracted the attention of many scientists, particularly the genera *Rhizophora, Bruguiera*, and *Avicennia*.

Several species of mangroves have been traditionally used against a plethora of diseases. Mangroves such as *Bruguiera gymnorhiza* (L.) Lam, *Rhizophora mucronata* Lam, and *Acanthus ilicifolius* L. have been recognized as the three most traditionally used mangrove species. Several in vivo and in vitro studies have been conducted on many mangrove species. For instance, *Avicennia germinans* (L.) L. showed anti-ulcer activity, whereas *B. gymnorhiza* has been reported for significant antioxidant, antidiabetic, and anti-inflammatory activities. *Rhizophora apiculata* Blume was screened for a wide array of pharmacological activities viz; antioxidant and antimicrobial properties. *R. mucronata* covered a broader spectrum of biological activities, namely antidiabetic (in vivo and in vitro), antioxidant, anti-inflammatory, antimicrobial, analgesic, anti-HIV, and anticholinesterase activities. Phytochemical screenings were also conducted on various species confirming the presence of tannins, alkaloids, and steroids among others.

In terms of distribution, Indonesia is the primary source of mangroves occupying the most significant area globally [8]. These plants form a rare and unique ecosystem but are threatened since they are destroyed five times faster than tropical forests [9]. For instance, North and Central America are recognized as the most threatened mangrove regions due to coastal development, hurricanes, and aquaculture. Aquaculturing of shrimps, mud crabs, or oysters is a critical staple job for many people in Southeast Asia. However, aquaculture is recognised as a leading threat to mangroves [10]. It is considered that 90% of the mangrove forests are found in developing countries which consequently build a thin line between livelihoods and mangroves [9]. People make a living on mangroves through fishing. Achim Steiner, head of the UN environment program, mentioned that mangroves contribute to the economy for a value of $57,000 per hectare annually [9].

It is increasingly acknowledged that mangrove plants are rich in natural products and new chemical compounds. Mangroves have been given a considerable extent of scientific importance worldwide as they are known for their potent activity against many diseases namely cardiovascular disease, diabetes, hypertension, and cancer. Many studies have probed into the pharmacological aspects of different mangrove species, and a wealth of literature has already emerged and published. Attempts have been made previously to validate the traditional uses of several mangrove trees using in vitro and in vivo models. Nonetheless, reports are scanty on the ethnopharmacological uses of mangroves. Thus, this review aims to provide a comprehensive insight into the morphological characteristics, ethnobotany, global distribution, taxonomy, ethnopharmacology, phytochemical profiles, and pharmacological activities of traditionally used mangroves. In addition, primary data has been analyzed to (i) compare species that were medical lore, (ii) highlight the main countries using mangroves species as a traditional remedy, (iii) compare the types of extracts, plant parts, and assays used in pharmacological validation of species, and (iv) highlight the main compounds isolated from traditionally used mangroves.

## 2. Review Methodology

Relevant literature was collected by probing scientific electronic databases namely EBSCO, Google Scholar, PubMed, and ScienceDirect and web sources such as PROSEA, PlantNET, and The Plant List. Keywords such as the different mangrove species, traditional uses, ethnobotany, ethnopharmacology, pharmacological activities, morphological characteristics, and phytochemistry were used. The manual search of ethnobotanical textbooks and related compilations were also made. Two articles in non-English languages (Persian and Thai) were also included. Information was gathered and summarized in the forms of tables wherever appropriate. For instance, Table 1 shows the local names used in countries. Table 2 distinguishes between the three dominant types of mangroves. Table 3 shows the first 20 nations with mangrove plantation. Table 4 describes the morphological characteristics of the different mangroves species as well as giving information on which family and taxonomic rank they belong to. Table 5 describes the traditional uses concerning the different parts of the mangrove plants together with information on their corresponding country of origin. Table 6 shows which mangrove species are traditionally and pharmacologically validated. Table 7 summarizes the in vivo and in vitro assays including the different types of tests done, parts of the plants used, and biological activities on both extracts and controls. Table 8 summarizes the phytochemical compounds isolated from each mangrove species. 

## 3. Terms, Origin, and Definition

The term ‘mangrove’ is of Guarani origin, the official language of Paraguay. In the early 1610s, the word was spelled as ‘mangrow’ coming from Portuguese mangue or Spanish ‘mangle’, but later in the 1690s the term ‘mangrow’ turned into an English word as ‘mangrove’ via folk etymology. Mangroves are associated with many terms, namely mangrove forest community or mangal and mangrove ecosystem. Other terms synonymous to mangrove forest are tidal forest, coastland woodlands, mangrove swamp, tidal swamp forest, and oceanic rainforests [6,11]. For instance, the mangrove forest community or mangal is linked with microbes and fungi while animals associated with the plants form the mangrove ecosystem [12]. It is suggested that the word ‘mangrove’ should be referred to specific mangrove species while the word ‘mangal’ to the forest community instead. Table 1 represents the local names used in different countries.

The discovery of mangroves happened during the time of Alexander III of Macedon commonly known as Alexander the Great from 326–324 B.C. During Alexander’s Indian expedition in 325 B.C., Nearchus (admiral of Alexander the Great’s army) was ordered to sail along the shores of Indus River to the Euphrates passing through the Persian Gulf. It was during the expedition that Nearchus made the first discovery of the plant ‘Mangrove’. Later in 305 B.C., a Greek philosopher Theophrastus also reported and documented the existence of the mangrove vegetation in his book entitled as “Historia Plantarum” [6,14,15]. As a result, it is recognized that the most ancient written shreds of evidence on mangroves were documented by Nearchus and Theophrastus. Both described the plants as ‘held up by their roots like a polyp’, and the leaves and flowers were *Rhizophora* [12,14]. Consequently, in 323 B.C., the ancient Greeks became aware of three mangrove areas namely the Red Sea, the Arabian Sea, and the Persian Gulf [15].

Many tribes and indigenous people have relied heavily on mangroves as a source of raw material and medicines. For instance, the tribal group of people living in the Orinoco Delta in Venezuela known as the Warao people has been dependent on mangrove forests for approximately 7000 years. The Warao people also known as the mangrove people used the roots of these plants to build houses and boats. Thousands of years later, mangroves attracted more people across the globe and till date has maintained its valuable importance for many people and also animals [14]. Recently, a study conducted by Gardner in Madagascar showed that lemurs use mangroves as their natural habitats for sleeping and foraging [16].

For many years, mangroves have formed remarkable and highly prolific ecosystems along the coastlines of many countries around the world that are both environmentally and medicinally important [14]. Within the scope of knowledge, mangroves originate from the Indo-Malayan regions which grow most of the mangrove species around the world with Indonesia being the first country covering the most extensive mangrove area globally (Table 2) [8,17]. The propagules and seeds produced by the plants have a unique feature which helped them to float in the water. Due to this characteristic, it was easy for the mangrove species to spread by water dispersal to Central and South America through India, East Africa about 23–66 million years ago [17].

Morphologically, a mangrove is a shrub or small tree that grows in coastal brackish or saline waters in muddy or rocky soils. Mangroves are halophytes as they are salt tolerant and are easily adapted to harsh coastal conditions due to their buttress root system or rhizophores and their aerial roots or pneumatophores [6]. Different sources defined mangroves differently. For instance, Collins dictionary defined mangrove plants as a tree growing along the coastlines or on the bank of river in tropical countries [18] while Merriam-Webster [19] defined the plant as ‘any of a genus (*Rhizophora*, especially *R. mangle* of the family Rhizophoraceae) of tropical maritime trees or shrubs that send out many prop roots and form dense masses important in coastal land building and as foundations of unique ecosystems’ or ‘any of numerous trees (of the genera *Avicennia*) with growth habits like those of the true mangroves’ [19]. The Cambridge dictionary defined mangroves as tropical trees growing near water developing twisted roots growing partly above the ground [20].

On the other hand, Spalding [14] defined mangroves as ‘trees or large shrubs including ferns and palms growing in or adjacent to intertidal regions which can easily adapt themselves in their environment’. However, these definitions sound paradoxical since many other plants can be mistaken for mangroves. For example, *Anemopsis california* (lizard tail) is a herb growing in wet or shallow waters [21], *Atriplex* (saltbush, genus of 250–300 species) is defined as a shrub growing in salty soils, and *Limonium* (sea lavender, genus of 120 species) is defined as a woody shrub growing along the coasts and in salt marshes [22]. These named plants are small in size and grow in saline conditions similarly to mangroves. Accordingly, it can be pointed out that mangroves do not have an appropriate and precise definition that demarcates the plants from any other halophytes.

## 4. Botanical Classification and Types of Mangroves

**Figure 1:**
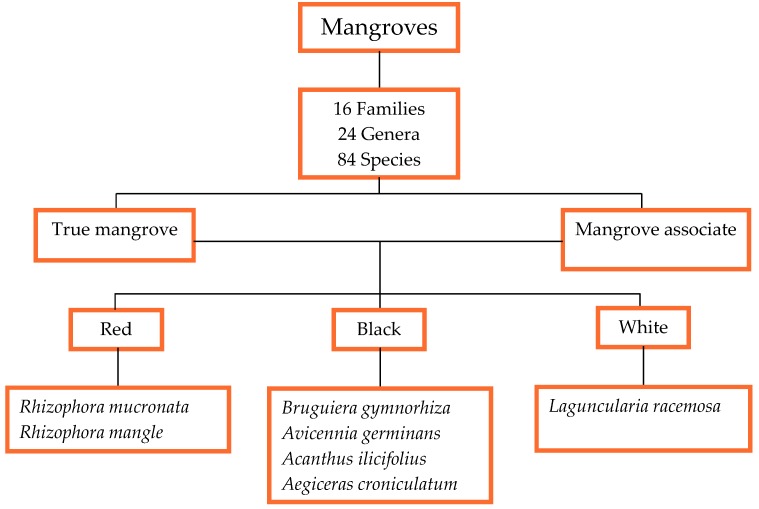
Classification of mangroves.

All mangroves belong to the Malpighiales order consisting of 16 families, with Rhizophoraceae being the dominant family, 24 genera and 84 species in all (Figure 1). Generally, mangroves are classified as true mangroves and mangrove associates. However, the classification does not meet the consensus of all scientists and therefore remains a debatable issue. For instance, *Heritiera littoralis* Aiton is classified as a true mangrove by many researchers [23,24,25,26] but is recognized as a mangrove associate by Mu et al. [27] and Mukherjee et al. [28], and Tansley and Fritsch [29] mentioned that the difference between the two groups might be based on the physiological adaptation to the environment, but this hypothesis still needs to be tested.

The Rhizophoraceae family comes from the major division of Angiosperms (flowering plants) and the well-known Malpighiales order. It is estimated that there are 350, 699 flowering plants or Angiosperms with 405 families, 14,559 genera, and 951,140 species [30]. As quoted by The Plant List (2013), the Rhizophoraceae family has 18 plant genera and 142 accepted species. However, not all species from the Rhizophoraceae family are considered as true mangroves; only 24 species from the four genera *Bruguiera*, *Ceriops*, *Kandelia*, and *Rhizophora* are called the ‘Mangrove trees’ [31]. Rhizophoraceae family is known as the richest family concerning mangrove species. However, although Rhizophoraceae family encompasses many mangrove species with aerial roots, all should not be taken as the ‘Mangrove family’ [32].

According to Bandaranayake [33], plant species that are considered to be true mangroves could also originate from at least 17 different families. Indeed, it is reported that a total of 69 species in 27 genera belonging to 20 families are considered as true mangroves [6,34,35]. On the other hand, Nebula et al. [31] updated the total number of species from 69 to 84, from 27 genera to 24, and from 20 families to 16. Recently, Thatoi et al. [7] opined that among the 84 mangroves species, only 70 of them are true mangroves and the remaining 14 are mangrove associates. Therefore, it can be understood that mangrove trees do not necessarily come from the Rhizophoraceae family but can also come from other families such as Acanthaceae, Avicenniaceae, and Meliaceae, among others.

Mabberley (2008) stated that the principal genera in Rhizophoraceae are *Bruguiera, Carallia, Ceriops, Crossostylis, Pellacalyx*, and *Rhizophora*. Based on the molecular phylogenetic and floral structures analyses, it is clear that Rhizophoraceae has a sister group which is the Erythroxylaceae, from which cocaine is derived [26]. Principally, this review is based on different mangrove species of therapeutic values such as *Avicennia marina* (Forssk.) Vierh, *Avicennia officinalis* L., *Avicennia ilicifolius* L., *B. gymnorhiza* (L.) Lam, *Excoecaria agallocha* L., *Heritiera fomes* Buch.-Ham., *Kandelia candel* (L.) Druce, *R. mucronata* Lam, and *Xylocarpus granatum* J. Koenig among others. In Mauritius, there are only two types of mangrove species that exist, namely *B. gymnorhiza* and *R. mucronata*. Both species originate from the Rhizophoraceae family [31]. Mangroves are spread in 16 families, 24 genera, and 84 species with three main types, namely red, black, and white (Figure 1).

Seven types of mangrove trees exist, among which three are most dominant namely the red, black, and white mangroves. In Mauritius, two dominant types of mangrove are grown along the coastlines, namely the red (*R. mucronata*) and the black (*B. gymnorhiza*) types. Besides these two species, there is another mangrove species namely the *Cassipourea gummiflua* var. verticillata (N. E. Br.) J. Lewis which is scarcely cultivated in the Sir Seewoosagur Ramgoolam (SSR) Botanical Garden, Pamplemousses, Mauritius. The difference between the three most common types of mangrove (red, black, and white mangroves) is distinctive from each other based on their leaves, roots, and fruits (propagules) (http://www.mangrovesgy.org/home/index.php/2014-04-27-16-39-08/types-of-mangroves). Table 2 describes the distinguishing characteristics between the three dominant mangrove types. However, most works of literatures have not specified or classified the mangrove species on which studies were conducted concerning their types which consequently results in only a few examples given in Table 2 and Figure 1.

Additionally, the difference is based on the tide level they survive. For instance, red mangroves grow in the low tide, black mangroves are mostly found growing in medium high tide, while white mangroves grow on a higher tide level experiencing lower tide flushing compared to the other two types (https://wetlandsandwildlife.wordpress.com/2017/03/06/featured-content-2/). Furthermore, there are other types of mangroves known as the Buttonwood, but they are not considered as true mangroves since they produce seeds instead of propagules and grow on a higher upland area compared to white mangroves (https://wetlandsandwildlife.wordpress.com/2017/03/06/featured-content-2/).

## 5. Biogeographical Distribution of Mangroves

Mangrove forests are known as the world’s most productive ecosystems, and they occur mainly in the tropical or sub-tropical regions [36]. Mangroves are found in 123 countries across the globe [14]. The total area covered by mangrove trees in the world was estimated to be 137,760 km^2^ in 2000 [37] and currently mangroves covered about 152,000 km^2^ [9]. Approximately 75% of mangroves are found in 15 countries with only 6.9% of them are protected [38]. The top 20 mangrove nations in the world with Indonesia covering the largest area followed by Brazil, Malaysia, and lastly Cameroon [8] are shown in Table 3.

Overall, Asia consists of the largest amount of mangrove’s forest (42%) in the world followed by Africa (21%), North/Central America (15%), and lastly by South America (11%).

**Figure 2:**
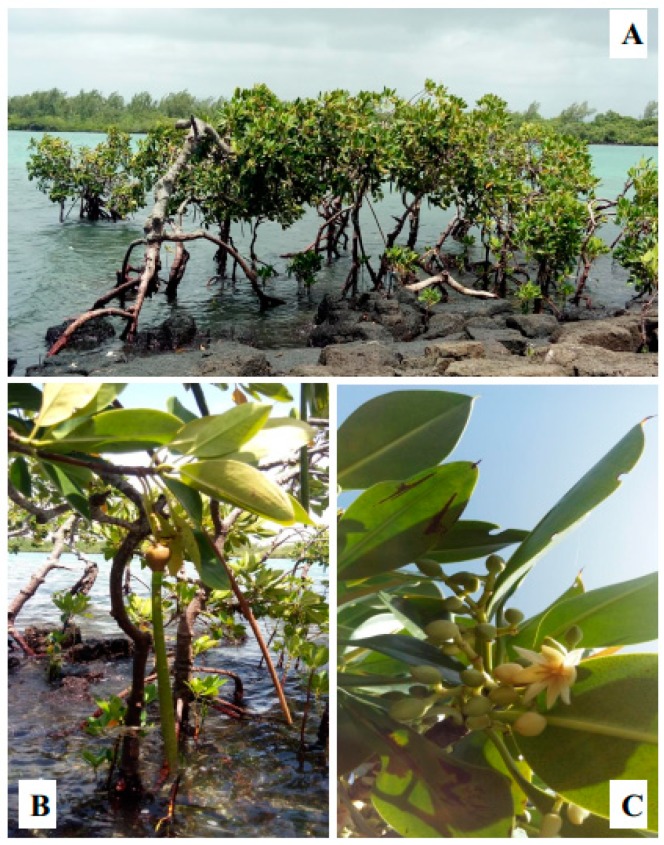
(**A**) *R. mucronata* growing along the coastline at Bras d’Eau public beach, Mauritius; (**B**) propagule; (**C**) flower.

**Figure 3:**
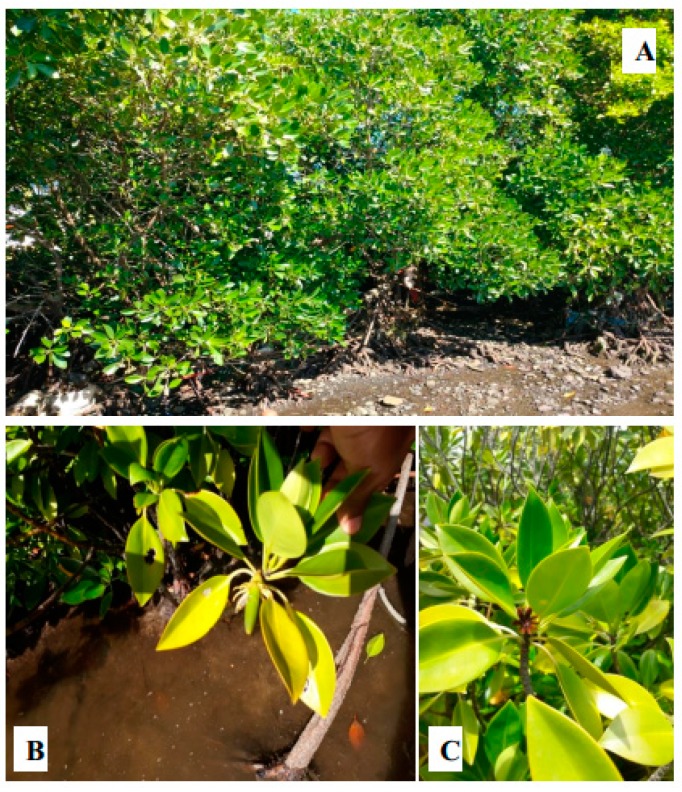
(**A**) *B. gymnorhiza* growing along the coastline at Bambous Virieux, Mauritius; (**B**) propagule; (**C**) flower.

In Mauritius, *R. mucronata* occupies an approximate area of 20 km^2^ of the coastline of Mauritius and are found mostly on the northeast, east, and southeast coastline of the island (Grand Gaube, Pointe des lascars, Poste la Fayette, Ile aux Cerfs, Trou D’eau Douce, Beau Champ, Grand Sable, Mahébourg) and is found scarcely in the south-southwest coasts (Maconde, Tamarin) [39]. There are only two predominant species of mangroves on the island namely, *R. mucronata* (Figure 2) and *B. gymnorhiza* (Figure 3) [40].

Mauritius has lost 30% of its mangrove population in seven years (1987–1994) from 20 km^2^ to 14 km^2^. Mangroves were abundantly used for firewood, construction purposes, and cut to provide a pathway for boats. In the mid-1990’s, a restoration program was set up and is still active. As a result, over the past 15 years, 23 hectares of mangrove trees were restored along with approximately 230,000 seedlings [41].

## 6. Morphological Characteristics

The distinct morphological characteristic of mangrove plant is linked with its root systems. All mangroves have special roots known as rhizophores (buttress, stilt, or arc-shaped prop roots) or pneumatophores (pencil-like roots). These types of roots act as a respiratory system for the plants to facilitate gas exchange since mangroves grow in high saline conditions and anaerobic soils. Mangroves are called halophytes since they have good salt tolerance and filter sea water effectively for their usage. The height of the plants varies from 2 m to 50 m. Their leaves are thick, elliptical in shape, and dark green in color, except for *Nypa fruticans* Wurmb (commonly known as Nypa palm) species which have thin long leaves resembling leaves of a palm tree. The fruits of most mangrove species have a cigar-shaped structure (long and cylindrical), green in color, and varying in length ranging from 2 cm to 25 cm. Table 4 summarizes the morphological characteristics of various mangrove species. 

## 7. Ethnopharmacological Uses

**Figure 4:**
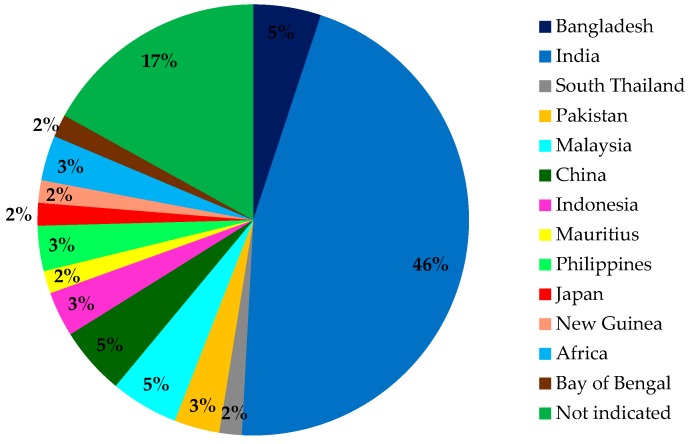
Countries using mangroves traditionally.

Mangroves have shown potential and promising therapeutic applications to treat a variety of ailments as reported by many ethnomedicinal studies. Various parts of the plants such as the leaves, roots, barks, or stems have been used in folk medicines. They are mainly used medicinally to treat diabetes, hypertension, and gastrointestinal disorders such as constipation, diarrhea, dysentery, dyspepsia, hematuria, and stomach pain. The plants are mostly used in Asian countries, namely India (45.8%), Bangladesh (5.1%), Malaysia (5.1%), China (5.1%), Indonesia (3.4%), Philippines (3.4%), and other countries with 16.9%. (Figure 4). No report is available for the traditional usage of mangroves in European countries.

**Figure 5:**
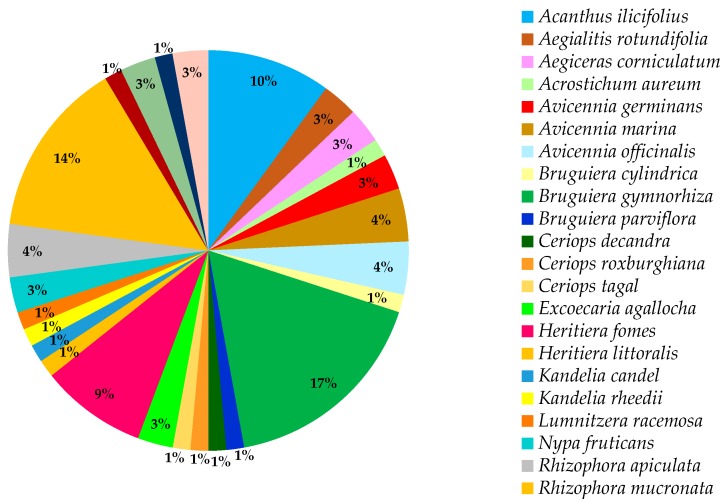
Traditionally used mangrove species.

Species such as *B. gymnorhiza* (17%), *R. mucronata* (14%), *A. ilicifolius* (10%), and *H. fomes* (9%) are widely used traditionally and possess an array of potential medicinal values compared to the other species (Table 5 and Figure 5). For instance, *A. ilicifolius* is used to treat asthma, diabetes, hepatitis, leprosy, rheumatism, snake bites, among others. In India, the fruits are crushed and used as a dressing for snake bites. Additionally, the whole plant can be boiled in water, and the resulting decoction can be consumed to remove kidney stones [58]. In India, the bark decoction of *X. granatum*, although poorly exploited (1.85%), is used for treating cholera and diarrhea [58].

*B. gymnorhiza* (Rhizophoraceae) is widely distributed in the Indian Ocean through Malaysia and Australia. The leaves and roots are mostly used in Bangladesh, China, India, and Indonesia to treat angina, diarrhea, eye disease, fever, hypertension, and intestinal worms, among others. In Comoros and Mauritius Islands, a decoction prepared from the root (15 cm length) of *B. gymnorhiza* and five to seven leaves of *Piper borbonense* boiled in two cups of water, is taken twice in a day to treat haemorrhage [13]. The same decoction is also used for diabetes and hypertension.

*R. mucronata* (Rhizophoraceae) is commonly found in East Africa, Australia, and the Indian Ocean. *R. mucronata* is widely used in India. This mangrove species has tannins up to 70% of tannins which is responsible for the medicinal properties including astringent, anti-diabetic, anti-rheumatism, and hypotensive [13]. The plant is most traditionally used against diarrhea, constipation, nausea, hematuria, and diabetes. In New Guinea, it is used to cure fertility and menstruation disorders [59]. Interestingly, in Indonesia, the whole plant is used to treat elephantiasis, which is a condition caused by the enlargement of tissues due to filarial worms [60,61]. Both *Bruguiera* and *Rhizophora* genera are known to be useful for treating a wide array of diseases such as angina, haemorrhage, hematuria, and interestingly, mature leaves and roots can be used for childbirth [62], ulcers [63], diarrhea, fever, burns [64], and stings of poisonous fish [13]. Table 5 summarizes and gives a greater insight into the traditional uses of different mangrove species in different countries.

## 8. Pharmacological Activities

The importance of mangroves in the medical field for curing diseases cannot be undermined as the plants have much therapeutic potential. Mangroves were used in folklore medicines a long time ago, and different extracts from various parts of the plants (roots, leaves, fruits, bark, and resin) have shown exciting and significant inhibitory activities in many assays namely antidiabetic, anti-inflammatory, anti-cancer, anti-ulcer, anti-tumor, anti-viral, antioxidant, and antimicrobial among others. Since various parts of the plants were used for inhibitory assays and considering the fact that mangrove ecosystems are known to be threatened, it can be said that plant samples were being used sustainably. Although many mangrove species have been used traditionally by local inhabitants for an extended period following folk traditions in various countries as ailments, many among them have not been studied extensively yet, and thus their medicinal properties have not been reported. For example, in Mauritius, local people use the root decoction of *R. mucronata* against diabetes, but the plant has not been locally validated by researchers to confirm its pharmacological properties. Similarly, no scientific research has been carried out so far on *Ceriops tagal* and *Kandelia rheedii* to prove their efficacy against diseases that can be cured by folk medicine. Interestingly, although few studies have been conducted on the species *Bruguiera sexangula, Rhizophora stylosa,* and *Pelliciera rhizophorae*, these species are yet to be used in folk medicine (Table 6). Therefore, mangrove species require more attention from researchers to shed more light into the traditional and pharmacological uses of these unique plants as there is a dearth of knowledge on this particular area. Table 6 shows the number of species used in folklore medicines and those that are pharmacologically tested.

**Figure 6:**
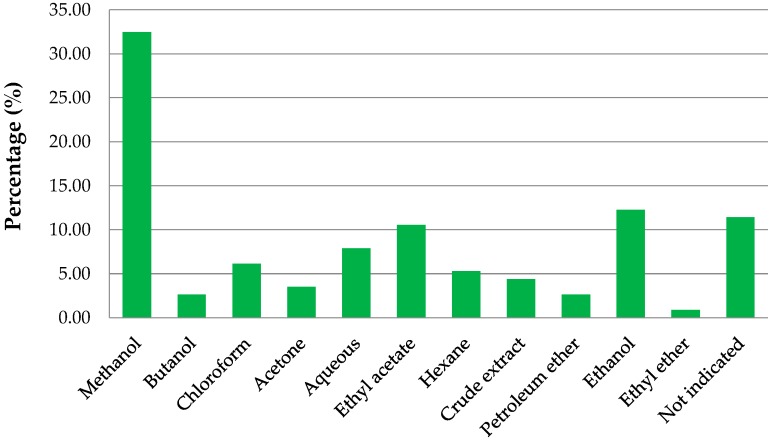
Types of mangrove extracts used in inhibition assays.

It has been acknowledged that out of the 84 mangrove species that exist, only 26 species were mentioned in literature to possess folklore medicinal importance. However, it could be possible that the remaining 58 species have an equally influential role in the management of diseases, but due to a lack of interest, there is a dearth of knowledge of all the mangrove species. Table 7 represents the pharmacological activities of various mangrove species studied and gives a broader knowledge on the pharmacological importance on mangroves and on the different types of assays conducted. Figure 6 illustrates the types of extracts commonly used for these assays.

Methanolic extracts (32.46%) were the most preferred extracts used in most studies followed by ethanolic (12.28%), ethyl acetate (10.53%), aqueous (7.89%), and chloroform (6.14%) (Figure 6). The percentage was calculated as per report per species mentioned in Table 7.

**Figure 7:**
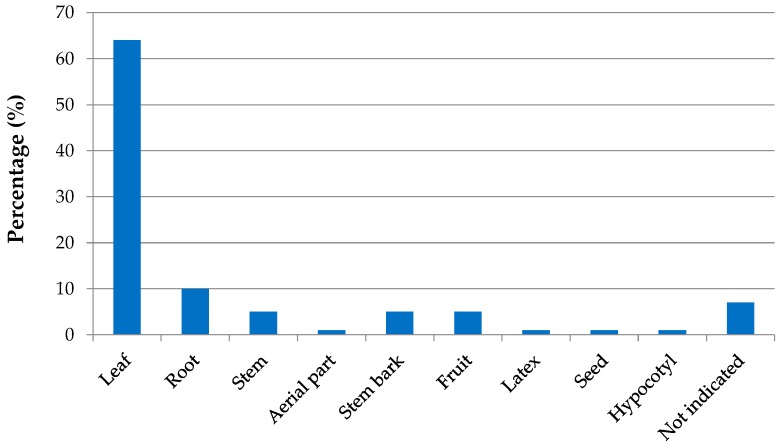
Types of plant parts of mangroves used in inhibition assays.

On the other hand, Figure 7 illustrates the types of plant parts most commonly used in the studies mentioned in Table 6. From the data shown, it can be suggested that the plant parts mostly studied are leaves (64%), roots (10%), stem bark (5%), and stem (5%). Only one work, published by Mondal et al. (2016), used latex and seed as plant samples to carry out anti-inflammatory, anticancer, analgesic, and anti-filarial activities and Wei et al. [93] conducted a test on the hypocotyl part to determine antioxidant property.

**Figure 8:**
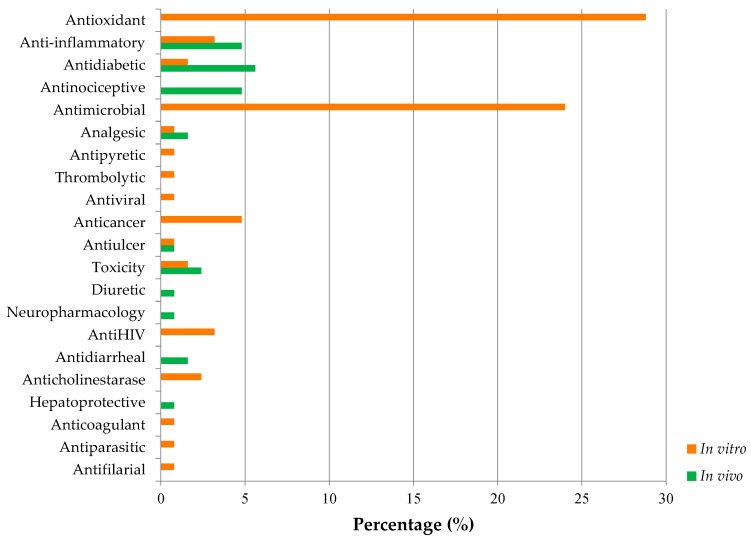
Types of assays.

Figure 8 illustrates the types of assays usually conducted on mangroves. It is evident that antioxidant (28.8%) and antimicrobial (24.0%) assays were the two most common in vitro studies performed. Interestingly, most in vivo studies were done for antidiabetic assays compared to in vitro. It is found that antipyretic, antiviral, thrombolytic activity, anticoagulant, antiparasitic, antiulcer, and anti-filarial tests were less seldom conducted. However, it is important to highlight that many mangrove species are used as a remedy for the ulcer in folklore medicine. For instance, the leaf of *A. marina*, the leaf of *A. officinalis*, the bark of *B. cylindrica*, bark, fruit, and leaf of *C. decandra*, whole plant of *C. roxburghiana,* and whole plant of *R. mucronata* (Table 5) are traditionally believed to cure ulcers. Nonetheless, the antiulcer potential of these named plants has not been extensively validated either in vivo or in vitro studies to confirm this belief in medical lore.

**Figure 9:**
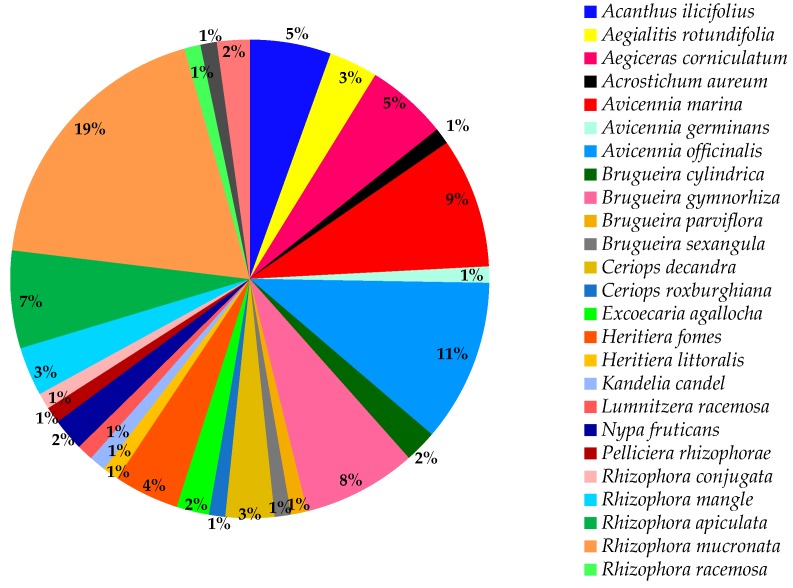
Pharmacologically validated species of mangroves.

A pie chart in Figure 9 represents mangroves that have been pharmacologically validated. The five most reportedly investigated species are *R. mucronata* (19%), *A. officinalis* (11%), *A. marina* (9%), *B. gymnorhiza* (8%), and *R. apiculata* (7%). It is important to highlight that *B. gymnorhiza* is the most traditionally used species (Figure 9), but it is found in the fourth place to be pharmacologically validated. This warrants an in-depth study on that particular species since its importance in folklore medicine.

## 9. Phytochemistry of Mangroves

**Figure 10:**
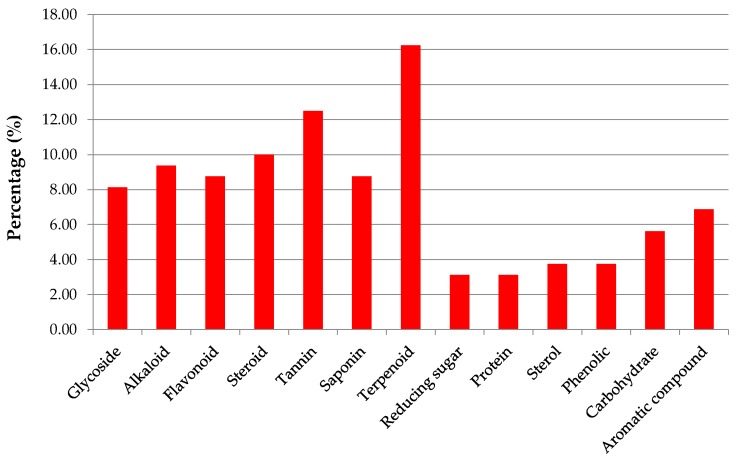
Classes of compounds isolated from mangroves.

Plants possess a plethora of novel and biologically active secondary metabolites and thus serves a reservoir for the production of novel drug compounds. In this era in which most researchers are screening thousands of plants for the discovery of novel compounds, it is thus of high importance to scrutinize mangrove species with that very aim to isolate new phytochemicals which can be potential candidates for the development of pharmaceutical drugs. Saying so, about 200 bioactive metabolites have already been identified from mangroves [36,148]. Therefore, this prompts more studies for phytochemical screening of new metabolites. Phytochemical studies conducted on various mangrove species are summarized in Table 8. Histogram in Figure 10 illustrates the 16 most common types of phytochemicals isolated from mangrove species. Generally, the seven most common chemical constituents present are terpenoids (16.25%), tannins (12.5%), steroids (10.0%), alkaloids (9.38%), flavonoids (8.75%), saponins (8.75%), and glycosides (8.13%). Furthermore, mangroves also yielded other compounds namely fatty acid derivative, anthraquinone, amino acid, coumarin, quinine, ester, gum, phenol, terpene quercetin, and anthranoid. However, these compounds are found at low levels and are present in only certain mangrove plants. For example, the presence of fatty acids has been reported only in *A. ilicifolius* and *A. marina* but not in any other species (Table 8).

There is an undeviating link between phytochemicals and pharmacological activities. Kathiresan et al. [149] have shown that the bioactive compounds such as galactose, galactosamine, glucose, and arabinose possess significant anti-HIV activity. The different types of constituents present in medicinal plants are responsible for the wide range of pharmacological activities that the plants possess. It is reported that plants grown along the coastal regions are known to be potential resources of anticancer drugs [149]. For instance, the constituent tannin isolated from the species *B. sexangula* showed anticancer activity against Lewis lung carcinoma and Sarcoma 180 [149,150]. Additionally, the bark extracts of this mangrove species have shown antitumor activity which was due to the tannin-free aqueous residue containing the alkaloid, brugine, tropine, and its acetic ester acid [149]. Compounds produced by the species *R. mangle* also showed potent activity against carcinomas, melanomas, and lymphomas [149].

Barik et al. [121] were the first to isolate a flavone known as 5,7-dihydroxy-2-(3-hydroxy-4, 5-dimethoxy-phenyl)-chromen-4-one-a, from the leaves of *B. gymnorhiza*. It has been reported that the compound was responsible for anti-inflammatory activity with a percentage inhibition of 80% against COX-2 mediated prostaglandin E2 production. Phenol group is a bioactive chemical compound that shows good antioxidant activity. High antioxidant activity was exhibited by the methanolic fruit extract of *B. gymnorhiza* with an IC_50_ value of 13.47 ppm. The fruit is rich in carbohydrate (29.28%) and thus can become a potential food source [64,151]. Sur et al. [64] reported that polyphenols such as gallic acid, quercetin and coumarin isolated from the methanolic leaf extract showed significant antioxidant activities. These constituents help in nursing the injury of hepatic tissue through its antioxidant effects. Moreover, constituents mainly flavonoids, reducing sugars, gums, saponins, and tannins isolated from the roots of *B. gymnorhiza* are responsible for antinociceptive and antidiarrhea properties [152].

**Figure 11:**
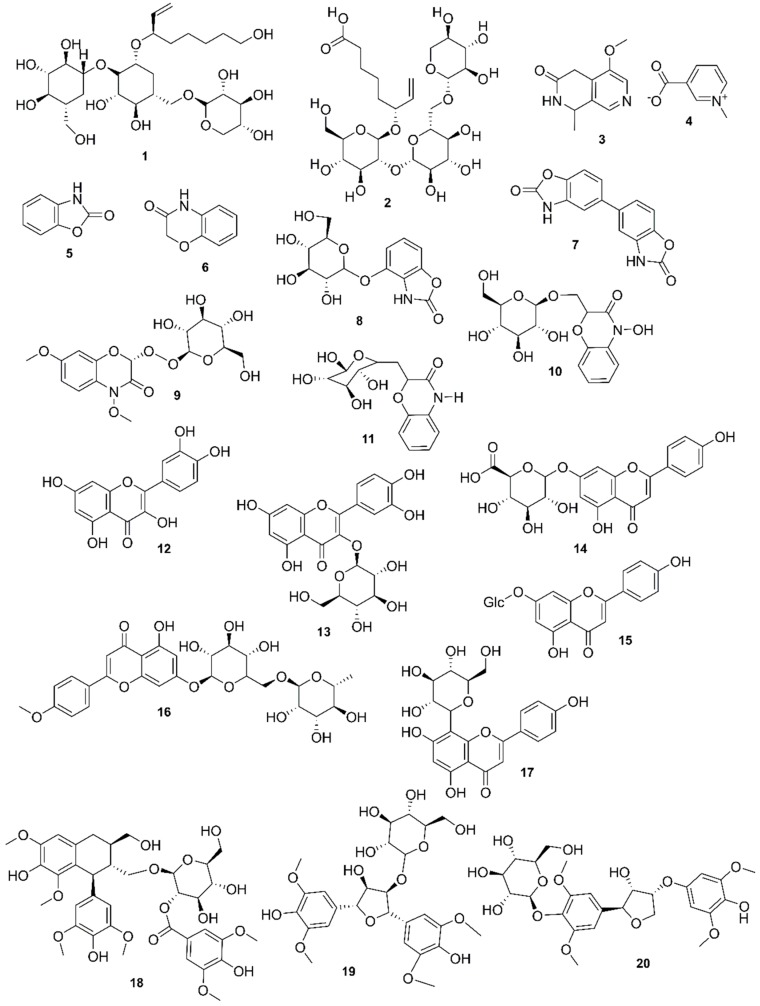
Chemical structures of compounds **1**–**20** isolated from mangrove species.

**Figure 12:**
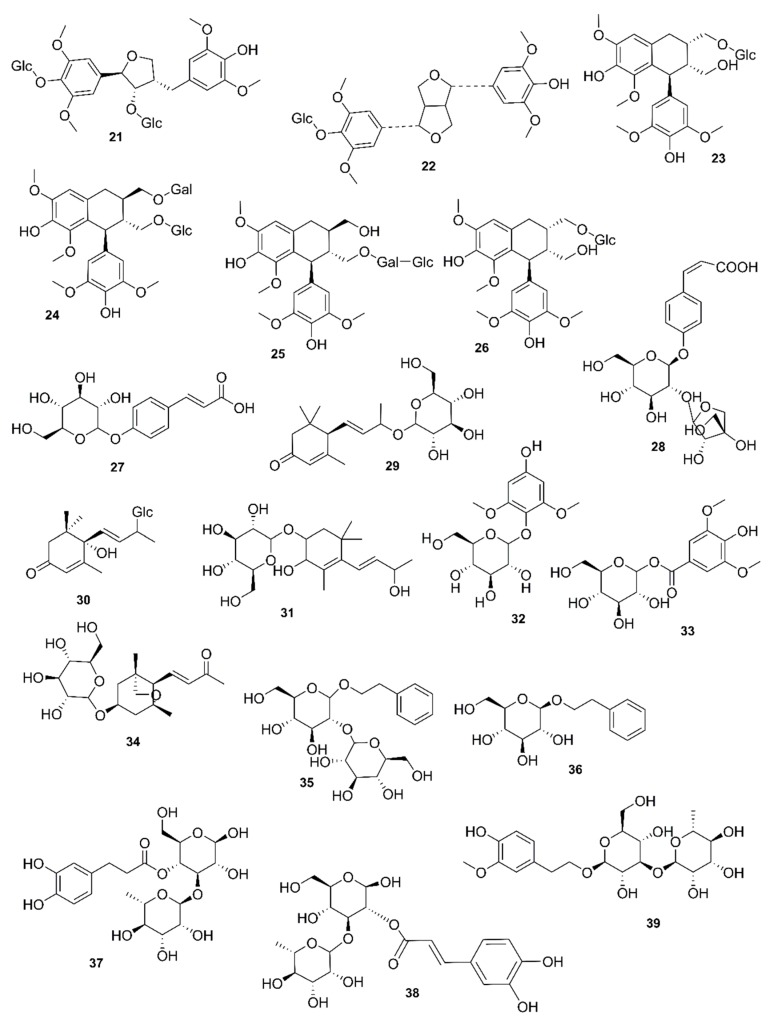
Chemical structures of compounds **21**–**39** isolated from mangrove species.

**Figure 13:**
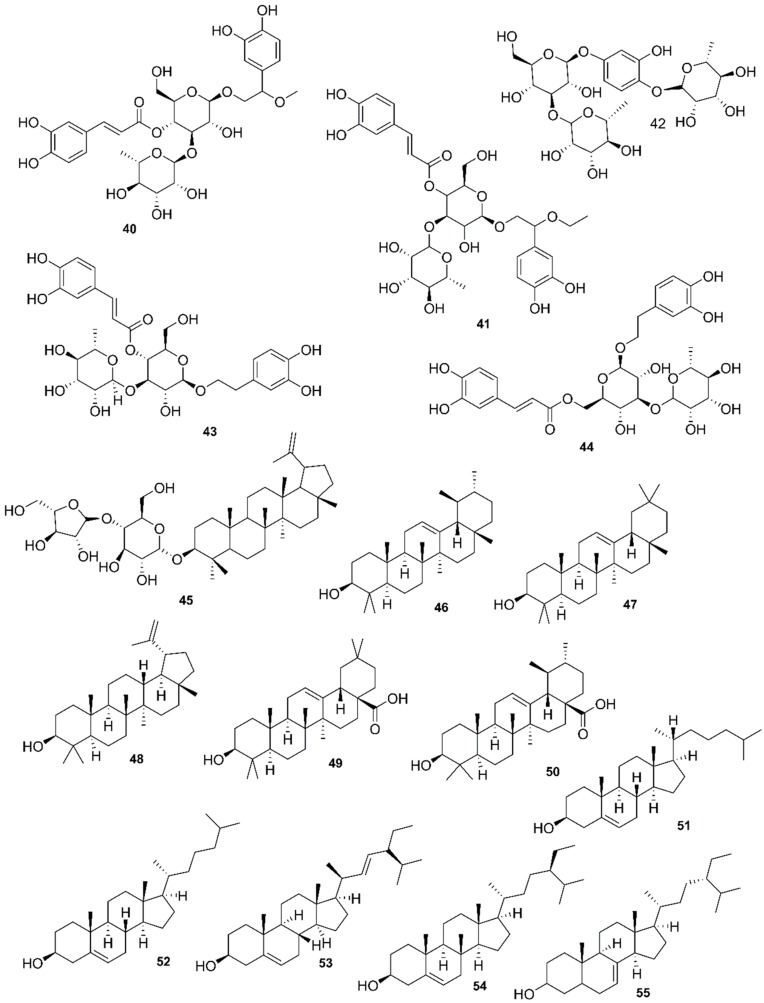
Chemical structures of compounds **40**–**55** isolated from mangrove species.

**Figure 14:**
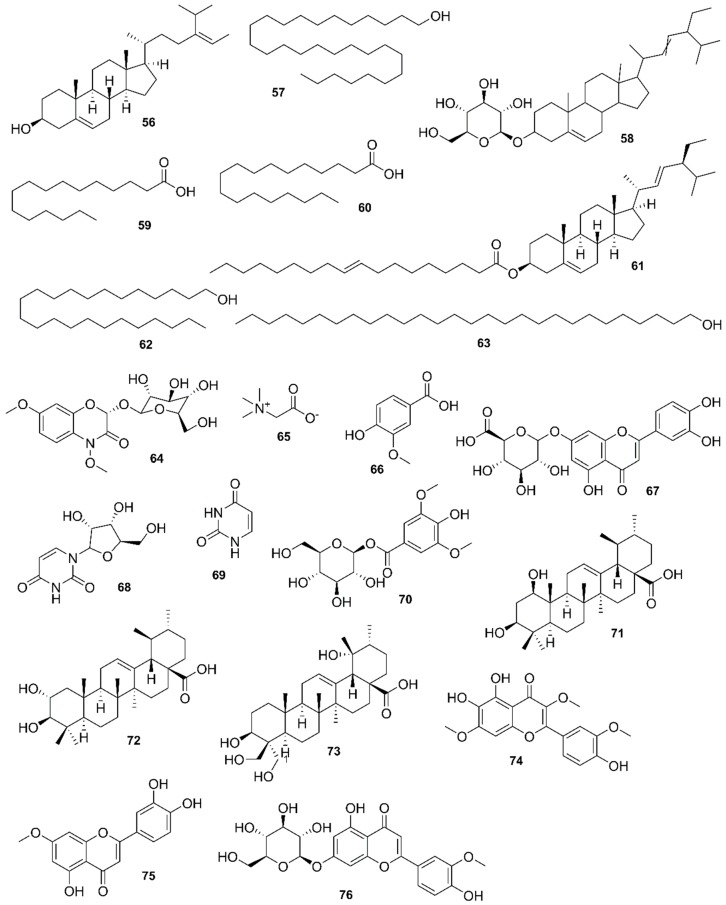
Chemical structures of compounds **55**–**76** isolated from mangrove species.

**Figure 15:**
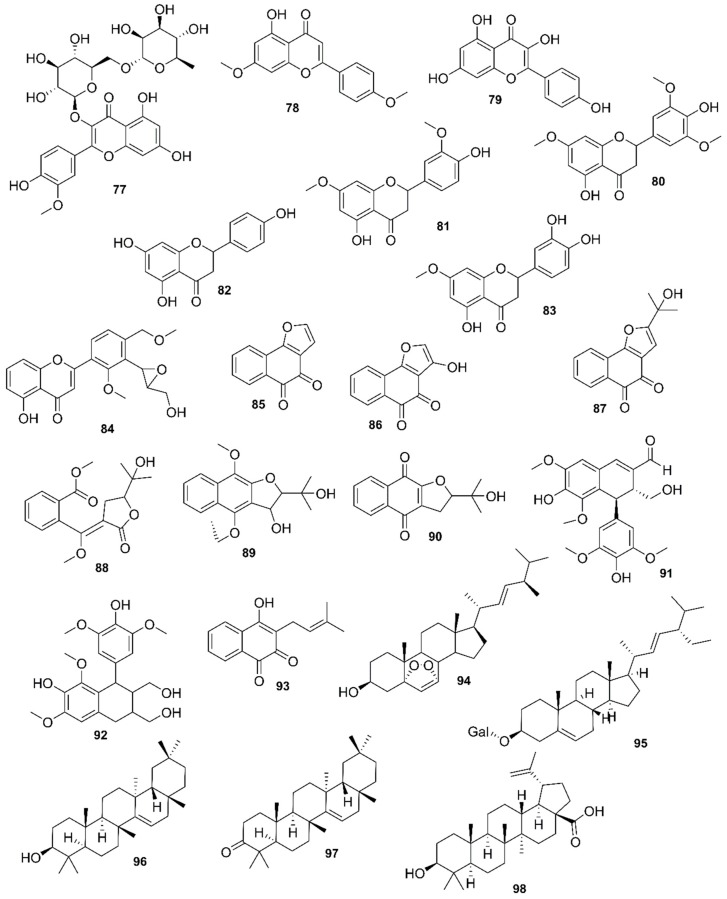
Chemical structures of compounds **77**–**98** isolated from mangrove species.

**Figure 16:**
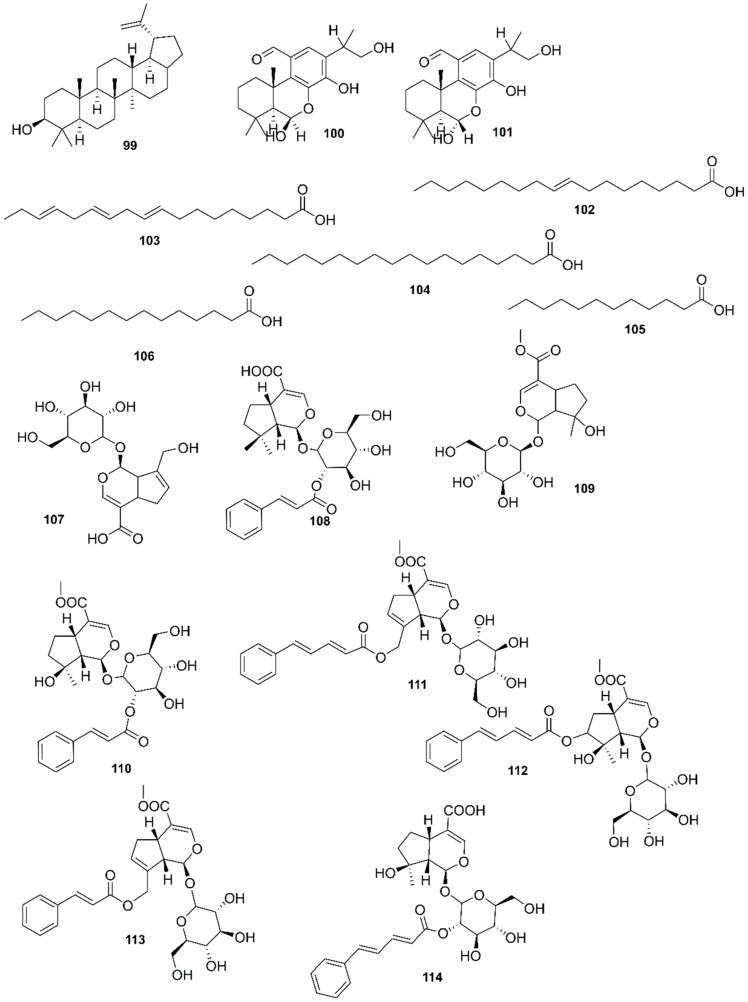
Chemical structures of compounds **99**–**114** isolated from mangrove species.

**Figure 17:**
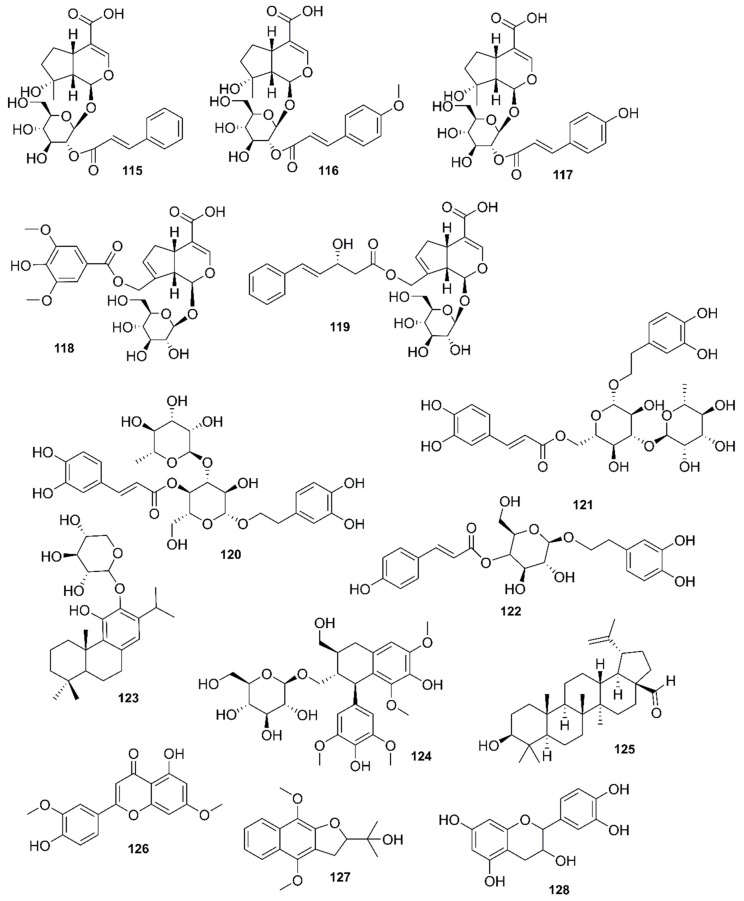
Chemical structures of compounds **115**–**128** isolated from mangrove species.

**Figure 18:**
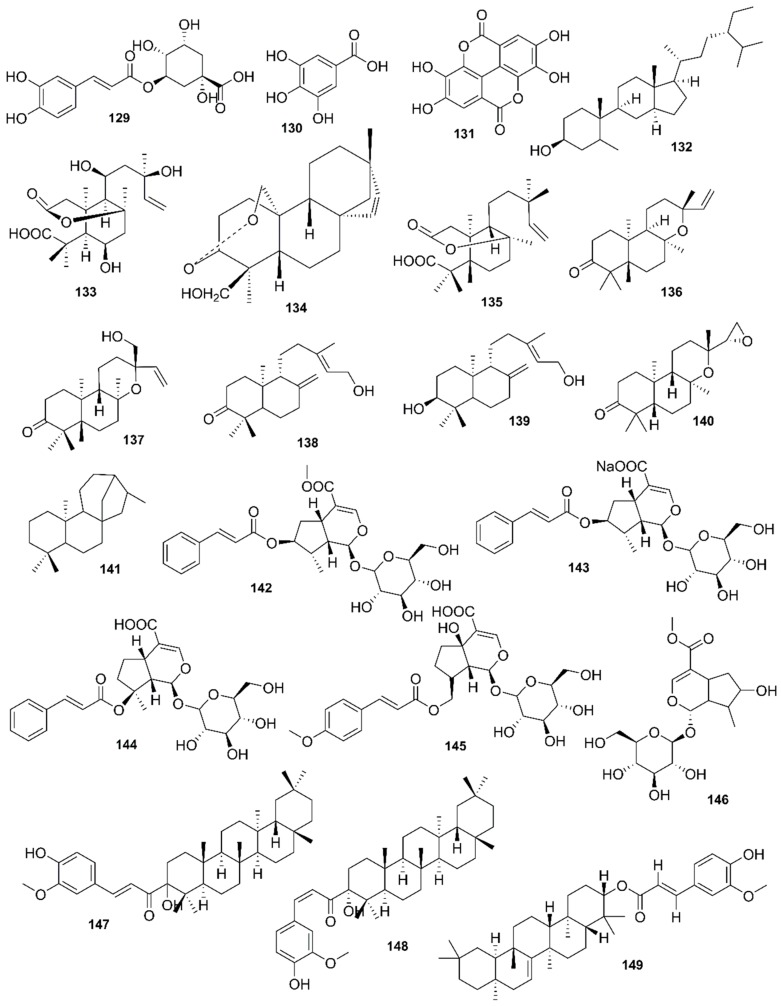
Chemical structures of compounds **129**–**149** isolated from mangrove species.

**Figure 19:**
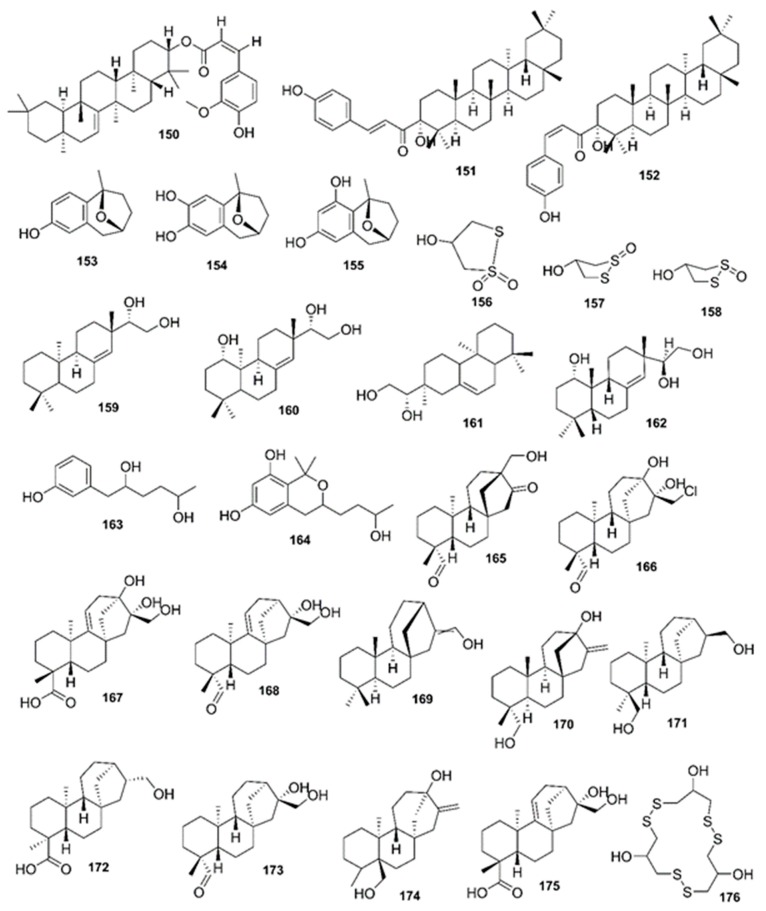
Chemical structures of compounds **150**–**176** isolated from mangrove species.

**Figure 20:**
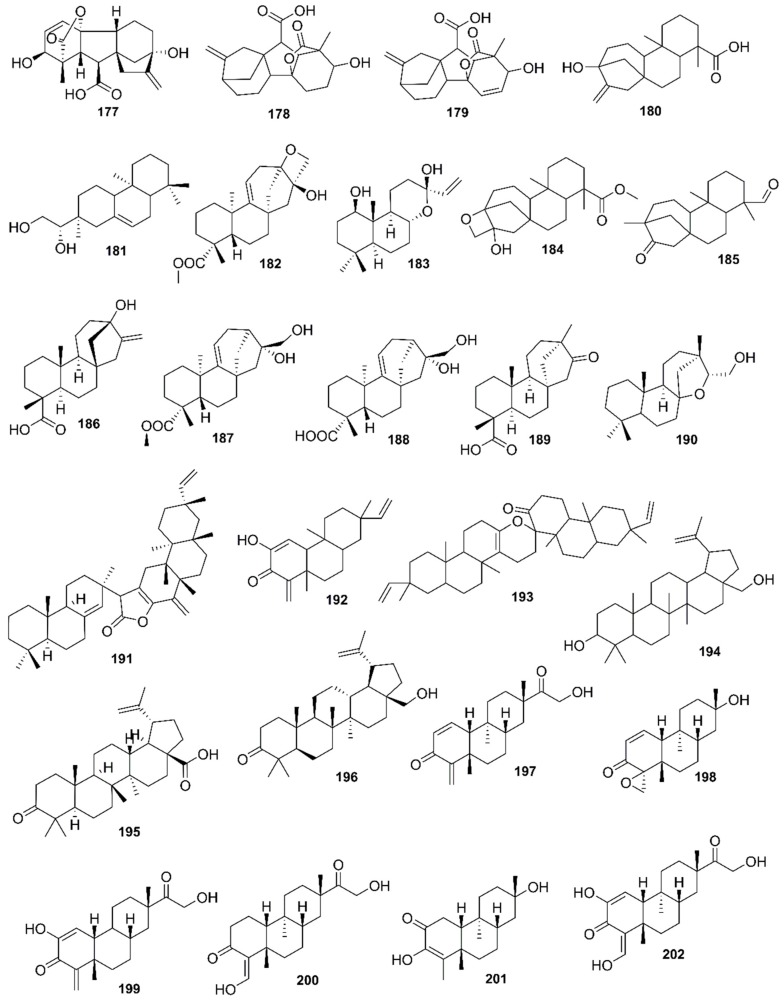
Chemical structures of compounds **177**–**202** isolated from mangrove species.

**Figure 21:**
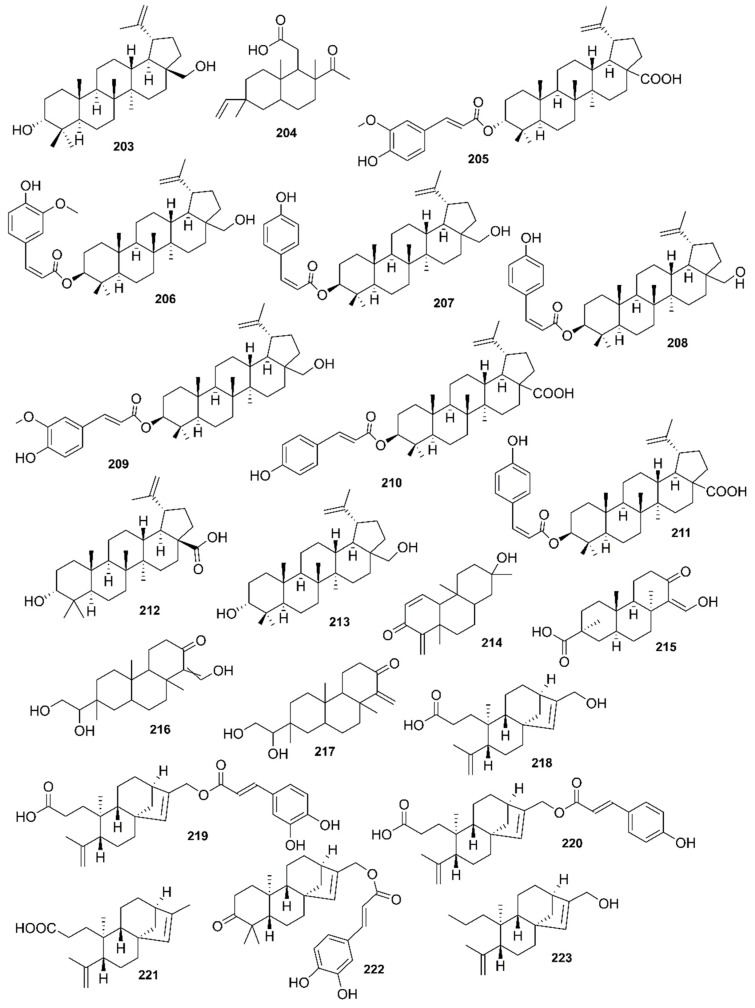
Chemical structures of compounds **203**–**223** isolated from mangrove speices.

**Figure 22:**
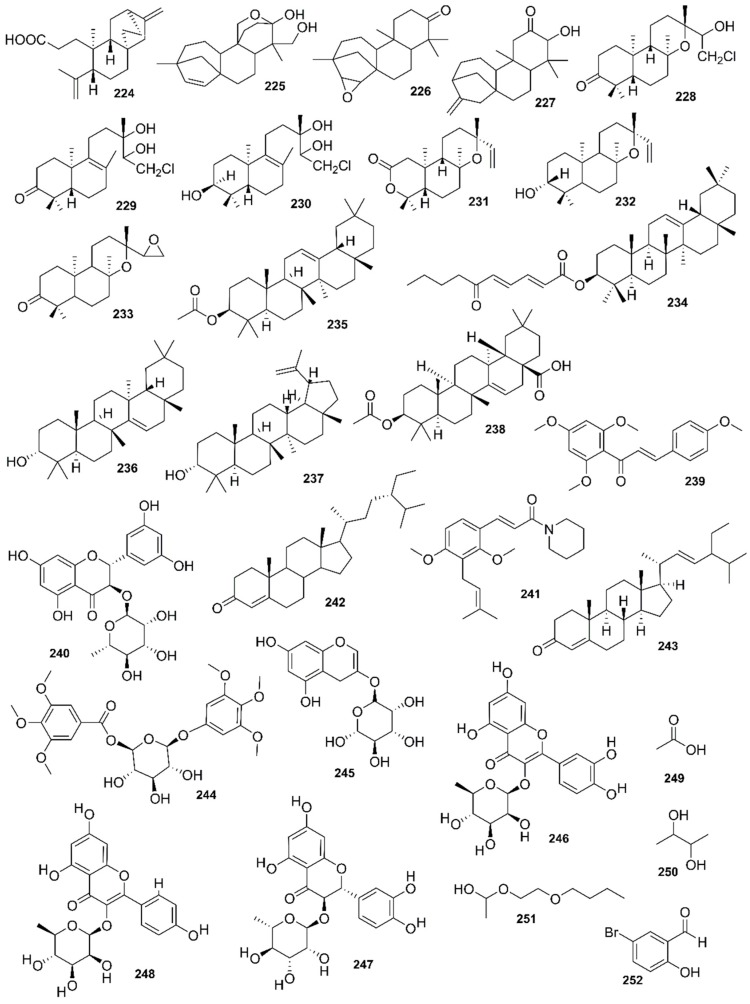
Chemical structures of compounds **224**–**252** isolated from mangrove species.

**Figure 23:**
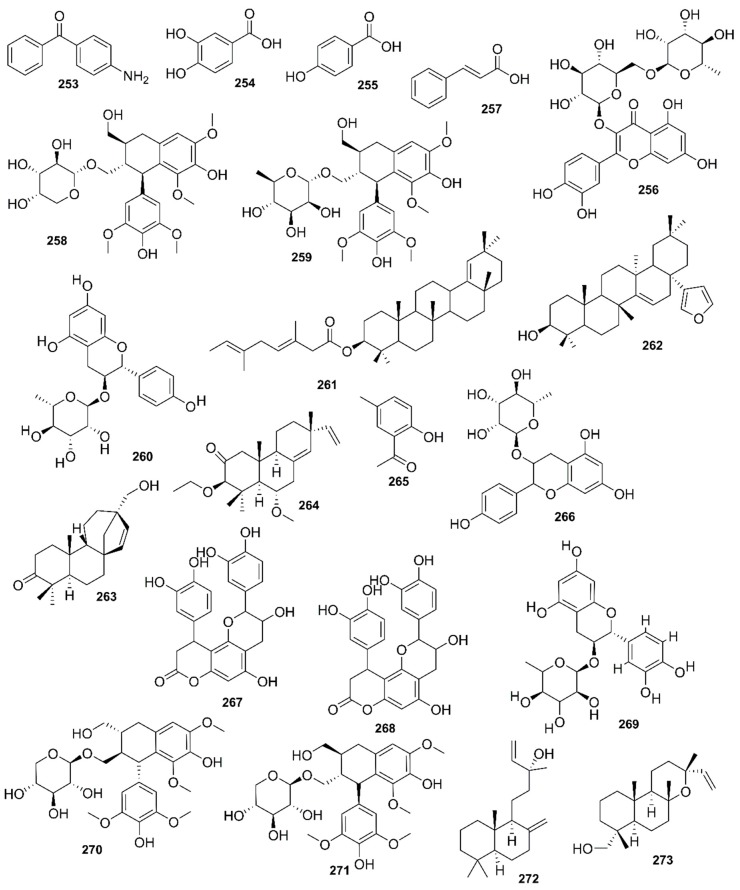
Chemical structures of compounds **253**–**273** isolated from mangrove species.

**Figure 24:**
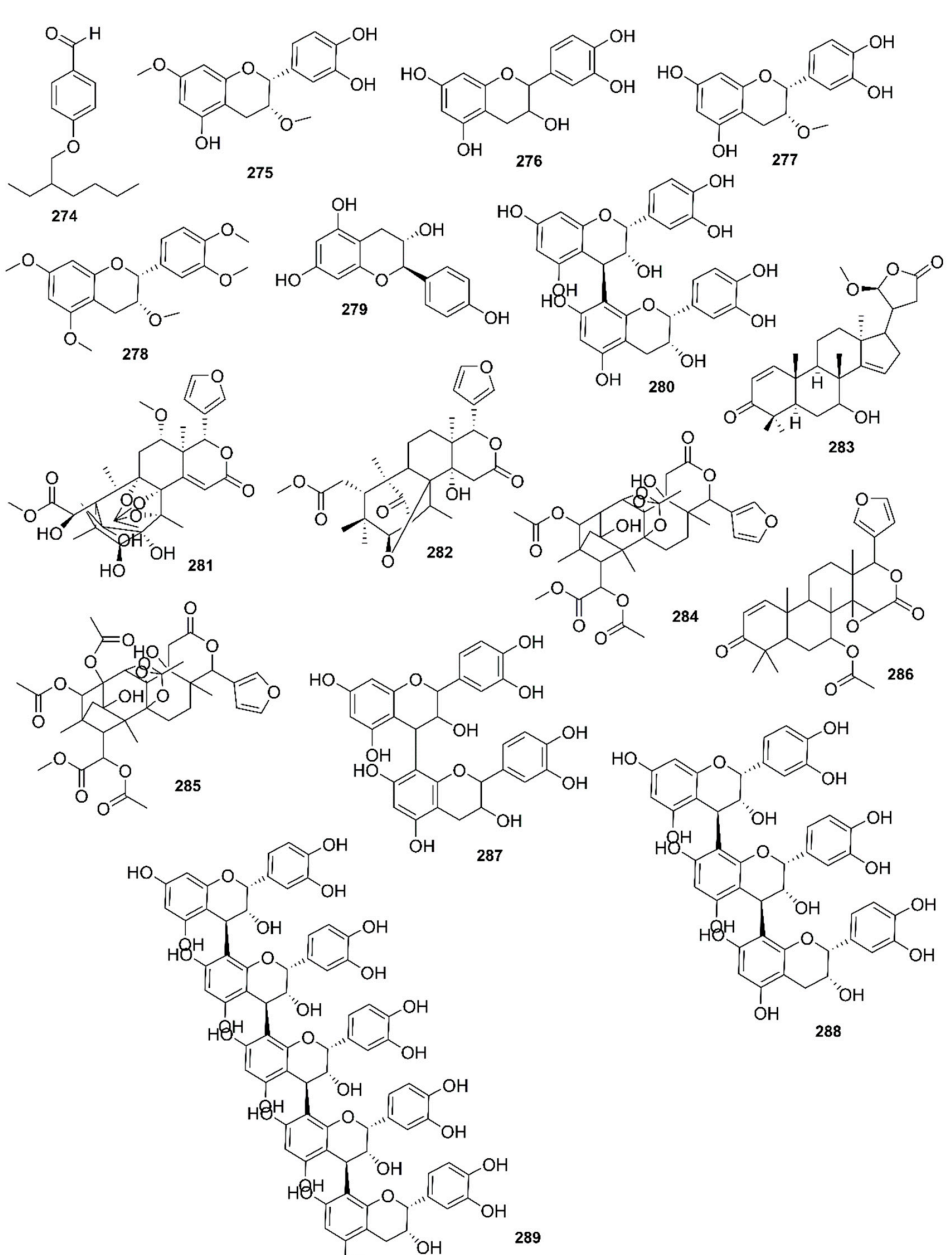
Chemical structures of compounds **274**–**289** isolated from mangrove species.

*Rhizophora mucronata*, another popular mangrove species consists of a broad spectrum of chemical constituents such as sugar, tannins, saponins, alkaloids, flavonoids, steroids, terpenoids, glycosides, phenolics [60,88,153], gibberellins, lipids, inositols, anthocyanidins, polysaccharides, proteins, minerals, hydrolysable tannins, and polyphenols (Balasubramanian et al. 2015). From the phytochemical screening test of *R. mucronata* the alkaloid, rhizophorine is considered as a major component in the leaf of the plant [65]. The methanolic leaf extract exhibited a strong anti-cholinesterase activity (AChE assay) with an IC_50_ value of 59.31 ± 0.35 µg/mL and potent antioxidant activity (DPPH) with an IC_50_ value of 47.39 ± 0.43 µg/mL. These significant results could be attributed to the presence of a high number of flavonoids, particularly catechin (**128**) [100]. With regards to antidiabetic activity, *R. mucronata* is considered as an excellent natural antidiabetic agent due to the presence of phenolics, flavonoids, gallic acid (**130**), quercetin (**12**), and coumarin [64]. Furthermore, a study by Rohini and Das [154] revealed the excellent anti-inflammatory activity of the bark extract of *R. mucronata* with the presence of the phytoconstituents lupeol (**48**), quercetin (**12**), β-sitosterol (**54**), and caffeic acid. Manilal et al. [143] are of the view that the main constituent, ethanone (1-(2-hydroxy-5-methylphenyl), isolated from the crude extract could play a pivotal role in the antibiotic activity of the plant. The chemical structures of isolated compounds from mangroves are illustrated in Figure 11, Figure 12, Figure 13, Figure 14, Figure 15, Figure 16, Figure 17, Figure 18, Figure 19, Figure 20, Figure 21, Figure 22, Figure 23 and Figure 24.

## 10. Results and Discussion

### 10.1. Acanthus ilicifolius

*A. ilicifolius* is a small tree of height up to 2 m with stilt roots, sharp edges leaves, kidney-shaped fruits, and large light-violet petals [42]. *A. ilicifolius* is found in Bangladesh, India, and South Thailand [33,65,68]. It is widely used as a traditional medicine by the local people in these countries. In Bangladesh and India, the whole plant parts are used for treating rheumatism, hepatitis, leprosy, skin allergies, snake bites, diabetes, asthma, kidney stones, smallpox, and ulcer (Table 5). In South Thailand, this species is used to treat psoriasis [70].

*A. ilicifolius* is found to have many pharmacological activities; namely, the methanolic leaf extracts have good antioxidant, anti-inflammatory activities [66,105]. Acetone leaf extracts showed good antimicrobial activities. A wide array of phytochemical constituents is found to be present in different parts of the plants namely the leaves, barks, roots, and fruits. Results from GC/MS confirmed the presence of alkaloids (acanthicifoline (**3**), benzoxazin-3-one (**6**)), flavonoids, steroids (cholesterol (**51**), β-sitosterol (**54**)), glycosides, saponins, tannins, and terpenoids [42,155,156] (Table 8). Ribose derivative isolated from this mangrove species known as 2-benzoxazoline exhibited antiviral and antitumor activities [150]. Recently, a new sugar ester was derived from the roots of *A. ilicifolius* known as 1,2-di-(syringoyl)-β-d-glucopyranose [157]. The structure of the new compound was clarified by extensive spectroscopic methods such as NMR and HRESI-MS.

### 10.2. Aegialitis rotundifolia

*A. rotundifolia* is a small tree originating from the Plumbaginaceae family with a height of 2–3 m. It has broad and ovate leaves, 5–8.8 cm long and 4.5–8.5 cm wide [44,45]. The species is mainly grown in Bangladesh and is used to cure inflammatory and painful arthritis [72,73]. The leaf infusion is used as an anti-ache agent. *A. rotundifolia* showed moderate inflammatory and anti-pyretic activities with aqueous leaf extracts [73]. Moreover, with recent literature, methanolic leaf extracts showed thrombolytic and membrane stabilizing activities. Additionally, the extracts did not show any antibacterial activity as the test sample was resistant against both gram-positive and gram-negative bacteria [45]. Ghosh et al. [160] identified compounds comprising of alkaloids, carbohydrates, tannins, phenolic compounds, sterols, triterpenoids, saponins, and flavonoids from the ethanolic leaf extracts.

### 10.3. Aegiceras corniculatum

*A. corniculatum* originates from the Primulaceae family with a height of up to 7 m. It has alternate and obovate leaves, 3–10 cm long and 1.5–5 cm wide. Its fruit is green to pink in color and has a curved-cylindrical shape [43]. Folk medicinal practitioners from the Sindh region in Pakistan use the stem to treat rheumatism, painful arthritis, and inflammatory diseases [74,75]. *A. corniculatum* showed many potential pharmacological activities (Table 6). For instance, the in vivo antinociceptive activity was investigated by Roome et al. [74,75] using acetic-acid induced writhing in mice. The ethyl acetate stem extracts at 50 mg/kg showed an inhibition of 53 ± 3.0% while the hexane stem extract at the same concentration has an inhibition of 28 ± 2.5%. Janmanchi et al. (2017) conducted an antibacterial study using REMA assay. It was found that the crude leaf extract was active against *Bacillus subtilis* and *Escherichia coli*. With the same extract, the antioxidant study was conducted using DPPH assay, and the IC_50_ value was 1.79 ± 0.0002 mg/mL. However, Roome et al. [74] mentioned that *A. corniculatum* lacked pharmacological evaluation concerning its analgesic effects. Using standard phytochemical tests, analysis of the bark, stem, and leaf extracts showed the presence of alkaloids, amino acids, benzoquinones, tannins, coumarins, flavonoids, saponins, and glycosides, among others (Table 8).

### 10.4. Acrostichum aureum

This species is found in Kerala, India. The local people used the whole plant as a worm remedy and as an astringent for hemorrhage [76]. Thomas [76] investigated the antibacterial activity of methanol, acetone, petroleum ether, and water leaf extracts against *Escherichia coli*, *Serratia marcesens*, *Pseudomonas aeruginosa,* and *Staphylococcus aureus*. The petroleum ether and water leaf extracts were inactive against *Escherichia coli* and *Serratia marcesens* while acetone leaf extract was active against all the tested microorganisms. In the methanol, acetone, petroleum ether, and water leaf extracts, alkaloids were reported absent whereas flavonoids and phenols were found present [76].

### 10.5. Avicennia

*A. marina* is a medium length mangrove tree from the Acanthaceae family with a height of 14 m with a specialized root structure known as pneumatophores. Its bark is smooth with thin, stiff, and brittle flakes in the surface and is generally light grey. The leaves are thick and glossy and 5–8 cm long. The species produces green and oval-shaped fruits [7,46]. *A. marina* is widely distributed in Australia, South-East Asia, Madagascar, Mozambique, and along the coastline of Africa [7]. This species traditionally used to manage smallpox, skin diseases, ulcers, and throat pains [65] and it has many pharmacological properties such as antimicrobial, anti-inflammatory, antiviral, antimutagenic, anticancer, and antioxidant (Table 6). Ramanathan [114] investigated the antimicrobial activity of the crude leaf extract using disc diffusion assay against *S. aureus*, *Klebsiella aerogenes*, *P.s aeruginosa*, *Bacillus subtilis*, *E.a coli*, *Enterobacter aerogenes*, *Proteus* sp, *Salmonella parathyphi,* and *Citrobacter* sp. The extracts showed activity against all the tested microorganisms. Shafie et al. (2013) conducted the anti-inflammatory activity on the rat model, and it was observed that the inflammatory markers were reduced, and the joint lesions were also improved. The ethanolic leaf extracts were active against HIV (Human immunodeficiency virus), SFV (Semliki forest virus), EMVC (Encephalmyocarditis virus), and HBV (Hepatitis B virus) [98]. The phytochemical screening of the methanolic, ethanolic, ethyl acetate, ethyl ether, and water extracts indicated the presence of a wide array of constituents viz; alkaloids, 31 glycosides, phenols, 5 terpenoids, saponins, 14 flavonoids, 23 tannins, 19 naphthalene derivatives, 6 fatty acids, and 7 steroids and amino acids [7] (Table 8).

*A. germinans* is the tallest mangrove tree compared to the other *Avicennia* species such as *A. integra*, *A. bicolor*, *A. marina*, *A. officinalis,* and *A. schaeurina*. This species comes from the Acanthaceae family. It is 30 to 50 m tall with rough and irregular scales on the bark. These plants have opposite and elliptical leaves which are 3–15 cm long. It produces dark-green, flat propagules with velvety pericarp which are 2–3 cm in diameter [7]. The bark, leaf, and flower of *A. germinans* are used traditionally to treat malaria, haemorrhoids, rheumatism, swellings, throat pains, and hemorrhage [7,77]. In the Bahamas, *A. germinans* is traditionally used to restore vitality and to manage rheumatism while in Colombia, gargling the bark decoction helps to cure cancer of larynx and ulcers of the throat [77]. The methanol extract of *A. germinans* exhibited significant antibacterial activity against *Escherichia coli*, *Klebsiella* sp, *Proteus* sp, *Staphylococcus aureus*, *Pseudomonas* sp., and *Salmonella* sp. Fennell et al., (2004) reported that the antibacterial properties are due to the presence of tannins, alkaloids, flavonoids, terpenoids, or essential oils. The compounds identified from *A. germinans* originate from the phytochemical class of glycosides namely 2′-cinnamoyl-mussaenosidic acid (**108**), 2′-caffeoyl-mussaenosidic acid, and 2′-CoU mamaheswarraoroyl-mussaenosidic acid [7,161,162].

*A. integra* is the smallest mangrove tree with a height of 2–7 m in the *Avicennia* genus. It has pneumatophore roots system with smooth bark, brown to reddish. The leaves are opposite, simple, and elliptical with shiny surfaces of length 5–14 cm. The plant produces pale green fruits, 21–23 mm long and 12–15 mm wide. This species also blooms to produce golden yellow or orange zygomorphic flowers. The plant can be found along the coastline of Australia [7].

*A. bicolour* originates from the Acanthaceae family and is 8–20 m tall. It is widely distributed in Colombia, Costa Rica, El Salvador, Guatemala, Honduras, Mexico, Panama, and Nicaragua [7].

*A. schauerina* species comes from the same family of Acanthaceae as the other *Avicennia* mangrove trees. The produced fruits are pale sap green with a purple tinge and are flatter compared to *A*. *germinans*. *A. schauerina* produced flowers which are larger than the flowers produced by *A. bicolor* [7].

*A. officinalis* is 30 m tall with pneumatophore roots system, smooth bark which is dirty green to dark grey, and is slightly fissured but does not flake compared to *A. germinans* [47]. The leaves are shiny, green in color with round apex, 10 cm long, and 5 cm wide. The tree has pneumatophores similar to the other *Avicennia* species. The flower of *A. officinalis* is the largest of all the species in its genus and is orange-yellow to lemon-yellow. This species produced a heart-shaped propagule, green or brown [7,47]. *A. officinalis* is an evergreen mangrove tree distributed throughout India, Bangladesh, Indonesia, Brunei, Myanmar, Vietnam, and Southern Papua New Guinea (Hossain et al., 2016). In Bangladesh, the species is known as ‘DholaBaen’. Locally, it is used as a treatment for boils and tumors (Hossain et al., 2016) and the unripe seeds are poulticed onto the sores of smallpox, boils, and abscesses [156]. Additionally, the bark can be used to heal scabies (Hossain et al., 2016). A decoction of the plant mixed with sugar candy and cumin is used against dyspepsia. The local people traditionally use the resin produced by the plant as a contraceptive without side effects [80]. *A. officinalis* is largely studied for its pharmacological activities. For instance, the ethyl acetate leaf extract is analyzed for its antimicrobial activity against *E. coli*, *Streptococcus mutans*, *S. aureus*, *Aspergillus flavus,* and *Trichophyton rubrum*. The extract showed activity against *E. coli*, *S. mutans,* and *S. aureus* but found inactive for *A. flavus* and *T. rubrum*. Anti-ulcer activity was investigated on the ethanolic extract using indomethacin-induced gastric ulcer assay and it was observed that the gastric ulcers decreased when the amount of glutathione is reduced in the gastric mucosa [115]. Hossain et al. (2012) investigated the diuretic and neuropharmacological properties of the methanolic leaf extracts. The Lipschitz diuretic model was used to test the diuretic activity of the sample. For the dosage of 200 and 400 mg/kg, the volume of urine excreted was 3.06 ± 0.18 mL and 3.89 ± 0.13 mL, respectively. It is reported that the amount of Na^+^ ion excreted by the methanolic extract is higher compared to the excretion of K^+^ ion and as a result the plant is classified as a good diuretic which causes less hyperkalaemic side effects. In GC/MS analysis, the methanolic, ethanolic crude leaf extract showed the presence of alkaloids, terpenoids, glycosides, tannins, steroids, flavonoids, naphthalene derivatives, reducing sugar, sterols, fatty acids, gums, wax esters, and amino acids, among others. Thatoi et al. [7] identified 17 compounds from the terpenoid class of constituents as taraxerol (**96**), taraxerone (**97**), betulinic acid (**98**), betulin (**99**), betulinaldehyde (**125**), β-amyrin (**47**), rhizophorin-A (**133**), rhizophorin-B (**134**), *ent*-13*S*-2,3-seco-14-labden-2,8-olide-3-oic acid (**135**), ribenone (**136**), *ent*-16-hydroxy-3-oxo-13-epi-manoyl oxide (**137**), *ent*-15-hydroxy-labda-8,13*E*-dien-3-one (**138**), *ent*-3α,15-dihydroxylabda-8,13*E*-diene (**139**), excoecarin A (**140**), *ent*-beyerane (**141**), rhizophorin-B (**134**); nine glycosides compounds as 7-*O*-*trans* cinnamoyl-4-epilogenin (**142**), geniposidic acid (**107**), 2′-cinnamoyl-mussaenosidic acid (**108**), 10-*O*-5-phenyl-2,4-pentadienoyl-geniposide (**111**), 7-*O*-cinnamoyl-8-epiloganic acid sodium salt (**143**), 8-*O*-cinnamoylmussaenosidic acid (**144**), officinosidic acid (**145**), loganin C, (**146**) iridoid glucoside; five steroids compounds as β-sitosterol (**54**), stigmasterol (**53**), cholesterol (**51**), campesterol (**52**), stigmast-7-en-3-ol (**55**); four tannins compounds as catechin (**128**), chlorogenic acid (**129**), gallic acid (**130**), ellagic acid (**131**); one naphthalene derivative as avicenol C (**127**); and one flavonoid compound as velutin (**126**) (Table 8).

### 10.6. Bruguiera

This species has not received enough scientific attention concerning its morphological characteristics, traditional uses, and pharmacological properties. However, Revathi et al. [88] and Bandaranayake [65] reported that the stem and bark of the plant consist of sulfur-containing alkaloids.

*B. cylindrica* coming from the Rhizophoraceae family, is 20 cm tall with pneumatophore roots and has smooth bark, grey with corky raised patches containing lenticels. The leaves are glossy in appearance and elliptical in shape with pointed apex. The plant produces fruits of 15 cm long and has a curved-cylinder shape. This species blooms greenish-white flowers in clusters of two to five [48]. The bark of *B. cylindrica* is traditionally used to treat hemorrhage and ulcers by the local people of India [63]. The IC_50_ values for the methanolic leaf and stem extracts are 175 and 162.5 µg/mL, respectively [120]. Laphookhieo et al. [163] conducted phytochemical screening on the fruit of *B. cylindrica* and the pentacyclic triterpenoids esters identified are *E*-feruloyltaraxerol (**147**), 3α-Z-feruloyltaraxerol (**148**), 3β-*E*-feruloyltaraxerol (**149**), 3β-*Z*-feruloyltaraxerol (**150**), 3α-*E*-coumaroyltaraxerol (**151**), and 3α-*Z*-coumaroyltaraxenol (**152**). Gawali and Jadhav [120] reported the presence of tannins, saponins, alkaloids, triterpenoids, anthraquinone, and flavonoids in the leaves of the plant.

The bark of this mangrove tree is used to manage diabetes [61]. Bunyapraphatsara et al. [123] performed antioxidant tests on the ethyl acetate leaf extract using three different methods namely DPPH, lipid peroxidation inhibition, and quinone reductase induction activity. Using the DPPH assay, the resulting EC_50_ values of the young pods and the leaves are 5 and 105 µg/mL, respectively. With lipid peroxidation inhibition assay, the IC_50_ values of the young pods and leaves are 0.375 and 42.6 µg/mL, respectively. Also, while using the third method, which is quinone reductase induction assay, the IC_50_ values recorded were >20 µg/mL for both young pods and leaves samples. Comparing the IC_50_ values from the two methods (lipid peroxidation inhibition and quinone reductase induction), it can be said that the samples showed better inhibition with the quinine reductase induction assay. Arora et al. [101] reported the presence of phenolic compounds in the bark of *B. parviflora*. Revathi et al. [88] reported the presence of tannins and triterpenes in the bark and leaves of the plant.

*B. sexangula* is scarcely distributed on the north shore and sides of Oahu, Hawaii [186]. Revathi et al. [88] reported the presence of phenolics, steroids, alkaloids, and tannins in the bark of this species. Alkaloid (1,2-dithiolane) of this plant exhibited antitumor activity against Sarcoma 180 and Lewis [150].

*B. gymnorhiza* is a common mangrove tree reaching a height of up to 15 m and originates from the Rhizophoraceae family. Its bark is smooth and grey-brown in color. It has smooth and glossy leaves with pointed apex, 9.5–20 cm long and 3–7 cm wide. The propagules are green in color and have a cigar-shape which is 5–12 cm long and 1–2 cm wide. The flowers of the plant have pale yellow-green to pinkish orange sepals [187]. *B. gymnorhiza* is a well-known mangrove tree. This species is distributed in the wild forest of India (Sunderbans), throughout Malaysia, China, Indonesia, Comoros, and Mauritius. In India, the bark and root decoction is used to treat diabetes, fever, and diarrhea [81,82]. In Malaysia, the local people used its stem as a remedy for viral fever [83]. In the Guangxi Province of China, the leaves and fruits are traditionally used to cure burns, intestinal worms, liver disorders, and diarrhea [33,54]. The folk medicine practitioners in Indonesia uses the fruits to treat eye disease, malaria, and shingles, which is a viral infection that can occur anywhere on the body [85]. In Comoros and Mauritius Islands, a decoction is prepared by boiling root (15 cm length) of *B*.*gymnorhiza* and five to seven leaves of *P. borbonense* in two cups of water. The decoction is taken to manage diabetes, hypertension, and hemorrhage [13]. The leaves, roots, and barks of this species have been reported to possess many medicinal properties (Table 5). The methanolic leaf extract has been studied for its antinociceptive activity using acetic acid-induced writhing in mice. At dosage 250 and 500 mg/kg, the % writhing inhibitions were 46% and 59%, respectively. The extract showed significant inhibition compared to the standard drug diclofenac sodium and confirmed the antinociceptive activity [81]. Barik et al. [121] investigated the anti-inflammatory activity on the crude leaf extract using COX (cyclooxygenase) inhibition assay. The %inhibitions at dosage 10 and 10 µg/mL were 9.7 ± 7.2% and 65.1 ± 5.8%, respectively. The ethanolic root extract was reported non-toxic with no significant change in behavior or neurological response up to 400 mg/kg body weight [82]. Methanolic leaf extract was found active against *Escherichia coli* (22 mm), while the hexane bark extract showed a broader spectrum of antimicrobial activity against *K. pneumonia* (23 mm), *S. typhi* (22mm), *Staphylococcus aureus* (19 mm), and *Shigella flexneri* (22 mm), respectively [62]. The plant was also reported to exhibit antioxidant, antihyperglycemic, anti-diarrheal, and hepatoprotective activities [64,81,82,83,105] (Table 7). Phytochemicals present in the leaves, stems, flowers, roots, and fruits include flavonoids, saponins, reducing sugars, tannins, gums, dammarane triterpenes, aromatic compounds, sterols, diterpenoids, anthocyanins, and catechins, among others (Table 8). Rahman et al. [164] identified the compounds from the dammarane triterpenes class as Bruguierol A-C (**153**–**155**), 4-hydroxy-dithiosulfonate, bruguiesulfurol (**156**), 4-hydroxydithiolane 1-oxides, brugierol (**157**), and isobrugierol (**158**).

### 10.7. Ceriops

The whole plant of *C. roxburghiana* species is traditionally used to treat diabetes and ulcers. The tree originates from the Rhizophoraceae family. Phytochemical screening of the whole plant revealed the presence of gibberellins and procyanidins [88].

*C. decandra* is native to India in the Tamil Nadu region. The local people use its bark, flowers, and leaves to treat hepatitis and ulcers [87]. This species showed various pharmacological properties namely antioxidant, anti-inflammatory, anti-microbial, and anti-HIV activities (Table 7). Premanathanet al. (1996) investigated the anti-HIV activity on the leaf extract using MTT (3-(4,5-dimethylthiazol-2-yl)-2, 5-diphenyltetrazolium bromide) assay. The CC_50_ and EC_50_ values of the leaf extract were 216.54 ± 14.21 and 13.38 ± 3.15 µg/mL, respectively. The leaf extract showed an SI value of 16.18. The higher the SI value, the lower the toxicity towards the host cells and, thus, the higher its effects against the virus. Comparing the SI values of the leaf extract of *C. decandra* with the values of *R. apiculata* (9.19), *R. mucronata* (1.62), *R. larmarckii* (2.38), respectively, it can be said that the leaf extract of *C. decandra* has higher anti-HIV activity. The ethyl acetate leaf extract was active against the following bacterial pathogens: *E. coli*, *Agrobacterium tumefaciens*, *S.s mutans,* and *S.s aureus,* while the same extract was found inactive against *A.s flavus* and *T. rubrum* [106]. The ethanolic leaf extract showed antioxidant and anti-inflammatory activities [125]. Revathi et al. [88] reported the presence of polyphenols, tannins, and triterpenes in the bark, leaf, and fruit extracts. Anjaneyulu and Rao [167] identified the following compounds: Ceriopsins F-G (**184**, **185**), *ent*-13-hydroxy-16-kauren-19-oic acid (**186**), methyl *ent*-16β,17-dihydroxy-9(11)-kauren-19-oat (**187**), *ent*-16β,17-dihydroxy-9(11)-kauren-19-oic acid (**188**), *ent*-16-oxobeyeran-19-oic acid (**189**), and 8,15*R*-epoxypimaran-16-ol (**190**) from the ethyl acetate root extract of the plant. Other phytochemicals isolated from the hexane, acetone, chloroform, and methanol leaf extracts include alkaloids, flavonoids, steroids, phenols, saponins, terpenoids, carbohydrates, reducing sugars, and cardiac glycosides, among others (Table 8) [168,169].

*C. tagal* comes from the Rhizophoraceae family and grows to a height of 25 m. It has a buttress root system, smooth barks, and is silvery-grey to orangish-brown with lenticels on its surface. The leaves are obovate and yellowish-green on the bottom surface, and they are 6 cm long and 3 cm wide. The propagule is ovoid in shape, brown in color, and is generally 3 cm long [57]. The bark of this plant is traditionally used to treat hemorrhage [61]. Chen et al. [170] identified six compounds from the root extract as 8(14)-enyl-pimar-2’(3’)-en-4’(18’)-en-15’(16’)-endolabr-16,15,2’,3’-oxoan-16-one (**191**), tagalsin C (**192**), tagalsin I (**193**), lup-20(29)-ene-3β,28-diol (**194**), 3-oxolup-20(29)-en-28-oic acid (**195**), and 28-hydroxylup-20(29)-en-3-one (**196**), while Wang et al. [29] identified 14 compounds from the ethanolic root extract as 3α-*O*-*trans*-feruloylbetulinic acid, 3α-*O*-*trans*-coumaroylbetulinic acid (**205**), 3β-*O*-*cis*-feruloylbetulin (**206**), 3β-*O*-*cis*-coumaroylbetulin (**207**), 3β-*O*-*trans*-coumaroylbetulin (**208**), 3β-*O*-*trans*-feruloylbetulin (**209**), 3β-*O*-*trans*-coumaroylbetulinic acid (**210**), 3β-*O*-*cis*-coumaroylbetulinic acid (**211**), lupeol (**48**), 3-epi-betulinic acid (**212**), betulin (**105**), 3-epi-betulin (**213**), and 28-hydroxylup-20(29)-en-3-one (**196**). Hu et al. [173] also isolated phytochemicals such as Dolabranes (Tagalsins P (**201**), Q (**214**), R (**215**), S (**200**), T (**216**), U (**217**)), pimarane, and abietane (Table 8).

### 10.8. Excoecaria agallocha

This species is also known as the blind-your-eye mangrove plant. This is because the latex produced by the bark is poisonous and can cause temporary blindness. *E. agallocha* comes from the Euphorbiaceae family and is 15 m tall. Its root system is described as elbow shaped pegs. The leaves are alternate and elliptical, with an acuminate apex and narrow base. They are 3–8 cm long and 1.5–3 cm wide [50]. This tree is known to produce latex which has therapeutic effects. The plant is traditionally used to treat rheumatism, epilepsy, leprosy, ulcers, and paralysis. This species is widely distributed from India, Africa, to northwest Australia. The local people of India, New Caledonia, and Malaysia traditionally used the latex and leaves to cure dart and fish poisoning. In Pakistan, besides using it for ulcers, paralysis, rheumatism, and leprosy, the latex is used as an abortifacient [174]. In Tamil Nadu, the latex is also used to alleviate a painful toothache [58]. This mangrove species possesses many pharmacological activities namely antioxidant, anti-inflammatory, analgesic, anticancer, anti-filarial, and antimicrobial activities (Table 7). Mondal et al. [50] investigated the anti-inflammatory activity on the stem extract using two different methods namely carrageenan-induced paw edema test and pellet-induced granuloma test. The ethanol and water (3:1) extract of the plant showed a significant inhibition of 62.29% in carrageenan-induced paw edema model while the pellet-induced granuloma test showed an inhibition of 57.03% with the stem extract. Analgesic activity was tested using acetic acid-induced writhing test in mice. At dosage 500 mg/kg, the ethanol and water (3:1) bark extract showed the highest activity with a reduction of 53.87%. The antimicrobial properties of *E. agallocha* were tested by Bakshi and Chaudhuri [106] using disc-diffusion assay and the results showed the extract was active against *E. coli*, *A. tumefaciens*, *S. mutans,* and *S. aureus* but inactive against *A. flavus* and *T. rubrum*. A study by Mondal et al. [50] revealed a wide array of phytoconstituents isolated from *E. agallocha* (Table 8). The main constituents identified were flavonoids, terpenoids, diterpenes, alkaloids, and tannins [50,88,174,175,176].

### 10.9. Heritiera

*H. fomes* is a tall mangrove tree which can attain a height of 15–25 m. It comes from the Sterculiaceae family and has pneumatophore roots, elliptical leaves, and blooms bell-shaped flowers pink to orange in color [51]. In Bhitarkanika and Sunderbans, India, the leaves, roots, and stems are traditionally used to treat cardiovascular diseases, gastrointestinal disorders (diarrhea, dyspepsia, stomach ache, dysentery, constipation), and skin diseases (rash, eczema, boils, itch, sores) [7,51,89,90]. Ali et al. [91] and Rahmatullah et al. [69] also reported that the whole plant or twig could be used against bloating, diabetes, heart disease, hepatic disorders, goiter, toothache, and oral infection. *H. fomes* showed antihyperglycemic, antidiabetic, and antinociceptive activities in the methanolic bark extract (Table 7). The ethanolic leaf extract showed excellent antimicrobial activity against *E. coli*, *S. typhi*, *S. paratyphi,* and *S. aureus* [51]. Rahmatulla et al. [69] conducted the toxicity test on the leaf extract and the % writhing inhibitions at dosage 250 and 500 mg/kg was 34.83% and 59.20%, respectively. The ethanolic leaf extract, bark extract, and the acetone and aqueous stem extracts were screened for the phytochemical compositions, and the constituents mainly include alkaloids, cardiac glycosides, tannins, steroids, saponins, gums, carbohydrates, proteins, and amino acids, among others (Table 8).

*H. littoralis* comes from the Sterculiaceae family similar to *H. fomes* and reaches a height of 25 m. It forms pneumatophore root and has dark green leaves with acute apex which are generally 10–23 cm long and 4–10 cm wide [51]. This species is broadly distributed in Asia mainly in China, Taiwan, Guangdong, Guangxi, Fujian, and Philippines [92]. In Philippines, the sap is traditionally used to counteract fish, arrowhead, and spearhead poisoning and the seed is used to treat diarrhea, dysentery, and hematuria [92]. Wang et al. [126] studied the antioxidant activities on the leaves and roots using three different assays, namely DPPH, HOm and SO. The IC_50_ value for the leaf extracts for all the three methods are 0.028, 0.600, and 0.606 mg/mL, respectively. Ge et al. [92] also conducted phytochemical screening and isolated four compounds namely 3,5,7-trihydroxychromone-3-*O*-α-l-rhamnopyranoside (**245**), quercetin-3-*O*-α-l-rhamnopyranoside (**246**), (2*R*,3*R*)-dihydroquercetin-3-*O*-α-l-rhamnopyranoside (**247**), and kaempferol-3-*O*-α-l-rhamnopyranoside (**248**) from the ethanolic leaf extract and Revathi et al. [88] reported the presence of alkaloids, tannins, polyphenols, and saponins in the stem, bark, fruit, and leaf extract.

### 10.10. Lumnitzera racemosa

This mangrove tree is used traditionally by the local people of Orissa, India, to treat rheumatism, skin allergies, asthma, diabetes, snake bites, and as a blood purifier [95]. *L. racemosa* showed antioxidant, cytotoxicity, and anticoagulant properties (Table 7). Cytotoxicity test was conducted on the aqueous leaf extract against Hep G2 cancer cell line using MTT assay. The resulting IC_50_ value was 26.05 µg/mL, and the extract was reported to exhibit potent cytotoxicity activity on the Hep G2 cell lines [127]. The aqueous leaf, methanolic twig, and dichloromethane: methanol stem extracts were screened for phytochemicals and most constituents present were alkaloids, phenols, flavonoids, terpenoids, tannins, sterols, carbohydrates, quinines, saponins, quercetin, and aromatic ester [127,167,177].

### 10.11. Kandelia

*K. candel* originates from the Rhizophoraceae family and grows to a height of up to 10 m. It has flaky barks with lenticels on its surface and is reddish-brown. The plant produces oval-shaped fruits, 25 cm long and blooms white flowers [57]. *K candel* is distributed along the tropical and subtropical coastline of China and from western India to Borneo [93]. The plant is traditionally used to treat cardiovascular diseases, cancer, and neurodegenerative disorders. The leaf extract of this mangrove species is reported to possess excellent antioxidant activities. The ethyl acetate hypocotyl extract has an IC_50_ value of 124.19 ± 3.02 µg/mL with DPPH assay and an AAE value of 4.39 ± 3.17 mmol/g with FRAP (Ferric reducing antioxidant power) assay [93].

*K. rheedii* has been used for tuberculosis treatment in India [94]. Revathi et al. [88] reported the presence of steroids and triterpenoids in the bark, leaf, and fruit extracts.

### 10.12. Nypa fruticans

*N. fructicans*, also known as Nypa palm, is a 9-m tall prostate-stemmed gregarious palm originating from the Arecaceae family. The leaves have a palm-like structure. It is distributed in Queensland (Australia), India [180], and Malaysia [179]. This mangrove palm is reported to have received little scientific attention. In Malaysia, the local inhabitants used the plant to manage diabetes [96] and in Philippines, the flowers and leaves are traditionally used to treat diabetes and snake bites [61]. The methanolic leaf extract showed antimicrobial activity against *E. coli*, *A. tumefaciens*, *S. mutans,* and *S. aureus* while the extract was inactive against *A. flavus* and *T. rubrum* [106]. The antioxidant activity of ethyl acetate extract was investigated for its antioxidant activity using DPPH assay, and the result showed an IC_50_ value of 2.770 ± 0.012 mg/mL [96].

### 10.13. Pelliciera rhizophorae

*P. rhizophorae*, also known as tea mangrove, is endemic to the coastline of Central America [44]. It comes from the Tetrameristaceae family. It attains a height of up to 20 m, has a buttress root system, and dark-green, elongated, pointed leaves which are 20 cm long and 5 cm wide [53]. Bioassay-guided fractionation isolated 10 phytoconstituents, namely α-amyrin (**46**), β-amyrin (**47**), ursolic acid (**50**), oleanolic acid (**49**), betulinic acid (**98**), brugierol (**157**), iso-brugierol (**158**), kaempferol (**79**), and quercetin (**12**). The structures of the isolated compounds were determined by two spectroscopic techniques such as APCI-HR-MS and NMR. Oleanolic acid (**49**), kaempferol (**79**), and quercetin (**12**) showed antiparasitic activity against Leishmania donovani, and their respective IC_50_ values were 5.3, 22.9, and 3.4 µM while α-amyrin (**46**) and betulinic acid (**98**) exhibited activity against *Tripanosoma cruzi* and *Plasmodium falciparum* with the corresponding IC_50_ values of 19.0 and 18.0 µM.

### 10.14. Rhizophora

*R. apiculata* comes from the well-known Rhizophoraceae family. It is 30 m tall, has stilt roots, and almost smooth bark. The leaves are decussate, have the acute apex and reddish petiole, are 1.5–3 cm long, and bloom yellow flowers [188]. It is widely distributed across the globe namely Australia, Guam, India, Indonesia, Malaysia, Micronesia, New Caledonia, Papua New Guinea, Philippines, Singapore, Taiwan, Sri Lanka, Maldives, Thailand, Vanuatu, and Vietnam. Folk medicinal practitioners in Tamil Nadu used the whole plant to prevent colitis and inflammatory bowel disease [97] and in Pichavaram region in India, the bark is used to treat amoebiasis, diarrhea, nausea, and vomiting [58]. *R. apiculata* possesses many pharmacological properties namely antioxidant, antimicrobial and anti-HIV activities. The butanol, ethanolic, ethyl acetate, and water stem extracts were tested for the antioxidant activity using DPPH, ABTS, and HO assays [132]. Lim et al. [133] investigated the antimicrobial activity of the crude bark extract using disc diffusion assay. The extract was found active against 11 microorganisms such as *Proteus mirabilis*, *Acinetobacter calcoaceticus*, *S. epidermidis*, *Yersinia enterocolitica*, *S. aureus*, *P. aeruginosa*, *B. cereus*, *E. coli*, *B. subtilis*, *Candida albicans,* and *Cryptococcus neoformans*. However, no fungal activity was reported. Revathi et al. [88] reported the presence of diphatic alcohols, hydrolysable tannins, steroids, triterpenoids, and phenolic compounds in the bark, flower, fruit, and leaf extracts. Gao and Xiao [132] isolated three compounds, namely Lyoniresinol-3α-*O*-β-arabinopyranoside (**258**), Lyoniresinol-3α-*O*-β-rhamnoside (**259**), and Afzelechin-3-rhamnoside (**260**) from the twigs, leaves, and barks. HPLC analysis indicated that the three compounds were mainly found in the bark extract with their respective % as 0.068%, 0.066%, and 0.011%.

*R. mucronata*, also known as red mangrove, loop-root mangrove, or Asian mangrove, is a 20–25 m tall mangrove tree and form part of the Rhizoporaceae family. The species has stilt roots buttressing the trunk. It has dark green thick leaves with a distinct mucronate tip and covered with minute black spots on the inferior surface. The mangrove tree produces green fruits with a cigar shape. The flowers are creamy white in color [13]. The plant is found to be present in many countries across the globe. *R. mucronata* is native to Africa (Egypt, Ethiopia, Kenya, Madagascar, Mauritius, Mozambique, Tanzania, Somalia, South Africa, Sudan), Seychelles island, Asia (India, Papua New Guinea, Sri Lanka, Philippines, Thailand, Taiwan, Vietnam), South Pacific (Solomon Islands, Vanuatu), and Australia (Queensland, Northern Territory). This mangrove species has many beneficial medicinal properties. For instance, in Tamil Nadu, India, the bark or the whole plant is traditionally used to cure angina, dysentery, hematuria, hepatitis, ulcers, diabetes, hemorrhage, vomiting, and nausea. In Mauritius, the local inhabitants use the *R. mucronata* plant as a traditional medicine against diabetes, hypertension, and also as a natural remedy to reduce the level of urea in the blood. A tea is prepared using root (5 cm length) of *R. mucronata*, 3 whole plants of Bidenspilosa, 10 leaves of *P. borbonense*, bark (15 cm length) of *Erythroxylum laurifolium*, 15 leaves of *Aphloia jobi*, and 10 leaves of *Antidesma madagascariense*. The tea is taken to balance the level of urea in the blood [13]. The root decoction is used to manage diabetes and hypertension while the leaf infusion can be used for fever. The Indonesians traditionally sued the whole plant as a treatment for elephantiasis, haematoma, hepatitis, ulcer, and febrifuge [60,61]. In China and Japan, the bark is used against diarrhea [99]. In Papua New Guinea, the local people used the stem to stop constipation, cure fertility and menstruation disorders [59]. *R. mucronata* possesses many pharmacological properties namely antioxidant, anti-inflammatory, anti-bacterial, antimicrobial, antidiabetic, analgesic, anti-HIV, and anti-cholinesterase properties (Table 7). Chakrarborty and Raola [137] conducted the antioxidant study on the crude chloroform leaf extract using DPPH assay and the resulting IC_50_ value was 1.38 ± 0.03 mg/mL, while Suganthy and Devi [100] conducted the same assay to obtain an IC_50_ value of 47.39 ± 0.43 µg/mL. Interestingly, a study conducted by Hardoko [144] reported that the ripe flour of the fruit contains 7.50% soluble dietary fiber and 38.60% insoluble dietary flour. Additionally, the antidiabetic in vivo study conducted showed a decline in the blood glucose level, which, as a result, makes the ripe flour of *R. mucronata* a good functional food for diabetic patients. Recently, Aljaghthmi et al. (2018) showed that the bioactive compounds present in this mangrove species contribute in lowering blood sugar level and boosting insulin production. Pimpliskar et al. (2012) investigated the antimicrobial activity on the ethanolic stem extract. However, to the best of the knowledge of the authors, no other studies were conducted on ripe flour to support or confirm these results obtained by Hardoko [144]. Alikunhi et al. [145] added that the antidiabetic properties of *R.mucronata*, *R.apiculata,* and *R. annamalayana* were due to the presence of the insulin-like protein present in the leaves. *R. apiculata* was more potent compared to the other two *Rhizophora* species since the results were in equivalence with the control drug, glibenclamide. The extract inhibited activity against *E. coli* (16 mm), *S.s aureus* (15 mm), *S. typhi* (20 mm), *S. pyogenes* (12 mm), and *P.s aeruginosa* (15 mm), respectively. However, no inhibition was noted against *K. pneumonia*, *P.s vulgaris,* and *C. albicans*. The different plant parts of *R. mucronata* contain a wide variety of phytochemicals namely condensed tannins, polyphenols, lipids, inositol, gibberellins, alkaloids, tannins, and proteins, among others [58,60,88,137,167] (Table 8).

In India, the bark of *R. conjugata* is used against diabetes [101]. Vadlapuri and Naidu [128] investigated the antimicrobial activity on the crude extract using agar-well diffusion and the extract was active against seven bacterial pathogens namely *Acremonium strictum* (7 mm), *A. flavus* (8 mm), *C. albicans* (11 mm), *S.s mutans* (15 mm), *S. salivarius* (19 mm), *S. aureus* (11 mm), and *Lactobacillus acidophilus* (22 mm), respectively. Activity was highest against *Lactobacillus acidophilus*. Phytochemical screening of the bark and stem extracts showed the presence of anthocyanins, tannins, steroids, and triterpenoids (Table 8).

*R. mangle* comes from the Rhizophoraceae family and attains a height of 24 m. It has stilt roots, thin and smooth bark grey or grey-brown in color. The leaves are elliptical in shape, thick, shiny green on the upper surface, and yellow-green with black spots on the bottom surface [56]. In India, the leaf and bark are used traditionally to manage diabetes [88,101]. *R. mangle* possessed antioxidant and anti-ulcer activities (Table 6). Andrade-Cetto et al. (2017) isolated six compounds from the ethanolic cortex extract, namely Cinchonains Ia and Ib, catechin-3-*O*-rhamnopyranoside, lyoniside, and nudiposide using NMR, UPLC-DAD-MS, HPLC, and the standard TLC techniques. Revathi et al. [88] and Kandil et al., (2004) reported the presence of tannins, triterpenes, flavonoids, glycosides, quercetin, myricetin, and kaempferol diglycosides in the bark and leaf extract.

*R. racemosa* originates from the Rhizophoraceae family. It has a height of up to 30 m, stilt roots, and elliptical leaves [26]. Revathi et al. [88] reported the presence of tannins and steroids in the flower and leaf extract of that plant. This mangrove species has been evaluated for its lethal dose (LD_50_) which is commonly used as a toothache remedy by the Nigerian people [102].

*R. stylosa* is a mangrove tree from the Rhizophoraceae family with a height of up to 15 m. Its bark is dark brown to black. It produces ovoid to pear-shaped propagules and is generally 4 cm long [57]. Li et al. (2010) isolated eight compounds from the crude stem and twig extract namely (–)-epicatechin (**276**), 3-O-acetyl (–)-epicatechin (**277**), 3,3′,4′,5,7-O-pentaacetyl (–)-epicatechin (**278**), (+)-afzelechin (**279**), (+)-catechin (**128**), cinchonain Ib (**268**), and proanthocyanidin B2. Revathi et al. [88] reported the presence of inositols and steroids in the leaves, roots, and seeds extract.

### 10.15. Xylocarpus granatum

This species is a small mangrove plant of height 3–8 m with a buttress root system. It has a light brown, yellowish, or greenish bark, and is smooth and flaky. The leaves are bright light green to dark green with a round apex [24]. The mangrove species occurs mainly in the Indian Ocean and Southeast Asia [185]. In East Africa and South Asia, the local people use the bark and leaf as a natural remedy for cholera, diarrhea, fever, and malaria [58,104]. *X. granatum* has many pharmacological activities namely antioxidant, anticancer, antidiarrheal, and antimicrobial (Table 7). Das et al. [104] investigated the antimicrobial activity on the ethanolic stem extract against seven bacterial pathogens, namely *E. coli*, *E. aerogenes*, *P. aeruginosa*, *S. typhi*, *S. aureus*, *K. pneumonia,* and *V. cholera*. The extract was active against all tested microorganisms. Wu et al. [185] isolated three new limonoids, namely 2,3-dideacetylxyloccensin S (**281**), 30-deacetylxyloccensin W (**282**), and 7-hydroxy-21b-methoxy-3-oxo-24,25,26,27-tetranortirucalla-1,14-diene-23(21)-lactone (**283**) from the seed of the Chinese mangrove, *X. granatum* (Table 8).

## 11. Conclusions and Future Perspectives

This review attempts to project the importance of various mangrove species used traditionally. An overview of their ecological aspects is also given since these plants represent a symbolic plant for the marine ecosystem. The fundamental ecological roles mangroves species play need to be understood to safeguard our environment as these species and their habitats are threatened due to rapid coastal development, extensive aquaculture, climate change scenarios, and overharvesting [9]. So far, there have been piecemeal reviews on mangroves dealing with one aspect or one species at a time but none of them have systematized all the traditionally known mangroves under one review. For instance, Rahmatullah et al. [69] evaluated botanical features and phytochemical profiling of only one mangrove species, *B. gymnorhiza*. Bandaranayake [33,65] reviewed chemical constituents of mangroves, while Mahmud et al. (2014) targeted only one species, *H. fomes*, to evaluate its pharmacological properties and ethnomedicinal uses. Ravindran et al. (2005) [58] reviewed the therapeutic importance and phytochemical screening of one genus, *Rhizophora*, in a book chapter while Shilpi et al., (2012) documented the antinociceptive, anti-inflammatory, and anti-pyretic activities of mangrove plants without detailing on the phytochemical screenings. Kathiresan [189] focused on mangroves from Pichavaram (India) only, while Mondal et al. [50] documented only one species, *E. agallocha*. Patra and Mohanta [190] have reported only the antimicrobial aspects of a few mangroves, and Simlai and Roy [147] elaborated on biological activities and chemical constituents from mangroves in only a specific region of the Sundarban estuary. This review article provides a more extensive coverage on all mangroves by compiling updated information and data on their discovery, ecology and physiological aspects, types, geographical distribution, taxonomy, morphological characteristics, ethnopharmacology, pharmacological activities, and phytochemical evaluation.

Morphologically, mangroves are defined as small trees or shrubs growing along the coastlines in muddy or rocky soils. For instance, Kathiresan and Bingham [6] classified mangroves as halophytes, however Collins, Merriam-Webster, and the Oxford English dictionaries defined mangroves simply as trees or shrubs with tangled roots growing along the coastlines of tropical countries while Spalding [14] generalized mangroves as trees or large shrubs growing in or adjacent to intertidal regions which can easily adapt themselves in their environment. Having said that, it can be concluded that there are still ambiguities in the definition of mangroves and thus require the attention of botanists to properly define the plant. In many countries, particularly in India, people believed that mangroves can cure a wide spectrum of diseases such as rheumatism, diabetes, fever, and gastrointestinal disorders (diarrhea, dysentery, dyspepsia, constipation). Locally, the Mauritian people have used the plant as a traditional medicine for diabetes and hypertension for many years. Interestingly, mangroves are not only important for people but equally significant for animals. For instance, a study conducted by Gardner [16] in Madagascar showed that lemurs use mangroves as their prime natural habitat for sleeping and foraging [16].

From the literature, it is acknowledged that there are 84 mangrove species. However, only 27 species are known to the folklore medicine and not all species have been tested for their pharmacological activities both in vivo and in vitro, which accounts for only about 31% of mangrove species that have been investigated till date. This rather low percentage can be linked to either a poor interest from the researchers’ side on these particular plants or because these plants are considered as endangered species in some countries. Therefore, this might have created a gap between traditional medicines and the interest in developing drugs derived from mangroves. Consequently, to fill the gap between traditional medicines and pharmaceutics, more research is needed to provide a greater range of potential cures against a panel of diseases.

So far, we have seen that mangrove species has a long history in traditional medicine/ethnopharmalogy and is still widely used because of a wide array of potential sources of natural compounds. Several classes of bioactive substances have been isolated and identified and investigations on different metabolic activities have been performed both in vitro and in vivo. While we present and discussed herein evidence in connection with mangrove species and their beneficial medicinal properties, there are still doubts as to how far these bioactive compounds can be used as direct disease management agents. There is no conclusive report of human trials and up to what extent these beneficial medicinal properties are substantiated warrant further investigation. For proper ethnopharmacological use of mangroves, we believe there should be more direct scientific evidence substantiated with more clinical-based research with rationale impact assessed on human health.

A deeper scientific understanding of the mechanisms of those compounds, their molecular targets, and any drug interaction should be further investigated. Well-designed in vivo tests and randomized controlled clinical studies should be carried out to obtain statistically significant outcomes. There is also a dire need to ensure the efficacy and safety of mangrove preparations and not direct their use solely based on people’s perceptions. Other pertinent questions that must be delved in are: How far can these mangroves be further exploited on a commercial scale by pharmaceutical companies? What are the optimized methods of extraction and characterization? What are the risk levels or adverse human effects? What types of pharmacological evaluations must be carried out to confirm activity of mangrove ingredients?

## Figures and Tables

**Table 1:** Local names of mangroves in different countries.

Country	Local Names	Reference
Netherlands	Vloedbosschen (mangrove community), mangrove (individual species)	[6,12,13]
United Kingdom	Mangrove	
France	Manglier, Paletuvier	
Germany	Mangrove	
Madagascar	Honkalahy, Voandrano	
Malaysia	Manggi-manggi	
Mauritius Rodrigues Comoros	Manglier, Paletuvier, Mangrove	
Spain	Manglar	
Surinam	Mangro	

**Table 2:** Distinguishing characteristics between three dominant types of mangroves *.

		Red	Black	White
Characteristics	Leaves	Very shiny, very pointy green on both sides, green on both sides	Less shiny, pointy, grey in color in bottom surface	Shiny on both sides, round
	Roots	Rhizophores or arc-shaped prop roots, roots come out of the stem and grow downwards to end in the soil	Pneumatophores or pencil-like roots, roots grow against gravity from the soil surface	-
	Fruits	Cigar-shaped	Teardrop-shaped	Smallest in size
	Examples	*R. mucronata, R. mangle*	*A. germinans, B. gymnorhiza*	*L.racemosa*

* Source: Restoring Guyanas mangrove ecosystem, 2014 (http://www.mangrovesgy.org/home/index.php/2014-04-27-16-39-08/types-of-mangroves).

**Table 3:** Top 20 mangroves-holding nations in 2014 in km^2^ and percentage of global total.

Rank	Country	km^2^	% Global Total
1	Indonesia	42,278	25.79
2	Brazil	17,287	10.55
3	Malaysia	7616	4.65
4	Venezuela	7516	4.59
5	Nigeria	6908	4.21
6	Papua New Guinea	6236	3.80
7	Colombia	6236	3.80
8	Mexico	6036	3.68
9	Thailand	3936	2.40
10	Gabon	3864	2.36
11	Myanmar	3783	2.31
12	Australia	3314	2.02
13	Panama	2673	1.63
14	Mozambique	2658	1.62
15	Cuba	2407	1.47
16	Bangladesh	2314	1.41
17	Philippines	2084	1.27
18	Ecuador	1906	1.16
19	United States	1554	0.95
20	Cameroon	1323	0.81

**Table 4:** Morphological characteristics of mangroves.

Species	Family	Height	Aerial Roots	Bark	Leaves	Fruits	Flowers	Reference
*Acanthus ilicifolius* L.	Acanthaceae	Up to 2 m	Stilt	-	Spiny edges	Kidney shaped	Large light-violet petals	[42]
*Aegiceras corniculatum* (L.) Blanco	Primulaceae	Up to 6 m	-	Smooth, greyish	Alternate, obovate, 3–10 cm long, 1.5–5 cm wide	Light green to pink, curved cylinder, 2–7.5 cm long	Fragrant, white, clusters of 10–30	[43]
*Aegralitis rotundifolia*	Plumbaginaceae	2–3 m	-	-	Broad ovate, obtuse apex, 5–8.8 cm long, 4.5–8.5 cm wide	-	-	[44,45]
*Avicennia germinans* (L.) L.	Acanthaceae	Up to 30–50 m	Pneumatophores	Rough with irregular flattened scales, dark brown or black	Opposite, elliptical, thick with glands on upper surface, green on upper surface, grey on bottom surface, 3–15 cm long	Dark green, flat, velvety pericarp beneath, 2–3 cm in diameter	White, auxiliary clusters, 1–2 cm in diameter	[7]
*Avicennia integra* N.C.Duke	Acanthaceae	2–7 m	Pneumatophores	Smooth, brown to reddish	Opposite, simple, elliptical, shiny green on upper surface, pale and fine on bottom surface, 5–14 cm long	Pale green, furry, ovoid pods, 21–23 mm long, 12–15 mm wide	Golden yellow or orange, zygomorphic	[7]
*Avicennia bicolour* Standl.	Acanthaceae	8–20 m	-	-	-	-	White corolla with yellow throat, hairy petals, zygomorphic, 5–6 mm in diameter	[7]
*Avicennia marina* (Forssk.) Vierh.	Acanthaceae	Up to 14 m	Pneumatophores	Smooth light grey made up of thin, stiff, brittle flakes	Thick, bright, and glossy on upper leaf, grey or silvery-white on bottom leaf, 5–8 cm long	Green, oval, 20–25 mm in diameter	White or golden yellow, clusters of 3–5	[7,46]
*Avicennia officinalis* L.	Acanthaceae	Up to 30 m	Pneumatophores	Smooth, dirty green to dark grey. Slightly fissured and does not flake	Shiny green with round apex, golden brown on upper leaf, 10 cm long, 5 cm wide	Green or brown, heart-shaped	Orange yellow to lemon yellow, 6–10 mm in diameter	[47]
*Avicennia schauerina* Stapf & Leechm. ex Moldenke	Acanthaceae	-	-	-	-	Pale sap green with purple tinge, flat	Slightly hairy corolla	[7]
*Bruguiera cylindrica* (L.) Blume	Rhizophoraceae	Up to 20 m	Pneumatophores	Smooth and grey, with corky raised patches containing lenticels (pores)	Glossy, elliptical with pointed apex	Curved cylinder, 15 cm long	Greenish white, clusters of 2–5	[48]
*Bruguiera sexangula* (Lour.) Poir.	Rhizophoraceae	Up to 15 m	Pneumatophores	Smooth, grey-brown	Smooth, glossy green with pointed apex, 9.5–20 cm long, 3–7 cm wide	Green, cigar shaped, 5–12 cm long, 1–2 cm wide	Pale yellow-green to pinkish orange sepals	[43]
*Bruguiera gymnorhiza* (L.) Lam	Rhizophoraceae	5–8 m	Pneumatophores	Rough, reddish-brown	Large, dark green, shiny, elliptical in shape with reddish petiole, 3–4.5 cm long	Green, cigar shaped, 2 cm long	Creamy white to brown	[13]
*Ceriops tagal* (Perr.) C. B. Robb.	Rhizophoraceae	Up to 25 m	Buttress	Smooth, lenticels, silvery-grey to orangeish-brown	Opposite in pairs, obovate, yellowish-green on bottom surface, 6 cm long, 3 cm wide	Ovoid, 3 cm long, brown	-	[49]
*Excoecaria agallocha* L.	Euphorbiaceae	Up to 15 m	Elbow-shaped pegs	-	Alternate, elliptical, apex shortly acuminate, narrow base, 3–8 cm long, 1.5–3 cm wide	3-lobed, 8 mm in diameter	Yellow, Unisexual	[50]
*Heritiera fomes* Buch.-Ham	Sterculiaceae	15-25 m	Pneumatophores	-	Elliptical	-	Pink or orange, bell-shaped, 5 mm across	[51]
*Heritiera littoralis* Aiton	Sterculiaceae	Up to 25 m	Pneumatophores	-	Dark green, short petioles of 1 cm, elliptical, acute apex, 10–23 cm long, 4–10 cm wide	Light green to brown	Unisexual	[51]
*Kandelia candel* (L.) Druce	Rhizophoraceae	Up to 10 m	-	Flaky, reddish brown with lenticels	-	Oval, 25 cm long	White	[49]
*Nypa fruticans* Wurmb	Arecaceae	Up to 9 m	-	-	Palm-like	Woody nut	Catkin-like, red or yellow	[52]
*Pelliciera rhizophorae* Planch. & Triana	Tetramerisataceae	Up to 20 m	Buttress	Brown	Dark green, leather-like, smooth on both upper and bottom surface, small hairs on edges, elongated, pointed, 20 cm long, 5 cm wide	Brown, spherical with a pointed end	5-rayed symmetric red or white petals	[53]
*Rhizophora apiculata* Blume	Rhizophoraceae	Up to 30 m	Stilt	Grey, almost smooth, 50 cm diameter	Decussate, rosette-like at end of twigs, acute apex, reddish petiole, 1.5–3 cm long	Brown, ovoid or inversely pear-shaped berry, rough, 2–3.5 cm long	Yellow, bisexual, 4-lobed calyx	[54,55]
*Rhizophora mangle* L.	Rhizophoraceae	Up to 24 m	Stilt	Grey or grey-brown, smooth, thin	Opposite, elliptical, acute apex, thick, shiny green on upper surface, yellow-green, black dots on bottom surface, 6–12 cm long, 2.5–6 cm wide	-	Pale pink	[34,56]
*Rhizophora mucronata* Lam.	Rhizophoraceae	20–25 m	Stilt roots buttressing the trunk	-	Thick, dark green, distinct mucronate tip, covered with minute black spots on inferior surface	Green, cigar-shaped	Creamy-white	[13]
*Rhizophora racemosa* G. Mey	Rhizophoraceae	Up to 30 m	Stilt	-	Opposite, elliptical, hairless blades	-	-	[26])
*Rhizophora stylosa* Griff.	Rhizophoraceae	Up to 15 m	-	Dark brown to black	-	Ovoid to pear-shaped, 4 cm long	-	[57]
*Xylocarpus granatum* J. Koenig	Meliaceae	3–8 m	Buttress long	Light brown, yellowish or greenish, smooth, flaky	Bright light green to dark green, round apex, pinnate	-	White, 8 mm across	[24]

**Table 5:** Traditional uses of mangrove species.

Species	Region/Country	Plant Part(s)	Use(s) in Traditional Medicine	References
*Acanthus ilicifolius* L.	Bangladesh	WP	Aphrodisiac, rheumatism, relief for asthma, diabetes, diuretic, dyspepsia, leprosy, hepatitis, blood purifier, cure for cold, gangrenous wounds, skin allergies, snake bites	[33,65]
	West Bengal	NI	Analgesic, wound healing effect	[66]
	NI	L	Pain reliever	[67]
	Sundarbans, India	L	Rheumatism, neuralgia, snake bite, paralysis, asthma	[68]
	NI	WP	Aphrodisiac, astringent, rheumatic pain, leucorrhea	[69]
	Pichavaram, India	F	Snake bites	[58]
		WP	Detoxification, kidney stone, small pox, skin diseases, ulcer	[70]
	South Thailand	NI	Rheumatism, asthma, paralysis, psoriasis, leucorrhea	[71]
	Thailand	L	Blood purifier, dressing against snake bites, rheumatism	
*Aegialitis rotundifolia* Roxb.	NI	L	Pain reliever, inflammation treatment, anti-ache agent	[72]
	Bangladesh	L	Antidote for insect bites, pyrexia	[73]
*Aegiceras corniculatum* (L.) Blanco	Sindh, Pakistan	St	Rheumatism, painful arthritis, inflammation	[74]
	Sindh, Pakistan	NI	Inflammatory diseases	[75]
*Acrostichum aureum* L.	Kerala, India	WP	Astringent in hemorrhage, worm remedy	[76]
*Avicennia germinans* (L.) L	NI	B, L, F	Astringent, malaria, hemorrhoids, treatment for hemorrhage, rheumatism, swellings, throat ailments	[7]
	NI	NI	Diarrhea, hemorrhage, rheumatism, hemorrhoids, tumors, swellings	[77]
*Avicennia marina* (Forssk.) Vierh.	NI	B, L	Small pox, skin diseases, treatment for ulcers, throat pains	[65]
	Iran	L	Ulcers, rheumatism, burns	[78]
*Avicennia officinalis* L.	Tamil Nadu, India	F	Tumor, boil	[79]
		S	Inflammation, ulcer	
		R	Aphrodisiac	
		B	Skin disease (scabies), contraceptive, astringent, hepatitis	
		Re	Snake bite, wound healing, contraceptive	
	Tamil Nadu, India	L	Asthma, paralysis, dyspepsia, rheumatism, ulcer, snake bite, skin disease, small pox sores, tumor	[80]
	Pichavaram, India	L	Asthma, bronchial, detoxification, joints pain, stomach disorders, urinary disorders	[58]
*Bruguiera cylindrica* (L.) Blume	NI	B	Hemorrhage, ulcers	[63]
*Bruguiera gymnorhiza* (L.) Lam	Sundarbans, India	B, L	Diarrhea, fever	[81]
	India	B, R	Diabetes, viral fever	[82]
	Selangor, Malaysia	St	Burns, intestinal worms, liver disorders	[83]
	Guangxi Province, China	L	Diarrhea	[54,65]
	China	F	Shingles, eye disease, malaria	[84]
	Indonesia	F	Angina, hemorrhage, hematuria	[85]
	South Andaman Island	L, R	Eye diseases, shingles	[62]
		F	Diarrhea, malaria, burns	
		B, R, L	Diabetes, hemorrhage, hypertension, stings of toxic lagoon fish	[13]
	Comoros, Mauritius	R	Eye disease	[86]
	Pichavaram forest, India	L	Constipation	[58]
	Pichavaram, India	WP	Diarrhea, fever, burns, intestinal worms	[64]
	NI	B, L, F		
*Bruguiera parviflora* (Roxb.) Wight & Arn. ex Griff.	NI	B	Diabetes	[61]
*Ceriops decandra* (Griff.) W. Theob.	Tamil Nadu, India	B, F, L	Hepatitis, ulcers	[87]
*Ceriops roxburghiana* Arn.	NI	WP	Diabetes, ulcers	[88]
*Ceriops tagal* (Perr.) C. B. Rob.	NI	B	Hemorrhage	[61]
*Excoecaria agallocha* L.	NI	NI	Epilepsy, ulcers, leprosy, rheumatism, paralysis	[50]
	Pichavaram, India	La	Toothache	[58]
*Heritiera fomes* Buch.-Ham.	Bhitarkanika, India	L, R, S	Cardiovascular diseases, gastrointestinal disorders, skin diseases, hepatic disorders, gastrointestinal disorders (diarrhea, dysentery, constipation, stomach ache, dyspepsia), skin diseases (rash, eczema, boils, itch, sores, scabies), infections, jaundice, hepatitis, wound healing, diabetes, goiter (hyperthyroidism)	[7,89,90]
	Sundarbans, India	WP		[51]
	Sundarbans, India	WP	Heart disease, bloating, stomach ache, diabetes, pain, diarrhea, skin disease, hepatic disorders, goiter	[91]
	NI	T	Toothache, oral infection	[69]
*Heritiera littoralis* Aiton	Philippines	Sa	Fish, arrowhead, and spearhead poisoning	[92]
		S	Diarrhea, dysentery, hematuria	
*Kandelia candel* (L.) Druce.	NI	NI	Cardiovascular disease, cancer, neurodegenerative disorders	[93]
*Kandelia rheedii* Wight & Arn.	India	NI	Tuberculosis	[94]
*Lumnitzera racemosa* Willd.	Orissa, India	NI	Snake bites, rheumatism, skin allergies, blood purifier, asthma, diabetes, anti-fertility	[95]
*Nypa fruticans* Wurmb	Malaysia	NI	Diabetes	[96]
	Philippines	F, L	Diabetes, snake bite	[61]
*Rhizophora apiculata* Blume	Tamil Nadu, India	WP	Prevent colitis, inflammatory bowel disease (IBD)	[97,98]
	Pichavaram, India	B	Amoebiasis, diarrhea, nausea, vomiting	[58]
*Rhizophora mucronata* Lam.	India	WP	Angina, dysentery, hematuria, hepatitis, ulcers, diabetes, hemorrhage	[98]
	Tamil Nadu, India	B	Diarrhea, nausea, vomiting, amoebiasis, antiseptic, stop bleeding	[58]
	Mauritius	L, R	Astringent, antidote against toxic fish stings, diabetes, fever, hypertension	[13]
	Porong, Indonesia	WP	Elephantiasis, hematoma, hepatitis, ulcer, febrifuge	[60,61]
	India	L, R	Angina, blood in urine, diabetes, diarrhea, dysentery, fever	[99]
	Malaysia	L, R	Childbirth, hemorrhage	[100]
	China	B	Diarrhea	
	Japan	B	Diarrhea	
	NI	L	Astringent, antiseptic	
	NI	WP	Diarrhea, elephantiasis, hematuria	[33]
	New Guinea	St	Constipation, cure fertility, menstruation disorders	[59]
	Pichavaram, India	B	Diarrhea, nausea, vomiting	[58]
	Thailand	B	Diarrhea, dysentery, leprosy	[71]
*Rhizophora conjugata* L.	India	B	Diabetes	[101]
*Rhizophora mangle* L.	India	B, L	Diabetes	[88,101]
*Rhizophora racemosa* G. Mey.	Nigeria	L	Toothache, dysmenorrhea	[102]
	NI	NI	Malaria	[103]
*Xylocarpus granatum* J.Koenig	NI	NI	Cholera, diarrhea, elephantiasis, inflammation, pain, swelling of breasts	[104]
	East Africa	B	Cholera, diarrhea, fever, malaria	
	South East Asia	L	Diarrhea	
	Indian coastal region	F	Diarrhea, dyslipidemia, hyperglycemia	
	Pichavaram, India	B	Cholera, diarrhea, dysentery	[58]
	Thailand	B	Cholera	[71]

B = Bark, F = Fruit, La = Latex, L = Leaves, Re = Resin, R = Root, Sa = Sap, S = Seed, St = Stem, T = Twig, WP = Whole plant, NI = Not indicated.

**Table 6:** Traditionally used and pharmacologically validated species of mangroves.

Species	Traditionally Used	Pharmacologically Validated
*Acanthus ilicifolius*	✓	✓
*Aegiceras rotundifolia*	✓	✓
*Aegiceras corniculatum*	✓	✓
*Acrostichum aureum*	✓	✓
*Avicennia germinans*	✓	✓
*Avicennia marina*	✓	✓
*Avicennia officinalis*	✓	✓
*Bruguiera cylindrica*	✓	✓
*Bruguiera gymnorhiza*	✓	✓
*Bruguiera parviflora*	✓	✓
*Bruguiera sexangula*	✓	✓
*Ceriops decandra*	✓	✓
*Ceriops roxburghiana*	✓	✓
*Ceriops tagal*	✓	✕
*Excoecaria agallocha*	✓	✓
*Heritiera fomes*	✓	✓
*Heritiera littoralis*	✓	✓
*Kandelia candel*	✓	✓
*Kandelia rheedii*	✓	✕
*Lumnitzera racemosa*	✓	✓
*Nypa fruticans*	✓	✓
*Pelliciera rhizophorae*	✕	✓
*Rhizophora apiculata*	✓	✓
*Rhizophora mucronata*	✓	✓
*Rhizophora stylosa*	✕	✓
*Rhizophora conjugata*	✓	✓
*Rhizophora mangle*	✓	✓
*Rhizophora racemosa*	✓	✓
*Xylocarpus granatum*	✓	✓
Total number of species	27	27

✓ represents either ‘used’ or ‘validated’, ✕ represents either ‘not used’or ‘not validated’.

**Table 7:** Pharmacological activities of different mangrove species.

Species	Plant Part(s)	Extract	Study/Assays	Activity	Reference
*Acanthus ilicifolius* L.	L, R	Me	Antioxidant-DPPH (In vitro)	IC_50_ (mg/mL): L = 2501.53 ± 182.62, R = 1319.66 ± 150.76	[105]
	L, R	Me	Antioxidant-FRAP (In vitro)	AAE (mg/g): L = 1.10 ± 0.03, R = 1.62 ± 0.03	
	L	Me	Antinociceptive- Acetic acid-induced writhing test (In vivo)	Control (10 mL/kg) number of writhings = 51.5 ± 4.1, at 250 and 500 mg/kg (extract), %inhibition = 33.0% and 51.1% respectively	[68]
	L	Me	Antinociceptive-Formalin test (In vivo)	At 250 and 500 mg/kg, %inhibition = 37.54 and 50.18 respectively for 5 min and 45.5% and 67.24% respectively for 30 min	
	L	Me	Anti-inflammatory- Carrageenan-induced paw edema (In vivo)	ED_50_ (mg/kg) = 146.2, 95% Cl = 69.38–286.2 both at early and late phases. After 2 h, ED_50_ (mg/kg) = 194, 95% Cl = 135.8–301.4. With BW755C (COX-LOX inhibitor) the paw edema decreased significantly. No significant inhibitory activity was shown with indomethacin	[66]
	L	Me	Anti-inflammatory- Acetic acid-induced peritoneal inflammation (In vivo)	At 200 and 400 mg/kg, %inhibition = 48 and 77, respectively	
	L	Me	Antioxidant-DPPH (In vitro)	IC_50_ (g/mL): extract = 8.40 ± 0.06, Quercetin = 5.28 ± 0.08, Vitamin C = 6.62 ± 0.05	
	L	Me	Antioxidant- ABTS (In vitro)	IC_50_ (g/mL): extract = 10.34 ± 0.02, Quercetin = 3.60 ± 0.03, Vitamin C = 4.86 ± 0.03	
	L	Me	Antioxidant- SO (In vitro)	IC_50_ (g/mL): extract = 78.12 ± 2.51, Quercetin = 30.19 ± 1.32, Vitamin C = 52.18 ± 3.14	
	L	Me	Antioxidant- HO (In vitro)	IC_50_ (g/mL): extract = 24.60 ± 1.10, Quercetin = 14.32 ± 0.52, Vitamin C = 21.08 ± 0.34	
	L	A	Antimicrobial (In vitro)	Zone of inhibition (mm) against BS = 20, SA = 18, PA = 18, CA = 22	[67]
	L	Bu		Zone of inhibition (mm) against BS = 16, SA = 8, PA = 10, CA = 15	
	L	C		Zone of inhibition (mm) against BS = 22, SA = 21, PA = 20, CA = 26	
	L	A	Antimicrobial-Disc diffusion assay (In vitro)	Active against EC, AGT, STM, SA, AF, and TR. Zone of inhibition (mm) = 7.5 ± 0.4, 8 ± 0.5, 7 ± 0.1, 8.2 ± 0.3, 8.0 ± 0.7 and 7.9 ± 0.3, respectively. Me and EA extracts are inactive against TR	[106]
*Aegialitis rotundifolia* Roxb.	L	Aq	Anti-inflammatory- Cotton pellet-induced granuloma (In vitro)	At 400 mg/kg, %inhibition = 29.1, while %inhibition of standard drug = 63.22%	[73]
	L	Aq	Anti-inflammatory- Carrageenan induced hind paw edema (In vitro)	At 400 mg/kg, %inhibition = 26.75%, while %inhibition of indomethacin = 40.13%	
	L	Aq	Analgesic- Acetic acid induced writhing test (In vitro)	At 200 and 400 mg/kg, %inhibition = 47.86% and 57.1% respectively	
	L	Aq	Antipyretic (In vitro)	At 400 mg/kg, a moderate antipyretic activity is reported by decreasing the temperature at 36.61 °C	
	L	Aq	Cytotoxicity using micro culture tetrazolium assay (MTT assay) (In vitro)	Active; IC_50_ at 200 µg/mL = 97.77	[107]
	L	Me	Thrombolytic activity (In vitro)	At dosage 2, 4, 6, 8, and 10 mg/mL, %of clot lysis = 9.57 ± 1.06%, 13.35 ± 1.67%, 19.35 ± 1.84%, 28.23 ± 1.97%, and 32.76 ± 1.22%, respectively	[45]
	L	Me	Membrane stabilizing activity―Hypotonic solution-induced hemolysis (In vitro)	At dosage 2, 4, 6, 8, and 10 mg/mL, %inhibition of hemolysis = 22.80 ± 0.49%, 30.80 ± 0.6%, 35.30 ± 0.74%, 40.80 ± 0.89%, and 45.80 ± 0.77%, respectively	
	L	Me	Antibacterial―Disc diffusion (In vitro)	Active against 100 µL of ST and EC. Inactive against SA and PA	
*Aegiceras corniculatum* (L.) Blanco	St	H	Toxicity (In vivo)	Non-toxic at 1 g/kg	[74]
	St	EA		LD_50_(mg/kg) = 850	
	St	Me		Toxic above 200 mg/kg	
	St	EA	Antinociceptive- Acetic acid-induced writhings in mice (In vivo)	At 10 and 50 mg/kg, %inhibition = 29 ± 2.5% and 53 ± 3.0%, respectively, IC_50_ (mg/kg) at 50 mg/kg = 52 ± 4.2	
	St	H		At 25, 50, and 100 mg/kg, %inhibition = 12 ± 0.7%, 28 ± 2.5%, and 37 ± 3.5%, respectively	
	St	Me		At 1, 5, and 10 mg/kg, %inhibition = 33.4 ± 3.3%, 55.6 ± 6.2%, and 82.4 ± 7.3%, respectively. Me extract at 5 mg/kg is more potent with IC_50_ value of 4.2 ± 0.99	
	AP	H	Anti-inflammatory- Carrageenan induced paw edema in rats (In vivo)	At 10, 25, and 50 mg/kg, % inhibition = 15.8 ± 2.0%, 39.2 ± 3.9%, and 65.0 ± 4.0%, respectively	[75]
	AP	EA		At 1, 5, and 10 mg/kg, % inhibition = 28.4 ± 4.7%, 40.6 ± 2.1%, and 51.4 ± 2.7%, respectively	
	AP	Me		At 100 mg/kg, % inhibition = 10.8 ± 3.4%	
	L	CE	Antibacterial using REMA assay (In vitro)	Active against BS (gram-positive) and EC (gram-negative) at 5 mg/mL	[108]
	L, Sb, R	Me	Antioxidant-FRAP (In vitro)	AAE (mg/g) for the 3 methanolic extracts of each plant parts = 5.31 ± 0.11, 8.18 ± 0.14, and 5.03 ± 0.73, respectively	[105]
	L, Sb, R	Me	Antioxidant-DPPH (In vitro)	IC_50_ (mg/mL) for the 3 methanolic extracts of each plant parts = 129.95 ± 3.29, 96.74 ± 2.52, and 233.53 ± 56.25, respectively	
	L	EA	Antimicrobial-Disc diffusion assay (In vitro)	Zone of inhibition (mm) against EC, AGT, STM, and SA = 6.9 ± 0.4, 8.25 ± 0.3, 6.5 ± 0.5, and 8.0 ± 0.4, respectively, Inactive against AF and TR	[106]
*Acrostichum aureum* L.	L	Me	Antibacterial-Disc diffusion (In vitro)	Zone of inhibition (mm) against EC = 10 ± 0.12, SM = 7.6 ± 0.58	[76]
	L	Ac		Zone of inhibition (mm) against PA, SA, EC and SM = 12.3 ± 0.23, 9.7 ± 0.48, 10.6 ± 0.14, and 7 ± 0.32, respectively	
	L	PE		No activity observed	
	L	W		No activity observed	
*Avicennia marina* (Forssk.) Vierh	L	A	Antimicrobial- Agar well diffusion (In vitro)	Active against BC, EF, SA, SM, and AT	[7]
	L	E	Anti-inflammatory- Rat model of rheumatoid arthritis (In vivo)	Inflammatory markers were observed to be reduced and joint lesions were improved	[109,110]
	L	E	Antiviral (In vitro)	Active against HIV, SFV, EMVC, and HBV	[98]
	L	E	Antimutagenic- MTT assay (In vitro)	Strong effect with inhibition rates of 68% and 71% with and without metabolic activation S9	[111]
	L	E	Anticancer- MTT assay (In vitro)	Significant cytotoxic effect on HL-60 cells and induced apoptosis in HL-60 cell line	
	NI	Me	Antioxidant- ABTS (In vitro)	Strong activity	[112]
	L	NI	Antimicrobial (In vitro)	Zone of inhibition (mm) against EC, SA, BS, CA, and AN = 12, 6, 7, 9, and 10, respectively for 30 µl of extract	[113]
	L	Ac	Antimicrobial- Disc diffusion assay (In vitro)	Zone of inhibition (mm) against AGT, STM, SA, and TR are 6.8 ± 0.9, 7.5 ± 0.5, 9.1 ± 0.3, and 6.5 ± 0.35, respectively. Inactive against EC and TR	[106]
	L	CE	Antimicrobial- Disc diffusion assay (In vitro)	Zone of inhibition (mm) against SA, KP, PA, BS, EC, ENA, PS, SP, and CS = 18, 24, 26, 16, 27, 8, 12, 5, and 1, respectively	[114]
	L	CE	Antioxidant- DPPH (In vitro)	%radical scavenging = 88.93%	
*Avicennia germinans* (L.) L.	L	Me	Antibacterial- Disc diffusion assay (In vitro)	At 100 mg, zone of inhibition (mm) against EC, KS, PS, and SA = 16, 22, 12, and 18	[77]
*Avicennia officinalis* L	L	E	Antioxidant- DPPH (In vitro)	IC_50_ (control) = 65.12 ± 54, IC_50_ (extract) at 0.1 mg/mL = 40.77 ± 3.43	[80]
	L	E	Antioxidant- HO (In vitro)	IC_50_ (control) = 64.35 ± 1.34, IC_50_ (extract) = 38.23 ± 3.84	
	L	E	Antioxidant- NO (In vitro)	At 0.1 mg/mL, IC_50_: control = 62.97 ± 8.64, extract = 39.87 ± 4.78	
	L	E	Antioxidant- ABTS (In vitro)	At 0.1 mg/mL, IC_50_: control = 61.84 ± 1.33, extract = 38.78 ± 9.62	
	L	EA	Antimicrobial- Disc diffusion assay (In vitro)	Zone of inhibition (mm) against EC, STM, and SA = 7.8 ± 0.7, 7 ± 0.1, and 7.7 ± 0.5, respectively, inactive against AF and TR	[106]
	R	A, E, Me	Antimicrobial- Agar well diffusion (In vitro)	For the three extracts activity observed with EC, SA, ENA, KP, PA, BS, LD, and SP	[112]
	NI	E	Antiulcer- Indomethacine-induced gastric ulcer (In vitro)	Gastric ulcers observed to decrease when glutathione is reduced in the gastric mucosa	[115]
	L	Me	Anti-inflammatory- Carrageenan induced paw edema (In vivo)	Inhibition of prostaglandin effect more potent in chronic model than in acute model	[79]
	L	Me	Diuretic- Lipschitz dirutic model (In vivo)	At dosage 200 and 400 mg/kg, volume of urine = 3.06 ± 0.18 and 3.89 ± 0.13 mL, respectively	[116]
	L	Me	Neuropharmacological- Pentobarbital induced hypnosis test (In vivo)	At dosage 250 and 500 mg/kg, total sleeping time = 6.74 ± 2.83 and 82.07 ± 3.57 min, respectively while with control (0.1% Tween 80), time = 32.06 ± 1.20 min	
	L	Me	Neuropharmacological- Open field test (In vivo)	At dosage 250 mg/kg, number of movements before and after drug administration after 90 min = 110.50 ± 2.12 and 41.85 ± 3.35, respectively	
				At dosage 500 mg/kg, number of movements before and after drug administration after 90 min = 107.99 ± 2.70 and 30.06 ± 2.64, respectively	
	L	Me	Neuropharmacological- Hole cross test (In vivo)	At dosage 250 mg/kg, number of holes crossed before and after drug administration after 90 min = 7.57 ± 0.18 and 5.30 ± 0.69, respectively	
				At dosage 500 mg/kg, number of movements before and after drug administration after 90 min = 6.61 ± 0.72 and 4.90 ± 0.67, respectively	
	L	PE	Anti-HIV- Reverse transcriptase (RT) inhibition assay (In vitro)	%inhibition: control = 71.04 ± 1.94, extract = 74.79 ± 3.47	[117]
	L	E		%inhibition: control (AZT) = 71.04 ± 1.94, extract = 82.00 ± 0.26	
	Fr	E	Antioxidant- ABTS (In vitro)	Activity highest with ABTS compared to DPPH and FRAP	[112]
	L	E	Toxicity (In vivo)	No significant change observed in the majority of the mice. Mortality rate was zero	[115]
	L	E	Antioxidant- DPPH (In vitro)	At dosage 10 and 100 µg/mL, %inhibition = 16.34% and 63.64%, respectively	[118]
	L	E	Cytotoxic (In vitro)	LC_50_ (µg/mL) = 131.2	
	L	E	Antibacterial- Disc diffusion (In vitro)	Active against EC and ST, MIC (µg/mL) against EC = 62.5, ST = 125	
*Bruguiera cylindrica* (L.) Blume	St	Bu, C, E, H, Aq	Antioxidant- Oxygen free radical generation (In vitro)	%inhibition for all extracts ranged from 18–77 for superoxide anions (O^2-^), 29–43 for hydroxyl radical (OH^•^) and 20–39 for microsomallipid peroxidation	[119]
	L, St	Me	Antioxidant- DPPH (In vitro)	IC_50_ (µg/mL) for L =1 75, St = 162.5	[120]
*Brugueira gymnorhiza* (L.) Lam	L	Me	Antinociceptive- Acetic acid-induced writhing in mice (In vivo)	At dosage 250 and 500 mg/kg, % writhing inhibition = 46% and 59%, respectively. Control (25 mg/kg) = 63%	[81]
	L	Me	Anti-diarrheal (In vivo)	Latent period (h) for control (loperamide) and at dosage 500 mg = 1.71 ± 0.145 and 1.67 ± 0.163, respectively	
	L	CE	Anti-inflammatory- COX inhibition assay (In vitro)	%inhibition at 10 and 100 µg/mL = 9.7 ± 7.2 and 65.1 ± 5.8, respectively	[121]
	L	CE	Antioxidant- DPPH (In vitro)	%inhibition at 2 and 1 mg/mL = 68% and 59%, respectively	
	B	C, E, Me	Antioxidant- DPPH (In vitro)	IC_50_: C = 0.27 ± 0.017, E = 0.029 ± 0.004, Me = 0.038 ± 0.003	[83]
	L	Me	Antimicrobial (In vitro)	Zone of inhibition (mm) against BC, SA, EC, and PA are 12.67, 14.34, 8.87, and 7.85, respectively	
	B	Me	Toxicity (In vivo)	Zone of inhibition (mm) against BC, SA, EC, and PA are 15.86, 17.85, 9.25, and 8.38, respectively	
	R	E		Non-toxic, no significant change in behavior or neurological response up to 400 mg/kg body weight	[82]
	R	E	Antihyperglycemic- STZ induced diabetic rats (In vivo)	Serum glucose levels of control and extract (400 mg/kg) at day 0 = 224.70 ± 15.52 and 237.0 ± 15.0 mg/mL, respectively	
				Serum glucose levels of control and extract (400 mg/kg) at day 7 = 214.5 ± 2.60 and 188.10 ± 3.14 mg/mL, respectively	
				Serum glucose levels of control and extract (400 mg/kg) at day 28 = 201 ± 16.32 and 89.04 ± 10.23 mg/mL, respectively. A significant decrease is observed in the blood glucose level compared to diabetic control rats	
	L	Me	Antimicrobial (In vitro)	Zone of inhibition (mm) against EC= 22	[62]
	B	H		Zone of inhibition (mm) against KP, ST, SA and SF are 23, 22, 19 and 22 respectively	
	L	Me	Antioxidant (In vitro)	IC_50_ (µg/mL) for FRAP, DPPH, NO, SO, HO and ABTS radical scavenging = 17.93 ± 0.161, 0.355 ± 0.005, 0.305 ± 0.004, 0.356 ± 0.007, 0.311 ± 0.004 and 0.056 ± 0.0003 respectively	[64]
	L	Me	Hepatoprotective- GaIN induced hepatic toxicity in rats (In vivo)	With sample GaIN + extract (125 mg/kg), ALT, AST, AKP, and total protein were exhibited to be 76.6 ± 2.75, 79.3 ± 2.49, 121 ± 3.19, and 4.46 ± 0.12. With sample GaIN + extract (250 mg/kg), ALT, AST, AKP, and total protein were exhibited to be 68.8 ± 2.27, 69.1 ± 1.66, 108.8 ± 3.43, and 5.01 ± 0.11	
	L, Sb, R	Me	Antioxidant- FRAP (In vitro)	AAE (mg/g) for the 3 methanolic extracts of each plant parts = 1.25 ± 0.03, 2.85 ± 0.09, and 1.55 ± 0.16, respectively	[105,122]
	L, Sb, R	Me	Antioxidant- DPPH (In vitro)	IC_50_ (mg/g) for the 3 methanolic extracts of each plant parts = 2052.20 ± 172.01, 254.69 ± 21.26, and 1532.71 ± 46.32, respectively	
	NI	Me	Cytotoxicity (In vivo)	IC_50_ ˃2.5 mg/mL	
*Bruguiera parviflora* (Roxb.) Wight & Arn. ex Griff	L	EA	Antioxidant- DPPH (In vitro)	EC_50_ (µg/mL) = 105.00	[123]
	L	EA	Antioxidant- Lipid peroxidation inhibition (In vitro)	IC_50_ (µg/mL) = 42.60	
	L	EA	Antioxidant- Quinone reductase induction activity (In vitro)	CD (µg/mL) ˃ 10, IC_50_ (µg/mL) ˃ 20	
*Bruguiera sexangula* (Lour.) Poir.	L	EA	Antibacterial- Agar diffusion (In vitro)	Inhibition against SA and PS	[124]
*Ceriops decandra* (Griff.) W. Theob.	L, Sb, R	Me	Antioxidant- FRAP (In vitro)	AAE (mg/g) for the 3 methanolic extracts of each plant parts = 0.90 ± 0.66, 13.04 ± 0.75 and 9.81 ± 0.87 respectively	[105]
	L, Sb, R	Me	Antioxidant- DPPH (In vitro)	IC_50_ (mg/g) for the 3 methanolic extracts of each plant parts = 5666.86 ± 324.46, 65.55 ± 1.35, and 93.65 ± 3.52, respectively	
	B	E	Anti-inflammatory- Carrageenan-induced paw edema test (In vivo)	%inhibition of extract (400 mg/kg)= 67.72 while that of standard drug, indomethacin is 69.29%	[125]
	B	E	Antioxidant- DPPH (In vitro)	IC_50_ (µg/mL) = 12.90	
	L	EA	Antimicrobial- Disc diffusion assay (In vitro)	Zone of inhibition (mm) against EC, AGT, STM, and SA are 8.3 ± 0.5, 9.0 ± 0.8, 7.8 ± 0.2, and 8.5 ± 0.45, respectively, Inactive against AF and TR	[106]
*Ceriops roxburghiana* Arn.	L	NI	Anti-HIV- MTT assay (In vitro)	CC_50_ (µg/mL) = 216.54 ± 14.21, EC_50_ (µg/mL) = 13.38 ± 3.15, SI = 16.18	[87]
*Excoecariaa gallocha* L.	L	Me	Antioxidant (In vitro)	IC_50_(µg/µl): DPPH = 67.50, NO inhibition = 4.8, lipid peroxidation inhibition = 100, metal chelating effect(µg) = 2.47	[50]
	La, L, S	E	Anti-inflammatory- Carrageenan-induced paw edema test (In vivo)	%inhibition at 500mg/kg for all 3 extracts are 63.15%, 62.15%, and 69.29%, respectively	
	S	NI	Anti-inflammatory- Pellet-induced granuloma test (In vivo)	At dosage 500 mg/kg, activity was highest with a %reduction of 57.03%.	
	B	E	Analgesic- Acetic acid-induced writhing test in mice (In vivo)	At dosage 500 mg/kg, activity was highest with a %reduction of 53.87%	
	St	E	Anticancer- MTS assay (In vitro)	IC_50_(µg/mL) = 4 and 7, strong activity against pancreatic cancer cell lines Capan-1 and Miapaca-2	
	L	Me	Antifilarial (In vitro)	Significant activity against metazoan filarial parasite Setariadigitata. After 24h treatment with extracts at a concentration of 10, 50, and 100 µg/mL, developmental stages of parasite were found dead with 30%, 75%, 100%, respectively	
	L	EA	Antimicrobial- Disc diffusion assay (In vitro)	Zone of inhibition (mm) against EC, AGT, STM, and SA = 10.3 ± 2.7, 6.2 ± 0.8, 8.3 ± 1.2, and 8.5 ± 0.7, respectively. Inactive against AF and TR	[106]
*Heritiera fomes* Buch.-Ham	B	Me	Antihyperglycemic- Oral glucose tolerance test in glucose-induced Swiss albino mice (In vivo)	After 60 min of glucose loading, serum glucose level with standard drug (glibenclamide- 10 mg/kg) and extract (250 mg/kg) were 43.5 and 49.2, respectively. After 120min of glucose loading, serum glucose level with standard drug, extracts at 250 and 500 mg/kg were 30.1, 35.6, and 44.7 respectively	[91]
	B	Me	Antinociceptive- Acetic acid-induced writhing in mice (In vivo)	At dosage 100, 250, and 500 mg/kg, %inhibition = 8.5, 26.4, and 43.4, respectively	
	L	E	Antioxidant- DPPH (In vitro)	IC_50_(µg/mL) = 26.30	[89]
	L	E	Antinociceptive- Acetic acid-induced writhing test (In vivo)	At dosage 250 and 500 mg/kg, % writhing inhibition = 34.83 and 59.20, respectively	
	L	E	Antimicrobial- Disc diffusion assay (In vitro)	Zone of inhibition(mm) against EC, ST, SP, SD, and SA = 3.92, 7.63, 5.21, 7.54, and 6.41 respectively	
	B	NI	Antidiabetic (In vitro)	After 60 min of glucose loading at dosage 250 mg/kg, serum glucose level was 49.2. After 120 min, serum glucose level of extracts (250 and 500 mg/kg) and standard drug (glibenclamide) were reduced by 35.6, 44.7, and 30.1, respectively	[51]
	L	NI	Antioxidant- DPPH (In vitro)	IC_50_ (µg/mL)= 26.30	
	B	NI	Antioxidant- DPPH (In vitro)	IC_50_(µg/mL) = 22, EC_50_(µg/mL) = 19.4	
	B	NI	Antinociceptive- Acetic acid-induced writhing in mice (In vivo)	At dosage 100, 250, and 500 mg/kg, %writhing inhibitions = 8.5%, 26.4%, and 43.3%, respectively	
	L	NI		At dosage 250 and 500 mg/kg, %writhing inhibitions = 34.83% and 59.20%, respectively	
	L	C	Toxicity (In vitro)	LC_50_(mg/mL) = 234.77 ± 0.144	[69]
	B	Me		LC_50_(mg/mL) = 47.081 ± 0.056	
	L	NI	Antioxidant-DPPH (In vitro)	IC_50_ (µg/mL) = 13	[109]
*Heritiera littoralis* Aiton	L, R	NI	Antioxidant- DPPH (In vitro)	IC_50_(mg/mL): L = 0.028, R = 0.023	[126]
	L, R	NI	Antioxidant- HO (In vitro)	IC_50_(mg/mL): L = 0.600, R = 0.536	
	L, R	NI	Antioxidant- SO (In vitro)	IC_50_(mg/mL): L = 0.606, R = 0.802	
*Kandelia candel* (L.) Druce	Hy	EA, PE, Aq	Antioxidant- DPPH (In vitro)	IC_50_(µg/mL): EA = 124.19 ± 3.02, PE = 153.48 ± 3.22, W = 132.04 ± 2.16	[93]
	Hy	EA, PE, Aq	Antioxidant- FRAP (In vitro)	AAE(mmol/g): EA = 4.39 ± 3.17, PE = 2.99 ± 0.27, W = 3.69 ± 0.04	
*Lumnitzera racemosa* Willd.	L	Aq	Antioxidant- DPPH (In vitro)	IC_50_(µg/mL) = 38.89	[127]
	L	Aq	Antioxidant- ABTS (In vitro)	IC_50_(µg/mL) = 44.38	
	L	Aq	Cytotoxicity against Hep G2 cancer cell line using MTT assay (In vitro)	IC_50_(µg/mL) = 26.05; exhibited potent cytotoxicity activity on Hep G2 cell lines at different concentrations	
	L	Aq	Anticoagulant- APTT and PT assays (In vitro)	Clotting time ratio at concentration 100, 500, and 1000 µg/mL for APTT assay are 1.2, 1.4, and 1.6, respectively. Clotting time ratio at concentration 100, 500, and 1000 µg/mL for PT assay are 1.25, 1.31, and 1.34, respectively. Prolongation of APTT is slightly higher than that of the PT assay	
*Nypa fruticans* Wurmb	NI	EA	Antioxidant- DPPH (In vitro)	IC_50_(mg/mL) = 2.770 ± 0.012	[96]
	NI	Aq	Antidiabetic- Intraperitoneal glusoce tolerance test (In vivo)	Blood glucose lowering effect = 56.6%, serum insulin level = 79.8%	
	L	Me	Antimicrobial- Disc diffusion assay (In vitro)	Zone of inhibition (mm) against EC, AGT, STM, and SA = 6.5 ± 0.4, 7.3 ± 0.5, 6.25 ± 0.3, and 6.8 ± 0.3, respectively. Inactive against AF and TR	[106]
*Pelliciera rhizophorae* Planch. & Triana	L	NI	Antiparasitic (In vitro)	At 10 µg/mL, IC_50_ (µM) for LD, PF, and TC = 12.6 ± 0.2, 9.7 ± 0.3, and 13.0 ± 0.4, respectively. Inactive against VC	[44]
	L	NI	Antidiabetic- α-glucosidase inhibition (In vitro)	More potent against - α-glucosidase than acarbose (positive control) with IC_50_(µM) = 217.7	
*Rhizophora conjugata L.*	NI	CE	Antimicrobial- Agar well diffusion (In vitro)	Zone of inhibition (mm) against AS, AF, CA, STM, STS, SA, and LA = 7, 8, 11, 15, 19, 11, and 22, respectively. Activity against LA was highest	[128]
*Rhizophora mangle* L.	B	Aq	Anti ulcer- Indomethacine-induced gastric ulcer (In vivo*)*	At dosage 50, 125, 250, 500, and 750 mg/kg, the lesion indices = 5.2 ± 0.84, 4.5 ± 0.58, 3.25 ± 1.71, 1.6 ± 1.95, and 4.6 ± 0.55, respectively. Lesion index (control-distilled water) = 4.8 ± 0.45.	[129]
	B	Aq	Antioxidant- DPPH (In vitro)	Significant decrease at 250 and 500 mg/kg compared to the control in gastric volume	[130]
	L	NI	Antioxidant- SO (In vitro)	IC_50_(µg/mL) of extract and polyphenolic fraction = 6.7 and 7.6, respectively	
				IC_50_(µg/mL) of extract and polyphenolic fraction = 31.9 and 21.6, respectively. Activity increased as tannins concentration increased	
	L	NI	Antioxidant- DPPH (In vitro)	IC_50_ (µg/mL) = 89.83 ± 4.91	[131]
			Antioxidant- FRAP (In vitro)	AAE (mmol/g) = 12.98 ± 1.20	
*Rhizophora apiculata* Blume	R	NI	Antioxidant- DPPH (In vitro)	IC_50_ (µg/mL) = 17	[109]
	St	Bu, E, EE, Aq	Antioxidant- DPPH (In vitro)	IC_50_ (µg/mL): Bu = 9.68 ± 1.86, E = 19.31 ± 1.56, EE = 13.56 ± 1.79, W = 23.72 ± 1.94, control (BHT) = 52.20 ± 1.57	[132]
	St	Bu, E, EE, Aq	Antioxidant- ABTS (In vitro)	IC_50_ (µg/mL): Bu = 1.26 ± 0.05, E = 3.01 ± 0.75, EE = 1.71 ± 0.39, W = 4.32 ± 0.96, control (BHT) = 9.63 ± 0.15	
	St	Bu, E, EE, Aq	Antioxidant- HO (In vitro)	IC_50_ (µg/mL): Bu = 9.07 ± 0.99, E = 17.93 ± 1.51, EE = 13.57 ± 1.59, W = 33.59 ± 1.66, control (BHT) = 45.58 ± 2.14	
	B	CE	Antimicrobial- Disc diffusion (In vitro)	Activity tested with MT. Complete inhibition with PM, AC, SE, YE, SA, PA, and BC. Partial inhibition with EC, BS, CA, and CN. No fungal activity reported	[133]
	B	Me		Activity tested with CT. Complete inhibition with SS, SA, PA, and SC. Partial inhibition with PM, SM, SP, BL, SE, BC, ETA, CA, and CN. No fungal activity reported	
				Activity tested with HT. Complete inhibition with PM, AC, SS, AA, BL, SE, ST, SA, and CA. Partial inhibition with PA, BC, ETA, RR, and CN. No fungal activity reported	
				MIC (mg/mL): 1.56 against AC, 3.12 against BC, 6.25 against PA, 6.25 against SA, 3.13 against SS	
	NI	NI	Antioxidant- DPPH (In vitro)	Most potent radical scavengers: catechol, methoxycatechol, syringol. Their respective EC_50_ (mg/mL): 0.1239 ± 0.0004, 0.2001 ± 0.0005, 0.2218 ± 0.0009. EC_50_ (mg/mL) Ascorbic acid (control) = 0.2562 ± 0.0023	[134]
			Antioxidant- FRAP (In vitro)	AEAC (mgAA/g): syringol = 635 ± 35, catechol = 2283 ± 168, methoxycatehol =1560 ± 155	
			Antioxidant- Phosphomolybdenum (In vitro)	AEAC (mgAA/g): syringol = 1556 ± 86, catechol = 1861 ± 95, methoxycatehol = 2396 ± 194	
			Antioxidant- ABTS (In vitro)	TEAC (mgTR/g): syringol = 956 ± 40, catechol = 1022 ± 53, methoxycatechol = 1039 ± 51	
	L	NI	Anti-HIV- MTT assay (In vitro)	CC_50_ (µg/mL) = 998.21 ± 81.57, EC_50_ (µg/mL) = 108.55 ± 16.24, SI = 9.19	[87]
	B	NI	Antioxidant- FRAP (In vitro)	Reducing power increased as concentration of mangrove tannins increased from 20 to 60 µg/mL	[135,136]
			Antioxidant- DPPH (In vitro)	Scavenging activity increased as concentration of tannins increased. Maximum scavenging activity (>90%) exhibited at 30 µg/mL	
	NI	NI	Antimicrobial- Disc diffusion (In vitro)	Zone of inhibition (mm) against BC = 14, SS = 9. For bacteria, AC, KP, BS, SA, BL, SE, BC, SM, PA, MIC (mg/mL) ranged from 3.13 to 386.25	
*Rhizophora mucronata* Lam.	L, Sb, R	Me	Antioxidant- FRAP (In vitro)	AAE (mg/g) for the 3 methanolic extracts of each plant parts =2.89 ± 0.23, 3.62 ± 0.16, and 1.40 ± 0.00, respectively	[105]
			Antioxidant- DPPH (In vitro)	IC_50_ (mg/g) for the 3 methanolic extracts of each plant parts = 365.37 ± 23.95, 193.82 ± 11.14, and 1377.45 ± 50.62, respectively	
	L	C	Antioxidant- DPPH (In vitro)	IC_50_(mg/mL) = 1.38 ± 0.03	[137]
			Antioxidant- ABTS (In vitro)	IC_50_(mg/mL) = 1.25 ± 0.01	
			Anti-inflammatory- COX-1 inhibition (In vitro)	IC_50_(mg/mL) = 1.42 ± 0.01	
			Anti-inflammatory- COX-2 inhibition (In vitro)	IC_50_(mg/mL) = 1.38 ± 0.00	
			Anti-inflammatory- 5-LOX inhibition (In vitro)	IC_50_(mg/mL) = 1.16 ± 0.02, least active with COX-1	
	L	EA	Antibacterial- Agar well diffusion (In vitro)	With 50µl of extract, zone of inhibition(mm) against EC, SA, KP, PV, PA, PSF, ST, and BS = 15, 18, 9, 11, 13, 9, 13, and 6, respectively	[138]
				MIC for EC, SA, KP, PV, PA, PSF, ST, and BS = 8, 9, 8, 15, 8, 13, 11, and 13, respectively	
	R	H	Antimicrobial- Disc diffusion (In vitro)	Zone of inhibition (mm) against BS, SA, PA, PV, CA, AFM, and AN = 20, 16, 19, 17, 16, 17, and 18, respectively	[99]
	R	Me		Zone of inhibition (mm) against BS, SA, PA, PV, CA, AFM, and AN = 16, 14, 16, 16, 14, 12, and 14, respectively	
	L	Me	Antidiabetic- STZ induced diabetic rats (In vivo)	Week 3: FBG(mg/100 mL blood) level at 50 and 100 mg/kg = 90.8 ± 6.03 and 99.3 ± 4.15, respectively Week 10: FBG (mg/100 mL blood) level at 50 and 100 mg/kg = 151 ± 3.26 and 136 ± 5.11, respectively	[64]
	L	Me	Antioxidant – DPPH (In vitro)	IC_50_ (µg/mg) = 5.25 ± 0.039	
	L	Me	Antibacterial-Disc diffusion (In vitro)	Zone of inhibition (mm) against BS, SA, STF, STP, EC, and PA = 9.97 ± 0.17, 19.56 ± 0.19, 15.74 ± 0.06, 11.31 ± 0.25, 5.63 ± 0.06, and 16.57 ± 0.22, respectively	[139]
			Antioxidant- DPPH (In vitro)	%radical scavenging at 4, 8, 16, 32, and 64 µg/mL = 15.1 ± 0.2, 19.82 ± 0.61, 25.98 ± 0.46, 36.98 ± 0.04, and 42.98 ± 0.28, respectively	
			Antioxidant- HO (In vitro)	%radical scavenging at 4, 8, 16, 32, and 64 µg/mL = 19.08 ± 0.14, 22.62 ± 0.35, 25.43 ± 0.18, 28.36 ± 0.22, and 32.77 ± 0.44, respectively	
	L	C	Analgesic (In vivo)	Basal reaction time (s) after 15 min of administration = 7.40 ± 0.30, after 30 min = 11.34 ± 0.05, after 45 min = 13.13 ± 0.03, after 90 min = 9.01 ± 0.28	[140]
	B, F, Fr, L, R	Me	Antibacterial- Disc diffusion (In vitro)	Zone of inhibition (mm) against SA for the respective plant parts extracts = 8.8, 7.5, 7.1, 6.1, and 7.6	[60]
				Zone of inhibition (mm) against EC for the respective plant parts extracts = 6.4, NR, 8.6, 6.2, and 7.1. Highest activity with bark extract for both bacteria	
	L	NI	Anti HIV- MTT assay (In vitro)Antioxidant- DPPH (In vitro)	CC_50_ (µg/mL) = 798.39 ± 72.02, EC_50_ (µg/mL) = 492.29 ± 48.99, SI = 1.62	[87]
	L	Me	Antioxidant- DPPH (In vitro)	IC_50_ (µg/mL) = 47.39 ± 0.43	[100]
			Antioxidant- HO (In vitro)	IC_50_(µg/mL) = 401.45 ± 18.52	
			Antioxidant- NO (In vitro)	IC_50_(µg/mL) = 80.23 ± 0.70	
			Antioxidant- Hydrogen peroxide (In vitro)	IC_50_ (µg/mL) = 316.47 ± 3.56	
			Anti-cholinesterase (In vitro)	%inhibition against AChE = 92.73 ± 0.54, BuChE = 98.98 ± 0.17, IC_50_(µg/mL): AChE = 59.31 ± 0.35, BuChE = 51.72 ± 0.35	
	L	Me	Antioxidant- DPPH (In vitro)	IC_50_(µg/mg) = 5.25 ± 0.039	[64]
			Antioxidant- NO (In vitro)	IC_50_(µg/mg) = 3.44 ± 0.038	
			Antioxidant- SO (In vitro)	IC_50_(µg/mg) = 6.04 ± 0.012	
			Antioxidant- HO (In vitro)	IC_50_(µg/mg) = 5.01 ± 0.072	
			Antioxidant- ABTS (In vitro)	IC_50_(µg/mg) = 1.42 ± 0.009	
	St	E	Antimicrobial (In vitro)	Zone of inhibition (mm) against EC, SA, ST, STP, and PA = 16, 15, 20, 12, and 15, respectively. No inhibition against KP, PV, and CA	[141]
				MIC (mg/mL): EC = 17, SA = 16, ST = 19, CA = 15 at 10 mg/mL of extract	
	L	H, EA, Me	Anti-cholinesterase (In vitro)	IC_50_ (µg/mL): H = NR, EA = NR, Me = 222.48, Physostigmine (control) = 0.06	[142]
	Fr			IC_50_ (µg/mL): H = 3.68x10^-6^, EA = 322.27, Me = 1.01, Physostigmine (control) = 0.06	
	AP	EA	Antimicrobial- Agar disc diffusion (In vitro)	Overall activity (%) against SA, SM, KP, SF, ML, VM = 66.6	[143]
	AP	Me		Overall activity (%) against SA, SM, KP, SF, ML, VM = 100.0	
	AP	C		Overall activity (%) against SA, SM, KP, SF, ML, VM = 14.28	
	Fr	NI	Antidiabetic- Alloxan- induced diabetic rats (In vivo)	Dosage 500, 1000, 1500, 2000 mg/day/head for 18 days were administered into diabetic rats Positive control (glibenclamide: 0.09 mg/day/200 g body weight) Blood glucose level of both groups (control and experimental rat group) decreased	[144]
	L	NI	Antidiabetic- Alloxan- induced diabetic rats (In vivo)	Dosage 60 mg/kg was administered to rats for 30 days. A decrease in blood glucose level was observed	[145]
	L	E	Hypoglycemic effect- Streptozotocin-induced diabetic rats (In vivo)	Dosage 100 and 200 mg/kg were administered for 6h. Positive control (glibenclamide) = 0.5 mg/kg. Higher percentage decrease observed with control (27.2%) compared to 100 mg/kg extract (19.7%), 200 mg/kg extract (21.0%)	[146]
*Rhizophora racemosa* G. Mey	L	Me	Lethal dose evaluation- Karber’s method (In vitro)	LD_50_= 1583.33 mg/kg, the lethal dose is safe to use as a traditional medicine	[102]
*Rhizophora stylosa* Griff.	L	H, EA, Me	Anti-cholinesterase (In vitro)	IC_50_ (µg/mL): H = 715.52, EA = NR, Me = 268.39, Physostigmine (control) = 0.06	[142]
	Fr	H, EA, Me		IC_50_ (µg/mL): H = NR, EA = 2.92, Me = 9.56, Physostigmine (control) = 0.06	
*Xylocarpus granatum* J. Koenig	NI	NI	Antioxidant- DPPH (In vitro)	IC_50_ (µM) = 3.3 ± 0.3	[147]
			Antioxidant- 15LOX (In vitro)	IC_50_ (µM) = 9 ± 1	
			Anticancer (In vitro)	IC_50_ (µM) of 16.93 against CaCo-2 colon cancer cell line	
	B	Me	Antidiarrheal (In vivo)	Significant activity at doses 250 and 500 mg/kg against castor oil and magnesium sulfate induced murine models	
	B, L, Fr	E	Antidiarrheal- Castor oil induced diarrheal model (In vivo)	Active	
	Sb	E	Antimicrobial- Agar disc diffusion *(In vitro)*	Active against EC, ETA, PA, ST, SA VC, and KP	[104]

Ac = Acetone, A = Alcohol, AA = *Acinetobacter anitratus*, AAE = Ascorbic acid equivalent, ABTS = 2, 2-azino-bis-3-ethyl benzthiazoline-6-sulfonic acid radical scavenging, AC = *Acinetobacter calcoaceticus*, AChE = Acetylcholinesterase, AEAC = Ascorbic acid equivalents per gram sample, AGT = *Agrobacterium tumefaciens*, AF = *Aspergillus flavus*, AFM = *Aspergillus fumigatus*, AN = *Aspergillus niger*, AKP = Alkaline phosphatase, ALT = Alanine transaminase, AT = *Aspergillus tumefacians*, AP = Aerial part, APTT = Activated partial thromboplastin, AS = *Acremonium strictum*, AST = Aspartateaminotransferase, Aq = Aqueous extract, B = Bark, BL = *Bacillus licheniformis*, BS = *Bacillus subtilis*, BC = *Bacillus cereus*, BHT = Butylated hydroxyl toluene, Bu = Butanol, BuChE = Butyrylcholinesterase, CA = *Candida albicans*, C = Chloroform, CC = Cytotoxic concentration, CD = Double specific activity, CE = Crude extract, CN = *Cryptococcus neoformans*, COX = Cyclooxigenase, CT = Condensed tannin, CS = Citrobacter sp, DPPH = 1-diphenyl-2-picryhydrazyl, E = Ethanol, EA = Ethyl acetateextract, EE =Ethyl ester, ETA = *Enterobacter aerogenes*, EC = *Escherichia coli*,EF = *Enterococcus faecalis*, ENA = *Enterobacter aerogenes*, EC_50_ = Effective concentration 50, ED_50_ = Effective dose 50,EMVC = Encephalmyocarditis virus, ETA = *Enterobacter aerogenes*, FBG = Fast blood glucose, Fr = Fruit, FRAP = Ferric reducing antioxidant power, GaIN = D-galactosamine, H = Hexane extract, HBV = Hepatitis B virus, HIV = Human immunodeficiency virus, HL-60 = Human leukaemic 60, HO = Hydroxyl, HT = Hydrolysable tannin, Hy = Hypocotyl, IC_50_ = Inhibitory concentration 50, KP = *Klebsiella pneumonia*, KS = Klebsiella sp, L = Leaf, LA = *Lactobacillus acidophilus*, LD = *Lactobacillus delbrueckii*, LDV = *Leishmania donovani*, LOX = lipoxygenase, MDA = Malondialdehyde, Me = Methanol, MIC = Minimun inhibitory concentration, ML = *Micrococcus luteus*, MTT = 3-(4, 5-dimethylthiazol-2-yl)-2, 5-diphenyltetrazolium bromide, MT = Mixed tannin, MTS = Cell proliferation assay, NI = Not indicated, NO = Nitric oxide, NR = No result, PA = *Pseudomonas aeruginosa*, PE = Petroleum ether extract, PF = *Plasmodium falciparum*, PM = *Proteus mirabilis*, PT = Prothrombin time, PSF = *Pseudomanas fluorescens*, PV = *Proteus vulgaris*, PS = Proteus sp, REMA = Resazurin microtitreassay, R = Root, RPA = Raw pyroligeneous acid, RR = *Rhodotorula rubra*, S = Seed, SA = *Staphylococcus aureus*, Sb = Stem bark, SE = *Staphylococcus epidermidis*, SF = *Shigella flexneri*, SFV = Semliki forest virus, SI = Selective index (CC_50_/EC_50_), SC = *Staphylococcus cerevisiae*, SD = *Shigella dysenteriae*, SM = *Serratia marcesens*, SP = *Salmonella paratyphi*, SS = *Staphylococcus saprophyticus*, ST = *Salmonella typhi*, STF = *Streptococcus faecalis*, STM = *Streptococcus mutans*, STP = *Streptococcus pyogenes*, STZ = Streptozotocin, STS = *Streptococcus salivarius*, St = Stem, SO = Superoxide, TC = *Trypanosoma cruzi*, TEAC = mg of Trolox equivalents per gram sample, TR = *Tricophyton rubrum*, VC = *Vibrio cholera*, VC = *Vibrio mimicus*, YE = *Yersinia enterocolitica.*

**Table 8:** Phytochemical constituents of different mangrove species.

Species	Plant Part	Extract	Phytochemical Class	Constituent	Reference
*Acanthus ilicifolius* L.	NI	NI	Aliphatic glycosides	Ilicifolioside B (1), ilicifolioside C (2)	[42]
			Alkaloids	Acanthicifoline (3), trigonelline (4), 2-benzoxazolinone (5), benzoxazin-3-one (6), 5,5′-bis-benzoxazoline-2,2′-dione (7), 4-*O*-β-d-glucopyranosyl-benzoxazolin-2(3H)-one (8), (2*R*)-2-β-d-glucopyranosyloxy-2H-1,4-benzoxazine-3(4H)-one (9), (2*R*)-2-β-glucopyranosyloxy-4-hydroxy-1,4-benzoxazine-3-one (10), 2-hydroxy-2H-1,4-benzoxazin 3(4H) one (11)	
			Flavonoids	Quercetin (12), quercetin 3-*O*-β-d-glucopyranoside (13), apigenin 7-*O*-β-d-glucuronide (14), methylapigenin 7-*O*-β-d-glucopyranuronate (15), acacetin 7-*O*-β-l-rhamnopyranosyl-(1”→6”)-*O*-β-d-glucopyranoside (16), vitexin (17)	
			Lignan glycosides	(+)-lyoniresinol 3a-(2-(3,5-dimethoxy-4-hydroxy)-benzoyl)-*O*-β-glucopyranoside (18), dihydroxymethyl-bis(3,5-dimethoxy-4-hydroxyphenyl)tetrahydrofuran-9(or 9′)-*O*-β-d-glucopyranosid (19), (8*R*,7′*S*,8′*R*)-5,5′-dimethoxylariciresinol-4-*O*-β-d-glucopyranoside (20), alangilignoside C (21), (+)-syringaresinol-*O*-β-d-glucopyranoside (22), (+) lyoniresinol 3-*O*-β-d-glucopyranoside (23), (+)-lyoniresinol 2a-*O*-β-d-galactopyranosyl-3a-*O*-β-d-glucopyranoside (24), (+)-lyoniresinol 3a-*O*-β-d-galactopyranosyl-(1-6)-β-d-glucopyranoside (25), (−)-lyoniresinol 3-*O*-β-d-glucopyranoside (26)	
			Megastigmane and phenolic glycosides	(*Z*)-4-coumaric acid 4-*O*-β-d-glucopyranoside (27), (*Z*)-4-coumaric acid 4-*O*-β-d-apiofuranosyl-(1′’/2′)-*O*-d-glucopyranoside (28), (6*R*,7*E*,9*R*)-9-hydroxy-megastigman-4,7-dien-3-one-9-*O*-β-d-glucopyranoside (29), (6*S*,7*E*,9*S*)-6,9-dihydroxymegastigman-4,7-dien-3-one-9-*O*-β-d-glucopyranoside (30), plucheoside B (31), 2,6-dimethoxy-p-hydroquinone 1-*O*-β-d-glucopyranoside (32), syringic acid-*O*-β-d-glucopyranosyl ester (33), 5,11-epoxymegastigmane glucoside (34)	
			Phenylethanol glycosides	Phenylethyl-*O*-β-d-glucopyranosyl-(1/2)-*O*-β-d-glucopyranoside (35), phenylethyl-*O*-β-d-glucopyranoside (36), cistanoside F (37), isocistanoside F (38), cistanoside E (39), campneoside I (40), ilicifolioside A (41), ilicifolioside D (42), acteoside (43), isoacteoside (44)	
			Triterpenoids	α-l-Arabinofuranosyl-(1/4)-β-d-glucuronopyranosyl-(1_3)-3-hydroxylup-20(29)-ene (45), α-amyrin (46), β-amyrin (47), lupeol (48), oleanolic acid (49), ursolic acid (50)	
			Steroids	Cholesterol (51), campesterol (52), stigmasterol (53), β-sitosterol (54), stigmast-7-en-3-ol (55), 28-isofucosterol (56), octacosyl alcohol (57), sitosterol-3-*O*-β-d-glucopyranoside (58)	
			Fatty acid derivatives	Palmitic acid (59), octadecanoic acid (60), stigmasterol octadecenoate (61), β- tetracosanol (62), octacosanol (63)	
			Miscellaneous	(2*R*)-2-*O*-β-d-glucopyranosyl-4-hydroxy-2H-1,4-benzoxazin-3(4H)-one (64), betaine (65), vanillic acid (66), luteolin-7-*O*-β-d-glucuronide (67), uridine (68), uracil (69)	
	NI	H, Me	Anthraquinone, alkaloids, flavonoids, glycosides, saponins, tannins, terpenoids	NI	[155]
	B, Fr, L, R	NI	Alkaloids, long chair alcohols, steroids, sulphur, triterpenes, saponins	NI	[88]
	L	Me	Protein, resin, steroids, tannins, glycosides, reducing sugar, carbohydrates, saponins, sterols, terpenoids, acidic compounds, phenol, cardio glycosides, catechol	NI	[156]
	L	Me	Flavonoids, tannins, steroids, saponins, glycosides	NI	[68]
	R	E	NI	Erigeside C (70)	[157]
	R	E	Triterpenoid saponin	NI	[158]
	L	Aq	2-benzoxazolinone	NI	
	L	C	Pentacyclic triterpenoids, sterols	NI	
	L	E	Methylapigenin 7-*O*-β-glucoronate-flavone glycosides	NI	
	L	Me	Bisoxazolinone	NI	
	AP	Me	Lignan, cyclolignan glycosides	NI	
	L	E	NI	1β,3β-dihydroxyrs-12-en-28-oic acid (71), 2α, 3β-dihyodroxyurs-12-en-28-oic acid (72), 3β, 19α, 23, 24-tetrahydroxyurs-12-en-28-oic acid (73), ursolic acid (50), chrysosplenol C (74)	[159]
*Aegialitis rotundifolia* Roxb.	L	E	Alkaloids, carbohydrates, tannins and phenolic compounds, steroids, sterols, triterpenoids, saponins, flavonoids	NI	[160]
*Aegiceras corniculatum* (L.) Blanco	B, L, St	NI	Amino acids, benzoquinones, tannins, coumarins, flavonoids, saponins, polyphenols, triterpenoids, steroids, quinines	NI	[88]
	L	Me	Tannins, saponins, glycosides, phenolics, flavonoids	NI	[108]
*Acrostichum aureum* L.	L	Pe	Flavonoids, phenols, sterols, phenol, and polyphenol	NI	[76]
	L	Me, W	Flavonoids, phenols	NI	
*Avicennia marina* (Forssk.) Vierh.	St	NI	Phytoalexins, tannins, triterpenes, steroids	NI	[88]
	L	M, E, EE, EA, W	Alkaloids, glycosides, phenols, steroids, tannins, terpenoids	NI	[7]
	L	M, EA	Saponins	NI	
	L	M, E, EE, EA, W	Flavonoids	Luteolin 7-*O*-methylether (75), chrysoeriol 7-oglucoside (76), isorhamnetin 3-*O*-rutinoside (77), 5-hydroxy-4; 7-dimethoxyflavone (78), quercetin (12), laempferol (79), 4′5-dihydroxy-3′-5,7-diimethoxyflavone (80), 4′,5-dihydroxy-3′,7-trimethoxyflavone (81), 4′,5,7-trihydroxyflavone (82), 3′,4′,5-trihydroxy-7-methoxyflavone (83), 2-(3′-3′-hydroxymethyloxiran-2′-yl-2′ methoxy-4′-Methoxymethylphenyl)-4H chromen-4-one (84)	
	L	M, E, EE, EA, W	Naphthalene Derivatives	Naphtha[1,2-b]furan-4,5-dione (85), 3-hydroxy-naphtha[1C-b]furan- 4,5-dione (86), 2-[2′-2′-hydroxypropyl]-naphtha[1,2-b]furan-4,5-dione (87), avicennone A (88), avicenol A (89), stenocarpoquinone B (90), 7′*S*,8′*R*-4,4′,9′-trihydroxy-3,3′,5,5′- tetramethoxy-7,8-dehydro-9-al-2,7′-cycloligan (91), lyoniresinol (92)	
	L	M, E, EE, EA, W	Tannins	Lapachol (93)	
	L	M, E, EE, EA, W	Steroids	β-sitosterol (54), ergost-6,22-diene-5,8-epidioxy-3β-ol (94), stigmasterol-3-*O*-β-d-galactopyranoside (95)	
	B, L, R	M, E, EE, EA, W	Terpenoids	Lupeol (48), taraxerol (96), taraxerone (97), betulinic acid (98), betulin (99), ursolic acid (50), 6Hα-11,12,16-trihydroxy-6,7-secoabieta-8,11,13-triene-6,7-dial11,6-hemiacetal (100), 6Hβ-11,12,16-trihydroxy-6,7-secoabieta-8,11,13-triene-6,7-dial11,6-hemiacetal (101)	
	L, R	M, E, EE, EA, W	Fatty Acids	Oleic acid (102), linolenic acid (103), palmitic acid (59), stearic acid (104), lauric acid (105), myristic acid (106)	
	B, L	M, E, EE, EA, W	Glycosides	Geniposidic acid (107), 2′-cinnamoyl-mussaenosidic acid (108), mussaenoside (109), 2′-cinnamoyl-mussaenoside (110), 10-*O*-5-phenyl-2,4-pentadienoyl-geniposide (111), 7-*O*-5-phenyl-2,4-pentadienoyl-8-epiloganin (112), 10-*O*-(*E*-cinnamoyl)-geniposidic acid (113), 2′-*O*-(2*E*,4*E*-5-phenylpenta-2,4-dienoyl)mussaenosidic acid (114), marinoids A–E (115–119), verbascoside (120), isoverbascoside (121), derhamnosylverbascosid (122), 11-hydroxy- 8,11,13-abietatriene 12-*O*-β-xylopyranoside (123), lyoniresinol 9′--*O*-β-d-glucopyranoside (124)	
	L	CE	Alkaloids, flavonoids, terpenoids, phenolics, saponins, amino acid	NI	[114]
*Avicennia germinans* (L.) L.	L	NI	Glycosides	NI	[7,161,162]
*Avicennia officinalis* L.	L	Me	Alkaloid, reducing sugar, tannins, gums, flavonoids, steroid	NI	[116]
	L	CE	Alkaloid, flavonoid, terpenoids, phenolics, tannins, sterols, glycosides	NI	[114]
	L	Me	Pentacyclic triterpenoids	Lupeol (48), betulin (99), betulinaldehyde (125), betulinic acid (98), β-sitosterol (54)	[79]
	L	Me	Glycosides, flavonoids, alkaloids, steroids, tannins, wax esters	NI	
	L	NI	Flavonoid	Velutin (126)	[7]
	L	NI	Naphthalene derivatives	Avicenol C (127)	
	L	NI	Tannins	Catechin (128), chlorogenic acid (129), gallic acid (130), elagic acid (131)	
	L	NI	Steroids	β-sitosterol (54), stigmasterol (53), cholesterol (51), campesterol (52), stigmast-7-en-3β-ol (132)	
	L	NI	Terpenoids	Taraxerol (96), saraxerone (97), setulinic acid (98), setulin (99), betulinaldehyde (125), β-amyrin (47), rhizophorins A-B (133-134) *ent*-13S-2,3-seco-14-labden-2,8-olide-3-oic acid (135), ribenone (136), *ent*-16-hydroxy-3-oxo-13-epi-manoyl oxide (137), *ent*-15- hydroxy-labda-8, 13*E*-dien-3-one (138), *ent*-3a,15-dihydroxylabda-8,13*E*-diene (139), excoecarin A(140), *ent*-beyerane (141)	
	L	NI	Glycosides	7-*O*-*trans*-cinnamoyl-4-epilogenin (142), geniposidic acid (107), 2′-cinnamoyl-mussaenosidic acid (108), 10-*O*-5-phenyl-2,4-pentadienoyl-geniposide (111), 7-*O*-cinnamoyl-8-epiloganic acid sodium salt (143), 8-*O*-cinnamoylmussaenosidic acid (144), officinosidic acid (145), loganin C (146),	
	L	E	Carbohydrate, reducing sugar, combined reducing sugar, glycosides, tannins, alkaloids, proteins, terpenoids and flavonoids	NI	[118]
*Bruguiera cylindrica* (L.) Blume	Fr	NI	Pentacyclic triterpenoids esters	*E*-feruloyltaraxerol (147), 3α-*Z*-feruloyltaraxerol (148), 3β-*E*-feruloyltaraxerol (149), 3β-*Z*-feruloyltaraxerol (150), 3α-*E*-coumaroyltaraxerol (151), 3α-*Z*-coumaroyltaraxenol (152)	[163]
	L	NI	Tannins, saponins, alkaloids, triterpenoids, anthraquinone, flavonoids	NI	[120]
*Bruguiera parviflora* (Roxb.) Wight & Arn. ex Griff.	B	NI	Phenolic compounds	NI	[101]
*Bruguiera conjugata* (L.) Merr.	St, B	NI	Sulfur containing alkaloids	NI	[88]
*Bruguiera rumphii* Blume	B, L	NI	Tannins, triterpenes	NI	[88]
*Bruguiera sexangula* (Lour.) Poir.	B	NI	Phenolics, steroids, alkaloids, tannins	NI	[88]
*Bruguiera gymnorhiza* (L.) Lam	L	Me	Flavonoids, saponins, reducing sugars, tannins, gums	NI	[81]
	F	NI	Dammarane triterpenes	Bruguierol A–C (153–155), bruguiesulfurol (156), brugierol (157), isobrugierol (158)	[164]
	St	NI	Pimaren diterpenes	*ent*-8(14)-pimarene-15*R*, 16-diol (159), *ent*-8(14)-pimarene-1alpha, 15*R*,16-triol (160), isopimar-7-ene-15S,16-diol (161), (−)-1β,15(*R*)-*ent*-pimar-8(14)-en-1,15,16-triol (162)	
			Aromatic compounds	1-(3-hydroxyphenyl)-2,5-hexanediol (163), 3,4-dihydro-3-(3-hydroxybutyl)-1,1-dimethyl-1H-2-benzopyran-6,8-diol (164), (4alpha,8beta,13beta)-13-(hydroxymethyl)-16-oxo-17-norkauran-18-al (165), (4alpha,16alpha)-17-chloro-13,16-dihydroxy-kauran-18-al (166),(4alpha)-13,16,17-trihydroxy-kaur-9(11)-en-18-oic acid (167),(4alpha)-16,17-dihydroxy-kaur-9(11)-en-18-al (168), *ent*-Kaurenol (169), *ent*-kaur-16-ene-13,19-diol (170), (−)-kauran-17,19-diol (171), (−)-17-hydroxy-16alpha-kauran-19-oic acid (172),(4α)-16,17-dihydroxy-Kauran-18-al (173),(−)-*ent*-kaur-16-en-13-hydroxy-19-al (174),16,17-dihydroxy-9(11)-kauren-18-oic acid (175)	
	WP	NI	Gibberellin	Gymnorrhizol (176), gibberellin A3 (177), A4 (178), A7 (179)	
	L	NI	Sterols	Cholesterol (51), campesterol (52), stigmasterol (53), 28-isofucosterol (56)	
	R	NI	Diterpenoids	Steviol (180), (−)-*ent*-kaur-16-en-13-hydroxy-19-al (174), 15(*S*)-isopimar-7-en-15,16-diol (181), *ent*-kaur-16-en-13,19-diol (177), methyl-*ent*-kaur-9(11)-en-13,17-epoxy-16-hydroxy-19-oate (182), apiculol (1-hydroxy-epimanoyl oxide) (183)	
	NI	NI	NI	Gymnorrhizol (176)	[165]
	R	Me	Gums, flavonoids, saponins, reducing sugar, tannins	NI	[152]
	Fr	NI	Anthocyanins, catechins, diterpenes	NI	[88]
*Ceriops roxburghiana* Arn.	WP	NI	Gibberellins, procyanidins	NI	[88]
*Ceriops decandra* (Griff.) W. Theob.	B, Fr, L	NI	Polyphenols, tannins, triterpenes	NI	[88]
	L	B, E	Protein, coumarin, phenols, flavonoids, saponins, glycosides, alkaloids, terpenoids, tannins	NI	[80,166]
	R	EA	Diterpenoids	Ceriopsin F, G (184, 185)	[167]
			NI	*ent*-13-hydroxy-16-kauren-19-oic acid (186), methyl *ent*-16β,17-dihydroxy-9(11)-kauren-19-oat (187), *ent*-16β,17-dihydroxy-9(11)-kauren-19-oic acid (188), *ent*-16-oxobeyeran-19-oic acid (189), 8,15*R*-epoxypimaran-16-ol (190)	
	NI	NI	Alkaloids, flavonoids, phenols, saponins, steroids, tannins, terpenoids	NI	[168]
	L	H	Carbohydrates, free reducing sugars, tannins, steroids, cardiac glycosides, terpenoids, flavonoids	NI	[169]
	L	C	Carbohydrates, combined reducing sugars, steroids, cardiac glycosides, terpenoids	NI	
	L	Ac	Carbohydrates, monosaccharides, combined reducing sugars, tannins, free anthraquinones, flavonoids, soluble starch, alkaloids	NI	
	L	Me	Carbohydrates, monosaccharides, combined reducing sugars, tannins, free anthraquinones, flavonoids, soluble starch	NI	
*Ceriops tagal* (Perr.) C. B. Rob.	B	NI	Inositols, steroids, polyphenols, tannins	NI	[88]
	R	NI	Dimeric diterpenoids	8(14)-enyl-pimar-2’(3’)-en-4’(18’)-en-15’(16’)-endolabr-16,15,2’,3’-oxoan-16-one (191)	[170]
	R	NI	Terpenoids	Tagalsin C (192), Tagalsin I (193), lup-20(29)-ene-3β,28-diol (194), 3-oxolup-20(29)-en-28-oic acid (195), 28-hydroxylup-20(29)-en-3-one (196)	
	AP	E	Dolabranes	Tagalsin V (197), Tagalsin W (198)	[171]
	AP	E	Terpenes	*ent*-5α,3,15-dioxodolabr-1,4(18)-diene-2,16-diol (199), tagalsin S (200), tagalsin P (201), *ent*-5α,2,15-dioxodolabr-3-ene-3,16-diol (202), *ent*-8(14)-pimarene-15*R*,16-diol (165), 3a-lup-20(29)-ene-3,28-diol (203)	
	St, Tw	E	NI	Tagalsin H (204)	[172]
	AP	E	Lupane-type triterpenes	3α-*O*-*trans*-coumaroylbetulinicacid (205), 3β-*O*-*cis*-feruloylbetulin (206)	[29]
	AP	E	Triterpenes	3β-*O*-*cis*-coumaroylbetulin (207), 3β-*O*-*trans*-coumaroylbetulin (208), 3β-*O*-*trans*-feruloylbetulin (209), 3β-*O*-*trans*-coumaroylbetulinic acid (210), 3β-*O*-*cis*-coumaroylbetulinic acid (211), lupeol (48), 3-epi-betulinic acid (212), betulin (105), 3-epi-betulin (213), 28-hydroxylup-20(29)-en-3-one (196)	
	St, Tw	NI	Dolabranes	Tagalsin P (201), Q (214), R (215), S (200), T (216), U (217)	[173]
	St, Tw	NI	Pimarane	NI	
	St, Tw	NI	Abietane	NI	
*Excoecaria agallocha* L.	St, Tw	NI	*ent*-kaurane diterpenoids	Agallochaol K (218), L (219), M (220), N (221), O (222), P (223)	[174]
	St, Tw	NI	Atisane-type diterpenoid	Agallochaol Q (224)	
	St, Tw	NI	Diterpenoids	NI	
	B	NI	*ent*-isopimarane-type diterpenoid	NI	[175]
	NI	NI	Diterpenoids	Excoecarins D, E, K (225-227)	[176]
	WP	NI	Alkaloids, tannins, phorbol esters, polyphenols	NI	[88]
	NI	NI	Diterpenoids	3-oxo-*ent*-13-epi-8 (13)-epoxy-15-chloro-14-hydroxylabdane (228), *ent*-15-chloro-13,14-dihydroxylabd-8 (9)-en-3-one (229), *ent*-15-chloro-labd-8 (9) ene-3α,13,14-triol (230), 8,13-epoxy-3-nor-2,3-seco-14-epilabden-2,4-olide (231), *ent*-3β-hydroxy-13-epi-manoyl oxide (ribenol) (232), (13*R*,14*S*)-*ent*-8α,13;14,15-diepoxy-13-epi-labda-3-one (excoecarin B) (233)	[50]
	NI	NI	Triterpenoids	3β-(2*E*,4*E*)-5-oxo-decadienoyloxy-olean-12-ene (34), β-amyrin acetate (235), Taraxerone (197), 3-epitaraxerol (236), taraxerol (196), 3-epilupeol (237), acetylaleuritolic acid (238)	
	NI	NI	Flavonoids	2′,4′,6′,4-tetramethoxychalcone (239), 3,5,7,3′,5′-pentahydroxy-2*R*,3*R*-flavanonol 3-*O*-α-l-rhamnopyranoside (240)	
	NI	NI	Alkaloid	2,4-dimethoxy-3-ψ,ψ-dimethylallyl-*trans*-cinnamoylpiperidide (241)	
	NI	NI	Sterols	β-sitostenone (242), (24*R*)-24-ethylcholesta-4,22-dien-3-one (243)	
	NI	NI	Tannin	3,4,5-trimethoxyphenol 1-*O*-β-d-(6-galloyl)-glucopyranoside (244)	
*Heritiera fomes* Buch.-Ham.	L	E	Flavonoids, tannins, alkaloids, terpenoids, saponins	NI	[89]
	L	NI	Saponins, alkaloids, glycosides, steroids, flavonoids, gum, phytosterols, reducing sugars	NI	[51]
	B	NI	Proanthocyanidins	NI	
	Sb	Aq	Alkaloids, cardiac glycoside, anthraquinone glycoside, tannin, steroids, saponins, flavonoids, gums and mucilages, carbohydrates, proteins and amino acids, terpenoid	NI	[90]
	Sb	Ac	Alkaloids, cardiac glycoside, anthraquinone glycoside, flavonoids, carbohydrates, proteins and amino acids, terpenoid	NI	
*Heritiera littoralis* Aiton	L	E	Flavonoids	3,5,7-trihydroxychromone-3-*O*-α-l-rhamnopyranoside (245), quercetin-3-*O*-α-l-rhamnopyranoside (246), (2*R*,3*R*)-dihydroquercetin-3-*O*-α-l-rhamnopyranoside (247), kaempferol-3-*O*-α-l-rhamnopyranoside (248)	[92]
	St, B, Fr, L	NI	Alkaloids, tannins, polyphenols, saponins	NI	[88]
*Lumnitzera racemosa* Willd.	L	Aq	Phenols, flavonoids, alkaloids, terpenoids, sterols, tannins, carbohydrates, cardiac glycosides, saponins, quinines	NI	[127]
	Tw	Me	Flavonoid, quercetin, myricetin	NI	[177]
	St	CH_2_Cl_2_:Me	Aromatic ester	NI	[167]
*Kandelia candel* (L.) Druce	WP	NI	Alkaloids, tannins, saponins, polyphenols	NI	[88]
	L	C	Carbohydrate, alkaloid, flavonoid, tannin, phenol	NI	[178]
	L	EA	Carbohydrate, alkaloid, glycoside	NI	
	L	E	Carbohydrate, protein, amino acid	NI	
*Kandelia rheedii*	B, Fr, L	NI	Steroids, triterpenoids	NI	[88]
*Nypa fruticans* Wurmb	NI	Aq	NI	Acetic acid (249), 2,3-butanediol (250), 1-(2-butoxyethoxy)-ethanol (251), 5-bromo-2-hydroxybenzaldehyde (252), (4-aminophenyl)-phenylmethanone (253)	[96]
	Fr	Aq	NI	Gallic acid (130), protocatechuic acid (54), 4-hydroxybenzoic acid (255), chlorogenic acid (129), rutin (256), cinnamic acid (257), quercetin (12), kaempferol (79)	[179]
	L	E	Alkaloids, cardiac glycosides, anthranoids, polyphenols, flavonoid	NI	[180]
	Hu	E	Alkaloids, phlobotannins, anthranoids, polyphenols, saponins	NI	
	L, Fr	NI	NI	Acetic acid (249),	[88]
*Pelliciera rhizophorae* Planch. & Triana	L	CE	NI	α-amyrin (46), β-amyrin (47), ursolic acid (50), oleanolic acid (49), betulinic acid (98), brugierol (157), iso-brugierol (158), kaempferol (79), quercetin (12)	[44]
*Rhizophora apiculata* Blume	B, F, Fr, L	NI	Aliphatic alcohols, hydrolysable tannins, steroids, triterpenoids, phenolic compounds	NI	[88]
	Tw, L, B	NI	NI	Lyoniresinol-3α-*O*-β-arabinopyranoside (258), lyoniresinol-3α-*O*-β-rhamnoside (259), afzelechin-3-rahmnoside (260)	[132]
	NI	CE	Alcohols, ketones, furan and pyran derivatives, guaicol and derivatives, phenol and derivatives, syringol and derivatives, pyrocatechol, alkyl aryl ether, nitrogenated compounds, carbohydrate derivatives, carbohydrate derivatives	NI	[134]
*Rhizophora mucronata* Lam.	B, Fr, F, R	NI	Alkaloids, tannins, gibberellins, inositol saponins, lipids	NI	[88]
	NI	NI	Proteins, minerals, carotenoids, hydrolysable tannins, lipids, polysaccharides, steroids, triterpenes, condensed tannins, procyanidins, anthocyanidins, alkaloids, carbohydrates, chlorophyll, gibberellins, flavonoids, inositols, polyphenols, saponins	NI	[181]
	L	C	Oleanenes	olean-18(19)-en-3β-yl-(3,6-dimethyl-3*E*,6*Z*-dienoate) (261), (13α)-27-frido-olean-14(15)-en-(17α)-furanyl-3β-ol (262)	[137]
	L, B, Fr, F	Me	Alkaloid, tannin, saponin, phenolic, flavonoid, terpenoid, steroid, glycosides	NI	[60]
	R	Me	Alkaloid, tannin, saponin, steroids, glycosides	NI	
	R	EA	Diterpenoids	Rhizophorin A (133), rhizophorin B (134), rhizophorin C-E (263–264)	[167]
	B	NI	Lupeol, quercetin, caffeic acid	NI	[154]
	L, F	H	Tannin, saponin, terpenoid, alkaloid, flavonoid	NI	[142]
	AP	CE	Ethanone	1-(2-hydroxy-5-methylphenyl) (265)	[143]
*Rhizophora conjugata* L.	B	NI	Anthocyanins, tannins, steroids, triterpenoids	NI	[88]
	St	E	NI	Lyoniresinol-3α-*O*-β-arabinopyranoside (258), lyoniresinol-3α-*O*-β-rhamnoside (259), afzelechin-3-*O*-l-rhamno-pyranoside (266)	[132]
*Rhizophora mangle* L.	B, L	NI	Tannins, triterpenes	NI	[88]
	L	NI	Flavonoid glycosides, quercetin, myricetin, kaempferol diglycosides	NI	[182]
	Co	E	NI	Cinchonain Ia (267), Ib (268), catechin-3-*O*-rhamnopyranoside (269), lyoniside (270), nudiposide (271)	[183]
	R	H	Diterpenes	Manool (272), jhanol (273), steviol (180)	[184]
			Benzaldehyde	p-oxy-2-ethylhexyl benzaldehyde (274)	
*Rhizophora racemosa* G. Mey.	F, L	NI	Tannins, steroids	NI	[88]
*Rhizophora stylosa* Griff.	St, Tw	CE	Acetylated flavonol	3,7-*O*-diacetyl (−)-epicatechin (275)	[174]
	St, Tw	CE	Flavonol derivatives	(−)-epicatechin (276), 3-*O*-acetyl (−)-epicatechin (277), 3,3′,4′,5,7-O-pentaacetyl (−)-epicatechin (278), (+)-afzelechin (279), cinchonain Ib (268), proanthocyanidin B2 (280)	
	L, R, Se	NI	Inositols, steroids	NI	[88]
	L, F	H	Tannins, saponin, terpenoid, flavonoid	NI	[142]
*Xylocarpus granatum* J. Koenig	Se	NI	NI	2,3-dideacetylxyloccensin S (281), 30 deacetylxyloccensin W (282), 7-hydroxy-21b-methoxy-3-oxo-24,25,26,27-tetranortirucalla-1,14-diene-23(21)-lactone (283)	[185]
	NI	NI	NI	Xyloccensin O (284), xyloccensin P (285), gedunin (286), catechin (141), (−) epicatechin (276), procyanidin B1 (287), procyanidin trimer (288), procyanidin pentamer (289)	[147]

AP = Aerial part, Aq = Aqueous, B = Bark, C = Chloroform, CE = Crude extract, Co = Cortex, CH_2_Cl_2_ = Dichloromethane, E = Ethanol, EA = Ethyl acetate, EE = Ethyl ether, Fr = Fruit, L = Leaf, NI = Not indicated, H = Hexane, Hu = Husk, Me = Methanol, R = Root, Se = Seed, St = Stem, Sb = Stem bark, Tw = Twig, WP = Whole plant.

## Abbreviations

APCI/HR/MS	Atmospheric pressure chemical ionisation high resolution mass spectrometry
ABTS	2,2-azino-bis-3-ethyl benzthiazoline-6-sulfonic acid radical
COX-2	Cyclooxigenase 2
DPPH	1-diphenyl-2-picryhydrazyl
EBSCO	Elton B. Stephens Co
FRAP	Ferric reducing antioxidant power
GC/MS	Gas chromatography mass spectrometry
HRESI/MS	High resolution electrospray ionisation mass spectrometry
HO	Hydroxyl
IC_50_	Inhibitory concentration 50
NR	No result
NI	Not indicated
PROSEA	Plant resources south-east Asia
REMA	Resazurin microtitre assay
SI	Selective index
SO	Superoxide
UPLC/DAD/MS	Ultra high performance liquid chromatography diode array detector tandem mass spectrometry
